# Excited-State Intramolecular Proton Transfer in 2-(2′-Hydroxyphenyl)pyrimidines: Synthesis, Optical Properties, and Theoretical Studies

**DOI:** 10.1021/acsami.2c05439

**Published:** 2022-05-17

**Authors:** Rodrigo Plaza-Pedroche, M. Paz Fernández-Liencres, Sonia B. Jiménez-Pulido, Nuria A. Illán-Cabeza, Sylvain Achelle, Amparo Navarro, Julián Rodríguez-López

**Affiliations:** † Área de Química Orgánica, Facultad de Ciencias y Tecnologías Químicas, Universidad de Castilla-La Mancha, Avda Camilo José Cela 10, 13071 Ciudad Real, Spain; ‡ Dpto. de Química Física y Analítica, Facultad de Ciencias Experimentales, Campus Las Lagunillas, Universidad de Jaén, 23071 Jaén, Spain; § Dpto. de Química Inorgánica y Orgánica, Facultad de Ciencias Experimentales, Campus Las Lagunillas, Universidad de Jaén, 23071 Jaén, Spain; ∥ Univ Rennes, CNRS, Institut des Sciences Chimiques de Rennes - UMR 6226, F-35000 Rennes, France

**Keywords:** ESIPT, pyrimidines, fluorescence, TD-DFT, anticounterfeiting

## Abstract

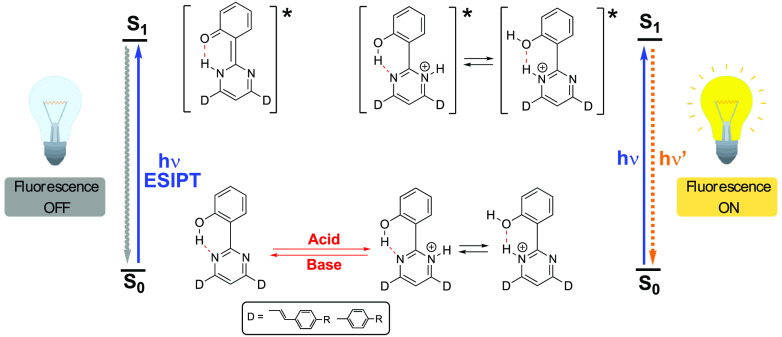

The development of fluorescence materials with *switched on*/*off* emission has attracted great attention owing to the potential application of these materials in chemical sensing. In this work, the photophysical properties of a series of original 2-(2′-hydroxyphenyl)pyrimidines were thoroughly studied. The compounds were prepared by following well-established and straightforward methodologies and showed very little or null photoluminescence both in solution and in the solid state. This absence of emission can be explained by a fast proton transfer from the OH group to the nitrogen atoms of the pyrimidine ring to yield an excited tautomer that deactivates through a nonradiative pathway. The key role of the OH group in the emission quenching was demonstrated by the preparation of 2′-unsubstituted derivatives, all of which exhibited violet or blue luminescence. Single crystals of some compounds suitable for an X-ray diffraction analysis could be obtained, which permitted us to investigate inter- and intramolecular interactions and molecular packing structures. The protonation of the pyrimidine ring by an addition of trifluoroacetic acid inhibited the excited-state intramolecular proton transfer (ESIPT) process, causing a reversible *switch on* fluorescence response detectable by the naked eye. This acidochromic behavior allows 2-(2′-hydroxyphenyl)pyrimidines to be used as solid-state acid–base vapor sensors and anticounterfeiting agents. Extensive density functional theory and its time-dependent counterpart calculations at the M06-2*X*/6-31+G** level of theory were performed to rationalize all the experimental results and understand the impact of protonation on the different optical transitions.

## 1 Introduction

In the last years, the phenomenon of excited -state intramolecular proton transfer (ESIPT) has been widely studied both from a spectroscopic and theoretical point of view,^1−6^ since it is a fundamental process in different chemical and biological systems.^7−10^ Typical ESIPT molecules possess intramolecular hydrogen bonds because the geometric proximity between the proton donor and acceptor units is crucial for ESIPT to occur. ESIPT is usually accompanied by large Stokes shifts, very short lifetimes (*k* ≈ 10^13^ s^–1^), and often low fluorescence quantum yields in solution. Photoexcitation triggers a fast proton transfer from the H-bond donor to the H-bond acceptor that leads to a tautomer (keto) with a different electronic and geometrical structure from the original excited form (enol). As a consequence, ESIPT chromophores are able to show two emission bands, the one with longer wavelength arising from the excited-state keto form (ESIPT emission).^11,12^ Because of the major structural reorganization, the fluorescence properties are highly sensitive to the microenvironment. Thus, the dual emission of the ESIPT molecules is finely tunable and has found numerous applications in fields such as UV photostabilizers,^13,14^ fluorescent probes and imaging agents,^15−19^ and organic optoelectronic devices,^20−25^ among others.

Although the range of ESIPT emitters is wide, 2-(2′-hydroxyphenyl)-substituted derivatives of benzimidazole, benzoxazole, and benzothiazole are by far the most studied to date. In this type of compound, it is also possible to detect triple fluorescence, because luminescent phenolic anions can be generated in alkaline protic media.^2,26−28^ In contrast, diazine-based fluorophores have been scarcely investigated in this context.^29,30^ Recently, the first detailed account for quinazoline derivatives has been reported, in which the ESIPT emission was found to be completely quenched in solution but successfully restored via aggregation-induced emission (AIE).^31^ Frustration of ESIPT luminescence is very common in solution, although it can sometimes be restored in the solid state due to the beneficial restriction of molecular motions.^3^

On the other hand, the photophysical properties of conjugated molecules based on diazines and benzodiazines also respond to environmental stimuli. These molecules have demonstrated to be highly sensitive to changes in polarity, pH, and the presence of metal cations.^32−34^ In this respect, the potential for protonation, complexation, and hydrogen bonding with the nitrogen atoms provides an excellent tool for developing new sensing and luminescent materials.

In this paper, we describe the synthesis, characterization, and a full investigation of the photophysical properties of a series of original 2-(2′-hydroxyphenyl)pyrimidines. Through first-principles calculations, we give an in-depth insight into the ESIPT process and nonradiative deactivation mechanism of this family of compounds. Time-dependent density functional theory (TD-DFT) has shown to be a suitable computational method for a deeper understanding of the ESIPT process,^1,35−44^ in which the proton transfer takes place in the excited state but not in the ground state.

## 2 Results and Discussion

### 2.1 Synthesis of 2-Aryl-4,6-bis(arylvinyl)pyrimidines

**Scheme 1:**
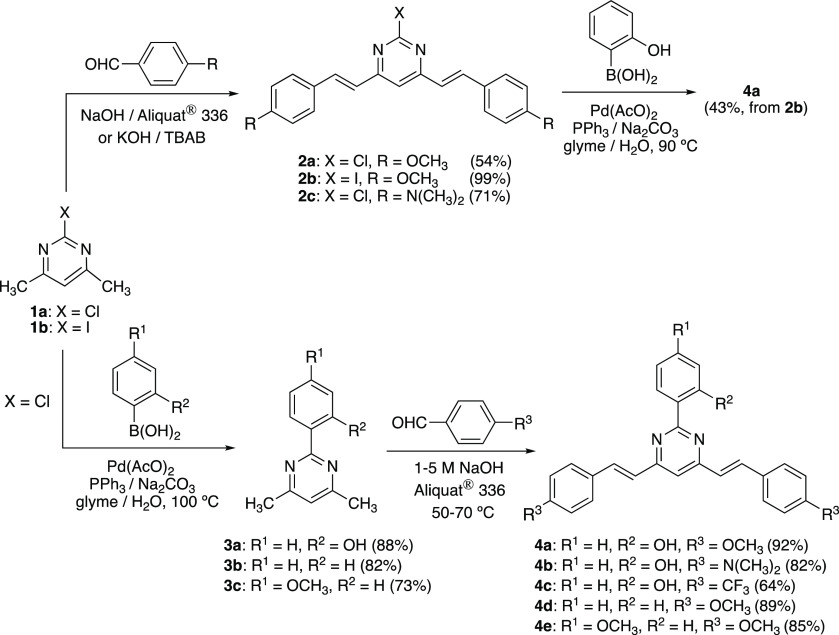
Synthesis of 2-Aryl-4,6-bis(arylvinyl)pyrimidines (**4a**–**4e**)

Two synthetic pathways can be envisaged for the synthesis of 2-aryl-4,6-bis(arylvinyl)pyrimidines, both based in a combination of Suzuki-Miyaura cross coupling and Knoevenagel condensation reactions. 2-Halo-4,6-dimethylpyrimidines **1** were used as starting materials to access 2-halo-4,6-bis(arylvinyl)pyrimidines **2** by condensation with the appropriate benzaldehyde in basic media. Chloro derivatives **2a** and **2c** were prepared in aqueous NaOH using Aliquat 336 as a phase-transfer catalyst.^45^ Microwave irradiation allowed us to reduce drastically the reaction time for **2a**. Meanwhile, the iodo derivative **2b** was obtained in excellent yield by solvent-free condensation.^46^ The subsequent coupling reaction of compound **2b** with 2-hydroxyphenylboronic acid under standard conditions afforded 2-(2′-hydroxyphenyl)-4,6-bis(4′-methoxystyryl)pyrimidine (**4a**) with moderate yield (Scheme 1, top). Nevertheless, this methodology proved unsuccessful when 2-chloro derivatives were used because of the lower reactivity of aryl chlorides.

The preparation was more straightforward, and the overall yield was substantially improved when the functionalization pattern of the starting materials was inverted (Scheme 1, bottom). Thus, starting from commercially available 2-chloro-4,6-dimethylpyrimidine (**1a**), 2-(2′-hydroxyphenyl)-4,6-dimethylpyrimidine (**3a**) was easily accessed. The synthesis of this compound had been previously reported from hydroxybenzamidine and acetylacetone with a very low yield of 12%.^47^ The final desired compounds **4a**–**4c** were obtained by Knoevenagel condensation with the corresponding aromatic aldehyde. In this case, the reaction was performed in hot aqueous NaOH. This protocol could also be used for the successful preparation of 2-aryl-4,6-bis(arylvinyl)pyrimidines **4d** and **4e**. Yields ranged from good to excellent. In all condensation reactions, the ^3^*J*(H–H) coupling constants of ∼16 Hz for the vinylic protons in the ^1^H NMR spectra indicated the selective formation of all-*E* isomers.

### 2.2 Synthesis of 2,4,6-Triarylpyrimidines

**Scheme 2:**
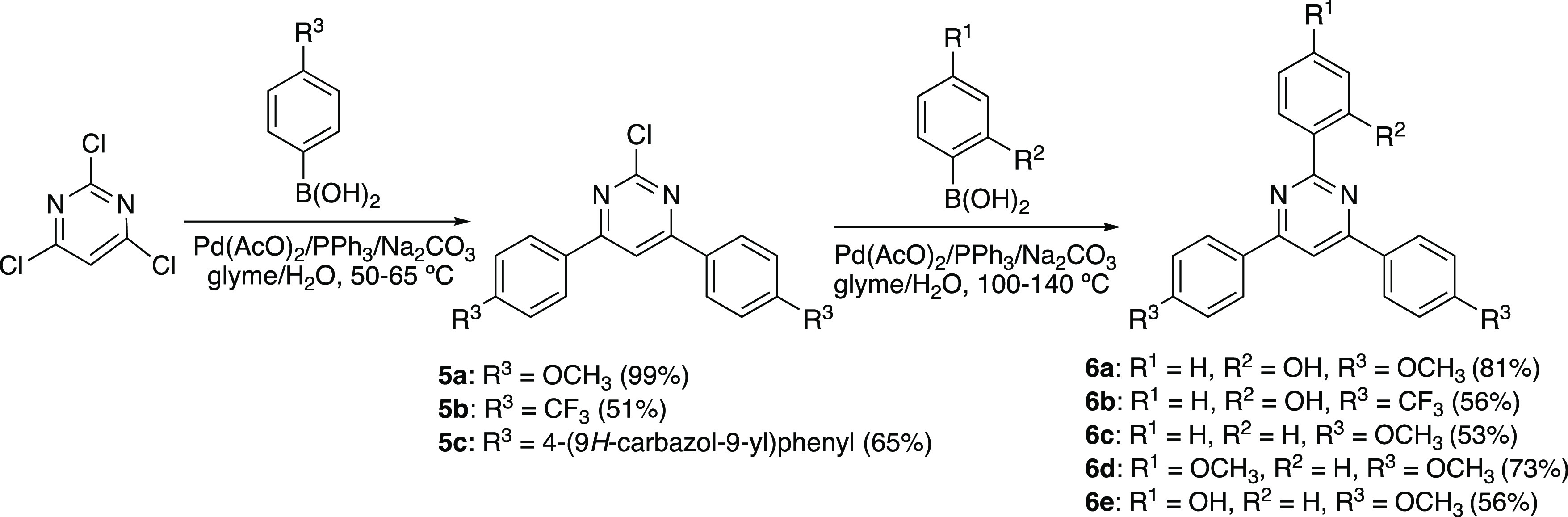
Synthesis of 2,4,6-Triarylpyrimidines (**6a**–**6e**)

Commercially available 2,4,6-trichloropyrimidine was chosen as starting material for the synthesis of 2,4,6-triarylpyrimidines because the higher reactivity of the C4 and C6 carbons over the C2 carbon allows one to perform Suzuki-Miyaura coupling reactions in a sequential manner.^48,49^ Thus, in a first reaction with 2 equiv of the appropriate boronic acid, we were able to obtain the 2-chloro-4,6-diarylpyrimidines **5** by selectively introducing two aryl groups under standard conditions at 50–65 °C. In a second step, a higher temperature (100 °C) was necessary to access the desired triarylsubstituted compounds **6** by reaction with a new molecule of boronic acid (Scheme 2). Under these conditions, no reaction was observed when **5c** was treated with 2-hydroxyphenylboronic acid, probably due to the higher electron-donor character of the carbazolyl group, which prevents the oxidative addition of Pd to the C–Cl bond. The temperature needed to be raised up to 140 °C when 4-hydroxyphenyl boronic acid was used, obtaining **6e** in moderate yield.

### 2.3 Photophysical Properties in Solution

The optical properties of compounds **4** and **6** were studied by UV/vis and fluorescence spectroscopy in CH_2_Cl_2_ solution at room temperature. The data obtained are summarized in Table 1. All compounds showed absorption maxima in the UV or visible region, which experienced a red shift on increasing the electron donor strength of the R^3^ group (**4b** > **4a** > **4c** and **6a** > **6b**). In most cases, a second or even a third absorption band of higher energy could be observed (Figure S1, Supporting Information).

**Table 1:** UV/Vis and Photoluminescence (PL) Data of Compounds **4** and **6**^a^

	CH_2_Cl_2_			CH_2_Cl_2_ + TFA^b^			solid (powder)	
compd	UV/vis λ_max_, nm (ε, mM^–1^·cm^–1^)	PL λ_max_, nm	Φ_F_^c^	UV/vis λ_max_, nm (ε, mM^–1^·cm^–1^)	PL λ_max_, nm	Φ_F_^d^	PL λ_max_, nm	Φ_F_^e^
**4a**	347 (41.3), 384 (45.4)			408 (33.8),^f^ 468 (55.4)	550	0.11		
**4b**	447 (55.2)			483 (6.9),^f^ 596 (27.4)	696	nd^g^		
**4c**	291 (39.9), 328 (34.2)^f^							
**4d**	320 (69.9),^f^ 371 (93.1), 384 (79.8)^f^	440	0.08				457	0.17
**4e**	278 (62.9), 329 (19.3), 373 (17.6), 390 (14.4)^f^	438	0.11				471	0.21
**6a**	277 (29.1),^f^ 299 (33.4), 330 (29.3)			359 (36.9), 395 (42.2)	436	0.34	444, 594	0.01
**6b**	270 (58.9), 327 (11.7)^f^							
**6c**	278 (31.8), 291 (31.5), 325 (19.1)	369	<0.01				388, 493	0.10
**6d**	285 (134.3), 333 (21.6)	374	0.02				385	0.03
**6e**	293 (37.3), 331 (12.4)	360,^f^ 371	0.01					

a All spectra were registered at room temperature (*c* = (0.38–5.80) × 10^–6^ M).

b Data after the addition of TFA (3000 equiv for **4a**, 1200 for **4b**, and 6000 for **6a**).

c Fluorescence quantum yield determined relative to those of 9,10-diphenylanthracene in cyclohexane (Φ_F_ = 0.90) and quinine sulfate in 0.1 M H_2_SO_4_ (Φ_F_ = 0.54) for **4d** and **4e** (λ_exc_ = 373 nm); 2-aminopyridine in 0.1 M H_2_SO_4_ (Φ_F_ = 0.60) for **6c** (λ_exc_ = 297 nm); anthracene in ethanol (Φ_F_ = 0.27) for **6d** and **6e** (λ_exc_ = 330 nm).

d Fluorescence quantum yield determined relative to those of fluorescein in 0.1 M NaOH (Φ_F_ = 0.82) for **4a** (λ_exc_ = 467 nm), and 9,10-diphenylanthracene in cyclohexane (Φ_F_ = 0.90) for **6a** (λ_exc_ = 390 nm).

e Fluorescence quantum yield calculated with a Jasco ILF-835/100 mm integrating sphere.

f Shoulder.

g Not determined (nd).

Compounds with R^2^ = OH (**4a**–**4c**, **6a**, and **6b**) were not fluorescent. Because they possess an intramolecular hydrogen bond, this absence of emission can be explained by a fast proton transfer reaction in the excited state from the OH group to the nitrogen atoms of the pyrimidine ring to yield an excited tautomer (see below). The tautomer can experience a charge-transfer process associated with a significant conformational change that leads to a radiationless decay.^50,51^ The key role of the OH group in the emission quenching was demonstrated by the preparation of derivatives with R^2^ = H (**4d**, **4e**, and **6c**–**6e**), all of which exhibited violet or blue luminescence (Table 1). Similar results were observed when the emission spectra were registered in the solid state. Whereas **4a**–**4c**, **6a**, and **6b** retained very little or no luminescence, compounds with R^2^ = H showed red-shifted emission maxima and higher quantum yields with respect to those obtained in solution, except **6e** (R^1^ = OH), which was not emissive (see Table 1 and Figures S2–S4).

The ESIPT process was thoroughly studied from a theoretical point of view by performing density functional theory (DFT) and TD-DFT calculations in CH_2_Cl_2_ solution and also in the crystal when available. Solvent effects were included using the polarizable continuum model (PCM) that accounts for implicit solvation (see the Supporting Information for computational details). Taking into account the similarity that exists both in the chemical structure and in the experimental photophysical properties, quantum-mechanical calculations were performed over compounds **4a**, **4d**, **4e**, **6a**, and **6c**–**6e**. An initial conformational analysis was performed at the M06-2*X*/6-31G** level of theory to determine the most stable conformation in solution (the relative energies are shown in Table S1). Figures S5 and S6 show some selected parameters of the optimized molecular geometry for the ground and excited states. It is worth commenting on the variations of the molecular geometry in compounds **4a** and **6a** after excitation. Our calculations predict O–H···N hydrogen bonds in the ground state as moderate, according to the Jeffrey criteria,^52^ which could predispose the molecule to the proton transfer in the excited state. For **4a**, a planar structure is predicted with dihedral angles along the molecular skeleton very close to zero for both S_0_ and S_1_ states. After excitation, the hydrogen-bond distance increases from 1.355 Å (S_0_, O–H···N) to 1.360 Å (S_1_, O···H–N), and the O–H–N bond angle decreases from 150° (S_0_) to 138° (S_1_), which would favor the stabilization of the keto form in the excited state. For compound **6a**, the hydroxyphenyl ring is twisted relative to the pyrimidine ring by ∼3°, and a significant deviation of the planarity is also found for the dihedral angles between the phenyl rings at the 4 and 6 positions and the central pyrimidine of ∼27° and −20° in the ground state. After excitation, these dihedral angles decrease up to 18° and 0.8°, while the ring at position 2 is twisted 19°. As in **4a**, the hydrogen-bond distance increases from 1.681 Å (S_0_, O–H···N) to 1.875 Å (S_1_, O···H–N), and the O–H–N bond angle decreases from 148° (S_0_) to 134° (S_1_). Thus, the O–H···N hydrogen bond weakens in **6a** after excitation, favoring the stabilization of the keto form in the excited state. Finally, compounds **4d** and **4e** are predicted to be almost planar in both ground and excited states. In contrast, compounds **6c**–**6e** present deviations in the ground state of 21° between the phenyl rings at the 4 and 6 positions and at the central pyrimidine and less than 0.2° between the pyrimidine and the phenyl ring at position 2. Nevertheless, these molecules become completely planar in the excited state.

**Figure 1:**
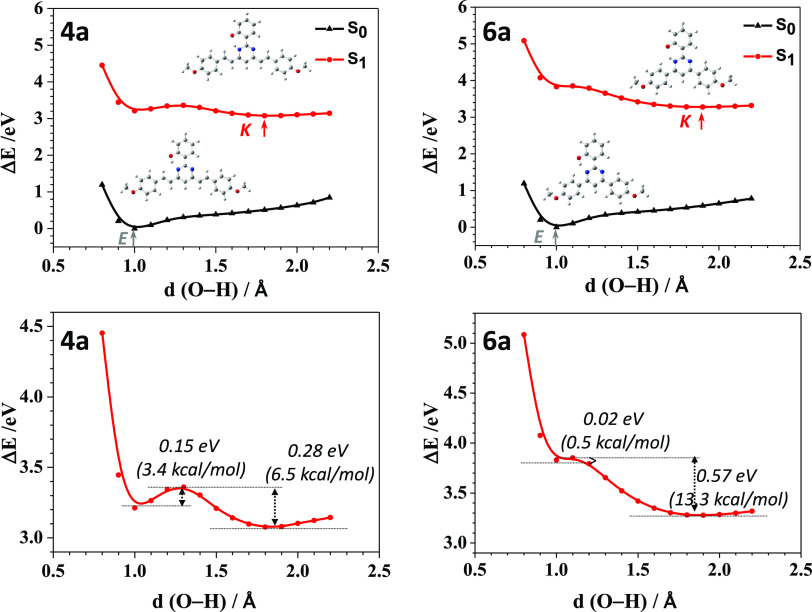
PES curves (top) computed for **4a** (left) and **6a** (right) in the Δ*E* scale at the M06-2*X*/6-31+G** level of theory in CH_2_Cl_2_ solution. The enol (E) and keto (K) forms are indicated at short and long O–H distances, respectively. An enlarged view of the energy barriers of S_1_ calculated for **4a** and **6a** in the excited state is shown in the bottom.

The relaxed potential energy scan (PES) from the enol form (E) to the keto form (K) were computed in CH_2_Cl_2_ solution for 2-(2′-hydroxyphenyl)pyrimidines **4a** and **6a**, enlarging the oxygen···hydrogen bond length of the OH group toward the nitrogen atom of the pyrimidine ring. As expected, the enol form is predicted to be more stable for both **4a** and **6a** in the ground state S_0_, whereas the keto form becomes more stable in the first excited state S_1_ (Figure 1, top; Table S1 lists the relative energies of the minima). As shown in Figure 1 (bottom), the height of the barrier for **4a** from the enol to keto form in S_1_ is 0.15 eV (3.4 kcal/mol), and the reversed barrier is 0.28 eV (6.5 kcal/mol). For **6a**, the energy barrier from the enol to keto form in S_1_ is 0.02 eV (0.5 kcal/mol), and the reversed barrier is 0.57 eV (13.3 kcal/mol). As a result, the low energy barriers from the enol to keto form and the high reversed-energy barriers would favor the ESIPT process in **4a** and **6a** and the fluorescence emission from the keto form.

**Figure 2:**
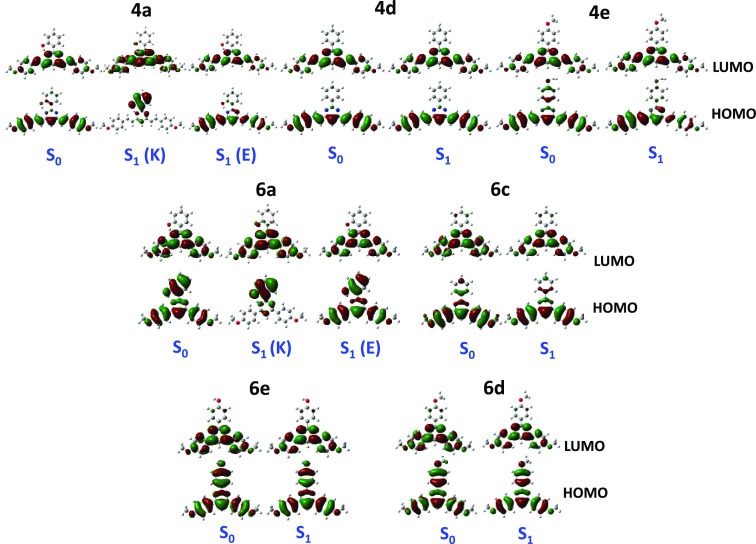
Molecular orbitals in CH_2_Cl_2_ solution calculated for the ground and excited states at the M06-2*X*/6-31+G** level of theory (isocontour plots 0.02 au).

Table 2 lists the vertical electronic transitions and oscillator strength (*f*) calculated at the M06-2*X*/6-31+G** level of theory in CH_2_Cl_2_ solution, considering both enol (E) and keto (K) tautomers (for more details see Table S2). There is a good agreement with the experimental absorption data, especially for compounds **4a**, **4d**, and **4e** with differences in the range of 0.1–0.3 eV. However, the differences observed were greater (0.3–0.5 eV) for compounds **6a** and **6c**–**6e**. For all compounds studied, the lowest energy transition S_0_ → S_1_ is predicted to be the strongest with a high contribution of the highest occupied molecular orbital (HOMO) → lowest unoccupied molecular orbital (LUMO) and therefore charge-transfer character. Figure 2 plots the HOMO and LUMO molecular orbitals. For compounds **4a**, **4d**, and **4e**, the HOMO is delocalized on the two styryl arms, while the LUMO is more localized on the pyrimidine ring. In **6a** and **6c**–**6e**, the HOMO is localized on the three phenyl rings, while the LUMO is localized on the pyrimidine ring.

**Table 2:** Maximum Absorption (λ_ab_^max^) and Emission Wavelengths (λ_em_^max^) Determined in CH_2_Cl_2_ Solution. Calculated Lowest-Energy Transition Wavelengths (λ_vert-ab_^calc^ and λ_vert-em_^calc^) and Oscillator Strengths (*f*) for These Transitions^a^

compd	λ_ab_^max^ eV (nm)	λ_vert-ab_^calc^ eV (nm)	*f*	% contribution	λ_em_^max^ eV (nm)	λ_vert-em_^calc^ eV (nm)	*f*
**4a**	3.23 (384)	3.45 (360)	1.86	H → L (90)		2.86 (434) E	2.06
						1.44 (859) K	0.09
**4d**	3.34 (371)	3.55 (350)	1.89	H → L (89)	2.82 (440)	2.88 (430)	1.54
**4e**	3.32 (373)	3.54 (350)	1.78	H → L (89)	2.83 (438)	2.90 (428)	1.94
**6a**	3.76 (330)	4.17 (297)	0.80	H→L (79)		2.83 (438) E	1.18
				H-1→L (12)		1.77 (702) K	0.12
**6c**	3.81 (325)	4.28 (290)	0.90	H → L (91)	3.36 (369)	3.84 (323)	0.86
**6d**	3.72 (333)	4.22 (294)	0.67	H → L (87)	3.32 (374)	3.76 (330)^b^	1.03
**6e**	3.75 (331)	4.23 (293)	0.70	H → L (89)	3.45 (360) 3.31 (371)	3.75 (331)	0.62

a Calculations were performed at the M06-2*X*/6-31+G** level of theory. The absorption corresponds to S_0_ → S_1_, and the emission corresponds to the S_1_ → S_0_ transition.

b Calculated with CAM-B3LYP.

A large red shift is predicted in the calculated S_1_ → S_0_ transition for the keto form compared to the enol form in compounds **4a** and **6a**. In addition, the predicted oscillator strength is significantly smaller in the keto form (*f* = 0.09 for **4a** and *f* = 0.12 for **6a**) compared to the enol form (*f* = 2.06 for **4a** and *f* = 1.18 for **6a**). Since the keto form is predicted to be more stable in the first excited state S_1_, the emission will occur from this form and could therefore account for the absence of emission in solution for these compounds in the experimental spectra.

**Figure 3:**
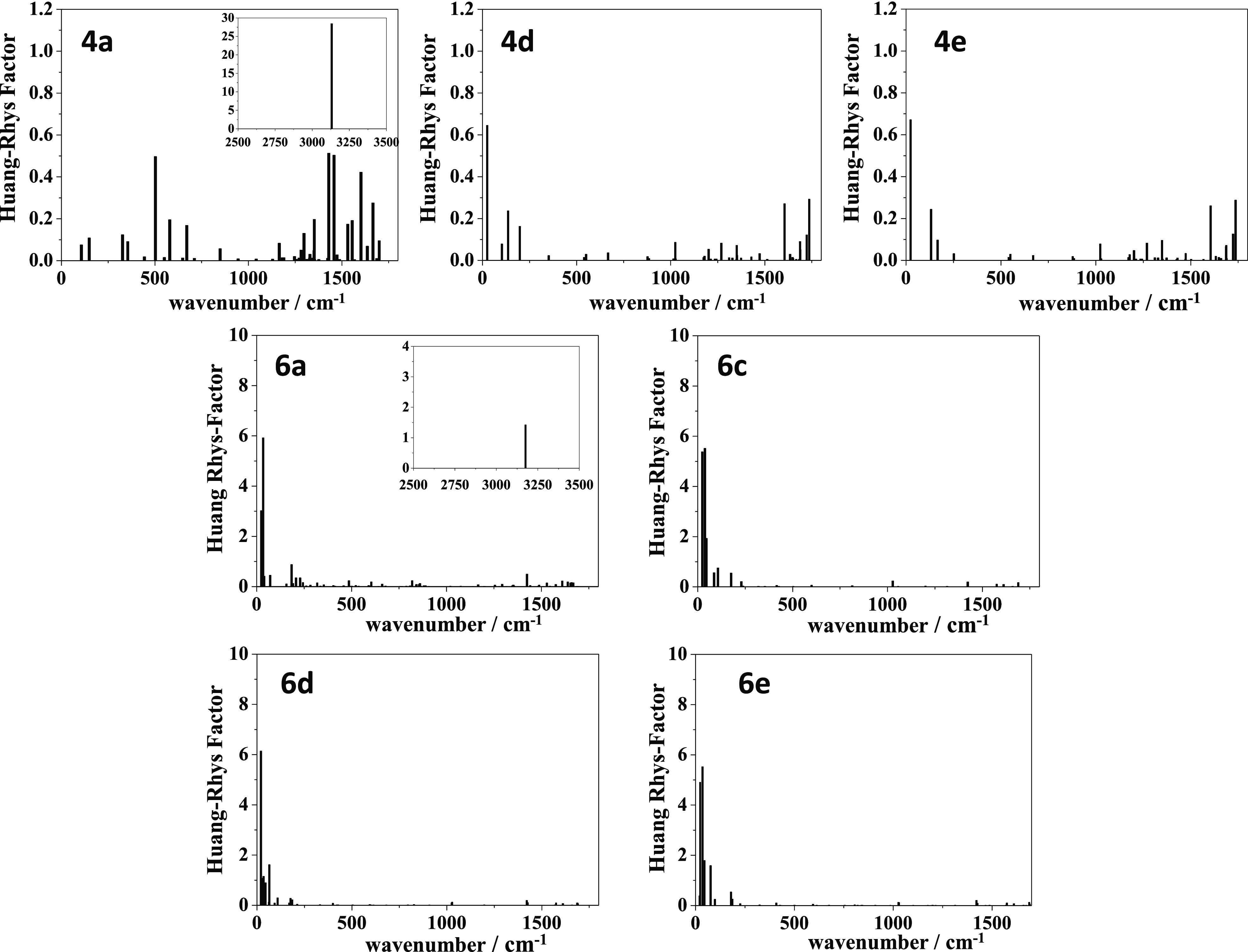
HR factors calculated for the ground state of compounds **4a**, **4d**, **4e**, **6a**, and **6c**–**6e** in CH_2_Cl_2_ at the M06-2*X*/6-31+G** level of theory.

The nonradiative vibrational relaxation from the excited state was also studied by calculating the Huang–Rhys (HR) factors in CH_2_Cl_2_ solution (see Table S3; the corresponding reorganization energies are shown in Figure S7).^53^Figure 3 shows that the largest values were calculated for compounds **4a** and **6a**, in agreement with the dark states observed by these compounds in solution. For **4a**, the largest HR factor is found for the vibrational mode associated with the OH stretching, calculated at 3131 cm^–1^ with an HR factor of 28. For **6a**, this vibrational mode is calculated at 3176 cm^–1^ with an HR factor of 1.4, and there are also two modes in the low-energy region predicted at 24 and 34 cm^–1^ with HR factors of 3 and 6, respectively. In view of our calculations, we postulate that the proton transfer in the excited state will be assisted mainly by the OH stretching mode in the high-energy region for **4a**, by the low-energy modes at 24 and 34 cm^–1^, and by the OH stretching mode for **6a**.

In contrast to the null emission observed for **4a**–**4c**, quantum yields of 8% and 11% were measured in solution for **4d** and **4e**, respectively (see Table 1). These results are consistent with the small vibrational relaxation predicted for these compounds, with 0.64 and 0.67 being the highest HR factors calculated for the vibrational modes 25 cm^–1^ (**4d**) and 24 cm^–1^ (**4e**), respectively. Furthermore, higher HR factors (∼6) were obtained for **6c**–**6e**, which could justify the lower quantum yields measured for these derivatives in solution (Φ_F_ ≤ 1%). Thus, our calculations predict two vibrational modes at 24 and 38 cm^–1^ for **6c**, at 21 cm^–1^ for **6d**, and at 23 and 35 cm^–1^ for **6e**, with HR factors around 5–6. Figures S8 and S9 show the atomic displacements for some of these vibrational modes.

### 2.4 Photophysical Properties in Solid State

Single crystals of **4a**, **4e**, and **6c**–**6e** suitable for X-ray diffraction analysis were obtained by vapor diffusion in CH_2_Cl_2_/CH_3_CN and CHCl_3_/MeOH solvent systems. This allowed us to investigate inter- and intramolecular interactions and molecular packing structures, which directly impact the emissive properties of the compounds in the solid state.^54^ Data processing and refinement parameters are given in Table 3.

**Table 3:** Crystallographic and Refinement Data for **4a**, **4e**, and **6c**–**6e**

compound	**4a**	**4e**	**6c**	**6d**	**6e**
CCDC number	2113241	2113242	2113243	2113244	2113245
formula	C_28_H_24_N_2_O_3_	C_29_H_26_N_2_O_3_	C_24_H_20_N_2_O_2_	C_25_H_22_N_2_O_3_	C_24_H_20_N_2_O_3_
FW (g·mol^–1^)	436.49	450.52	368.42	398.44	384.42
color, habit	white needle	yellow prism	white prismatic	white prismatic	white prismatic
crystal size (mm^3^)	0.030 × 0.035 × 0.300	0.20 × 0.15 × 0.06	0.27 × 0.23 × 0.07	0.190 × 0.185 × 0.035	0.228 × 0.150 × 0.123
crystal system	orthorhombic	monoclinic	monoclinic	monoclinic	monoclinic
space group	*Pca* 21	*P* 21/*c*	*P* 21/*n*	*P* 21	*P* 21/*n*
unit cell dimens. *a* (Å)	22.569(1)	15.917(1)	13.460(1)	6.483(1)	13.755(1)
*b* (Å)	5.140(1)	13.881(1)	7.515(1)	8.533(1)	7.797(1)
*c* (Å)	37.999(1)	10.594(1)	18.609(1)	18.029(1)	17.927(1)
α (deg)	90	90	90	90	90
β (deg)	90	98.99(1)	104.27(1)	95.01(1)	102.42(1)
volume (Å ^3^)	4408.2(2)	2312.1(1)	1824.2(1)	993.5(1)	1877.9(2)
Z	8	4	4	2	4
density (calc. Mg·m^–3^)	1.315	1.294	1.341	1.332	1.360
μ (mm^–1^)	0.086	0.084	0.086	0.088	0.091
F(000)	1840	952	776	420	808
θ range (deg)	2.099–27.177	1.96–27.12	2.14–7.51	2.27–27.19	2.33–28.76
index ranges	0 ≤ *h* ≤ 6	–20 ≤ *h* ≤ 20	–17 ≤ *h* ≤ 16	–8 ≤ *h* ≤ 8	–18 ≤ *h* ≤ 18
	0 ≤ *k* ≤ 28	–17 ≤ *k* ≤ 17	0 ≤ *k* ≤ 9	–10 ≤ *k* ≤ 10	–9 ≤ *k* ≤ 10
	–48 ≤ *l* ≤ 48	–13 ≤ *l* ≤ 13	0 ≤ *l* ≤ 24	0 ≤ *l* ≤ 23	–24 ≤ *l* ≤ 23
reflecs. collected	9721	18 962	4185	4409	46 689
indep./ *I* > 2σ(*I*)	9721	5113	4185	4409	4872
*R*_int_	0.086	0.0381	0.047	0.035	0.0429
weighting scheme *w*^–1^ = σ^2^(*F*_o_^2^) + (*xP*)^2^ + *yP* (*P* = (*F*_o_^2^ + 2*F*_c_^2^)/3)					
*x*/*y*	0.0624/0.8168	0.0513/0.3935	0.0379/1.1602	0.0419/0.1246	0.0573/1.0741
data/restraints/parameters	9721/1/636	5113/0/411	4185/0/333	4409/1/359	4872/0/342
goodness-of-fit on *F*^2^	1.036	1.037	1.077	1.061	1.061
R1/*w*R2 [*I* > 2σ(*I*)]	0.0619/0.1325	0.0391/0.0968	0.0474/0.1120	0.0347/0.0854	0.0409/0.1092
R1/*w*R2 (all data)	0.0850/0.1485	0.0539/0.1032	0.0544/0.1154	0.0403/0.0876	0.0516/0.1186
largest Δρ (e·Å^–3^)	0.844/–0.365	0.322/–0.205	0.957/–1.143	0.156/–0.239	0.390/–0.283

The compounds adopt monoclinic crystal systems, except **4a**, which prefers an orthorhombic structure with two crystallographically independent molecules in the asymmetric unit. Figure S10 shows the crystal structures for all compounds. In general, intramolecular C–H···N weak hydrogen bonds (2.48–2.83 Å) exist between the central pyrimidine and the adjacent phenyl rings, which could restrict the intramolecular distortion and increase the molecular stability. Crystal structures show that the compounds adopt a planar geometry: the dihedral angle between the plane of the rings and the molecular plane exhibits values between 5.1° and 18.1°, with the compound **6c** showing the greatest deviation (18.1°). For compounds **4a** and **6e**, the presence of the OH group in *ortho* (**4a**) and *para* (**6e**) position permits intra- and intermolecular hydrogen bonds, respectively. In the structure of **4a**, it can be observed an intramolecular interaction O2C–H2C···N3 (O2–N3 distance of 2.545(6) Å), whereas **6e** exhibits an intermolecular bond O4C–H4C···N1 (O4–N1 distance of 2.905(1) Å).

**Figure 4:**
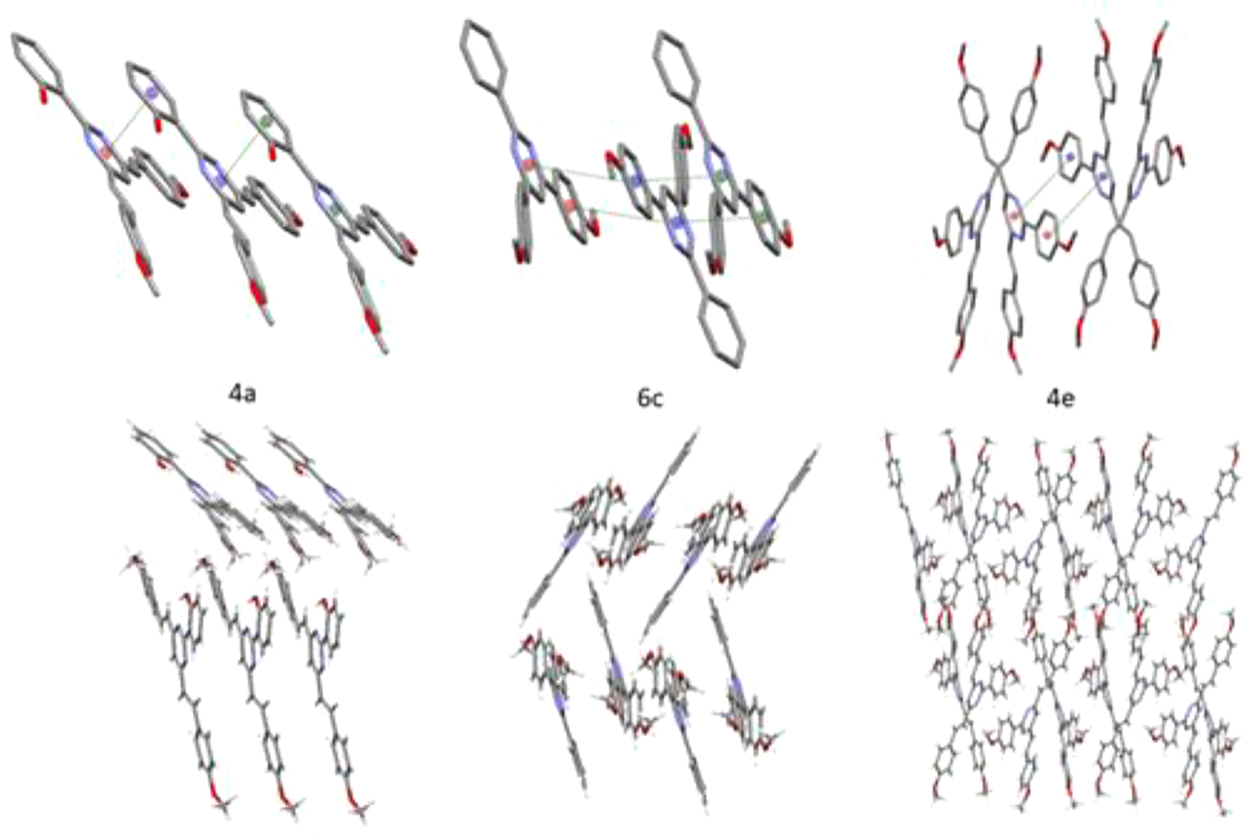
Pattern of the π–π interactions between molecules (top) and crystal packing (bottom) of compounds **4a**, **6c**, and **4e**. For clarity, neither hydrogen atoms (top) nor atom labels are displayed (crystal packing of **6d** and **6e** are shown in Figure S11).

The molecular packing patterns of the less-emissive compounds in solid **4a** and in **6c**–**6e** are similar. Crystal structures of **4a** and **6d** show the molecules stacked in parallel, while in **6c** and **6e** the arrangement is antiparallel. All the interactions were stablished by π–π interactions through the pyrimidine ring and one of the neighboring rings (Figure 4, top).^55^ Geometrical parameters defining the π–π interactions indicate the existence of a π-stacking arrangement with centroid distances that range between 3.5 and 4.0 Å. As it is known, these interactions quench the emission,^56^ which would agree with the low quantum yield measured for compounds **6c** and **6d** in the solid state (10% and 3%, respectively) and the null emission for **4a** and **6e**. Meanwhile, multiple weak interactions such as C–H···π interactions and no classical hydrogen bonds promote three-dimensional structures in which the stacking of molecules shows an angular disposition (Figure 4, bottom), with angular dihedral angles between 40.73° in **4a** and 73.10° (quasi parallel disposition) in **6d**. In contrast, the three-dimensional structure of **4e** shows isolated molecules and exhibits only an intramolecular weak bond (C42–H42···N3; 2.841(2) Å). Moreover, the intermolecular interactions are weaker than in the other structures; for instance, the distances between planes to form the π–π stacking are larger (ca. 4.54 Å). These weaker interactions could lead to improved emission, thus increasing the photoluminescence quantum yield in the solid state for **4e** up to 21%.

**Figure 5:**
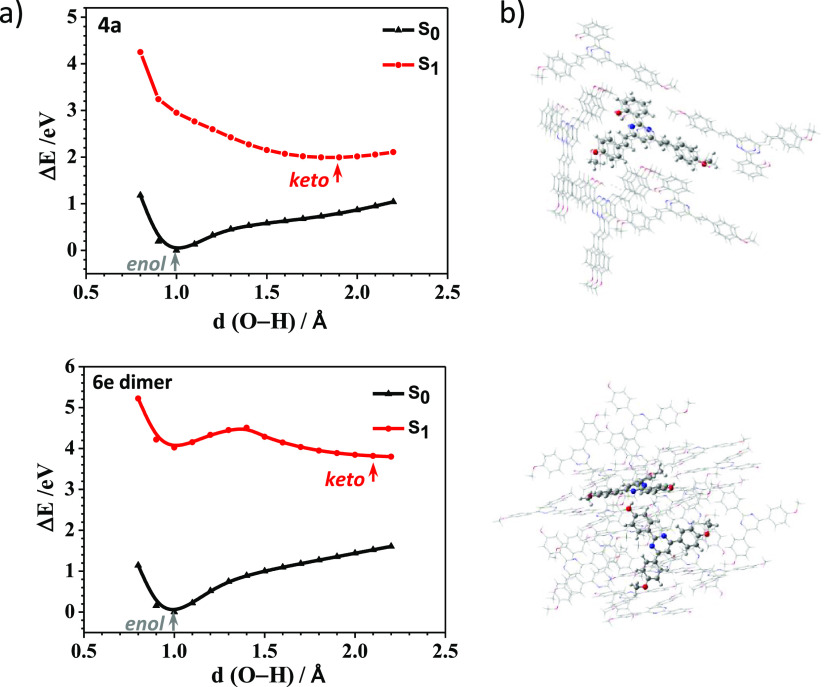
(a) PES curves computed for the central molecule of **4a** (top) and the dimer of **6e** (bottom) in the Δ*E* scale at the M06-2*X*/6-31G** level of theory. The enol (E) and keto (K) forms are indicated at short and long O–H distances, respectively. (b) Molecular clusters computed at the QM/MM level. The central molecule is treated as high level (M06-2*X*/6-31G**) and the surrounding molecules as low level (UFF41).

The possibility of ESIPT in the crystal was also investigated by performing TD-DFT calculations for compound **4a**. For **6e**, we analyzed the possibility of an intermolecular proton transfer in the excited state in a dimer. In both cases, we built a cluster of 15 molecules from the crystal structure. The relaxed PES from the enol form (E) to the keto form (K) were computed by enlarging the oxygen···hydrogen bond length of the OH group to the nitrogen atom of the pyrimidine ring in compound **4a** and to the neighboring molecule in dimer **6e**, leaving the surrounding molecules frozen. As expected, the enol form is more stable for both **4a** and **6e** in the ground state S_0_ (Figure 5). For compound **4a**, the energy barrier for the ESIPT process disappears, and the keto form becomes more stable in the first excited state S_1_. However, the energy barrier for the intermolecular proton transfer in **6e** is 0.49 eV (11.1 kcal/mol), and the ESIPT process in the solid state would be less favored than in solution (Figure S12). In any case, this result should be taken with caution due to the simplicity of our model, which considers only the excitation of one dimer in the crystal. Table S4 lists the calculated emission for the enol (E) and keto (K) forms of the central molecule of **4a** and the dimer **6e**. The calculated oscillator strength for the keto form of **4a** is almost zero (*f* = 0.008), which is in agreement with the lack of emission of this compound in the solid state. Although the enol form is more stable than the keto form for the dimer of **6e**, the oscillator strength is also very small (*f* = 0.4), which could account for the absence of emission in the solid state.

### 2.5 Effect of Protonation: Experimental Results and Computational Insights

**Figure 6:**
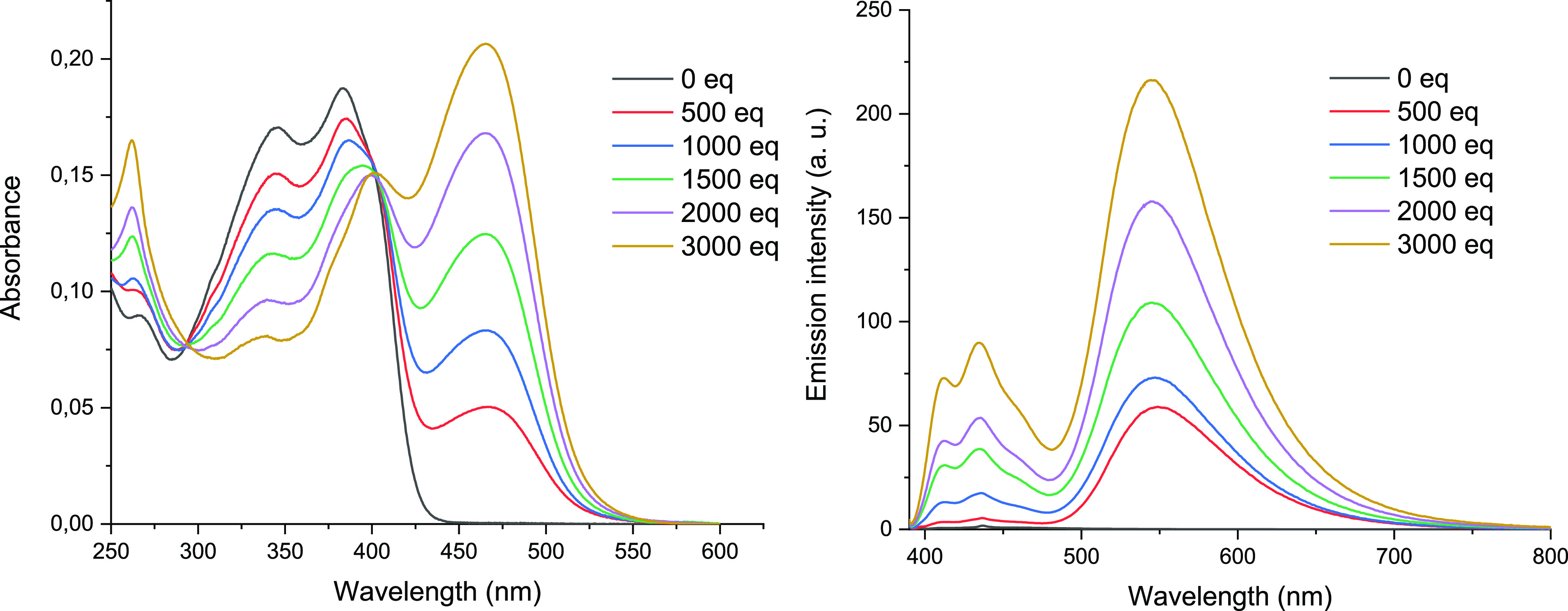
Changes in the absorption (left) and emission (right, λ_exc_ = 384 nm) spectra of a CH_2_Cl_2_ solution of **4a** (*c* = 4.12 × 10^–6^ M) upon addition of TFA.

The effect of protonation of the pyrimidine ring on the photophysical properties was also studied by titration of CH_2_Cl_2_ solutions with trifluoroacetic acid (TFA). The changes observed in the absorption and emission spectra for compound **4a** are illustrated in Figure 6. The UV/vis spectra showed the progressive attenuation of the absorption band for the neutral compound on increasing the concentration of acid, whereas a new red-shifted absorption band progressively appeared. The spectra showed an isosbestic point at 402 nm, and this is characteristic of an equilibrium between two species (Figure 6, left). The bathochromic shift is explained by the higher degree of intramolecular charge transfer (ICT) due to an increase in the electron-withdrawing character of the pyrimidine ring, as observed previously.^32^ More than 3000 equiv of TFA was required for a complete protonation.

**Figure 7:**
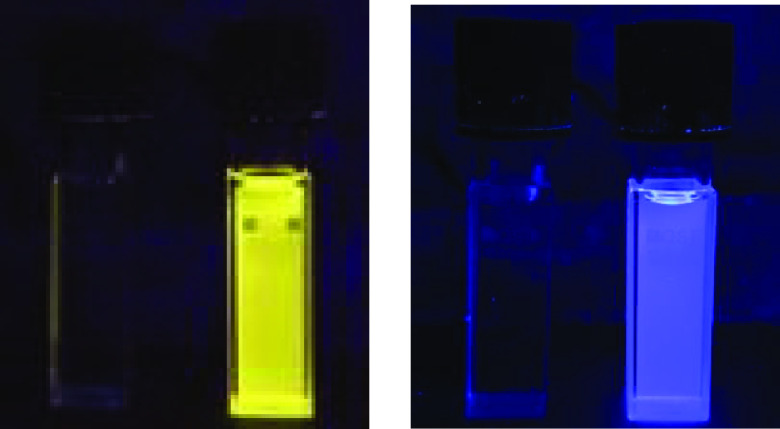
Change in the color of a CH_2_Cl_2_ solution of **4a** (left) and **6a** (right) after the addition of TFA (3000 equiv for **4a** and 1500 equiv for **6a**). The pictures were taken in the dark upon irradiation with a UV hand-held lamp (λ_exc_ = 365 nm, 24 W).

As mentioned above, the neutral solution of **4a** did not exhibit luminescence. Nevertheless, the addition of acid resulted in the progressive appearance of a green-yellow emission band (λ_max_ = 550 nm) whose intensity increased with the concentration of acid (Figure 6, right). The addition of acid should inhibit the ESIPT process, effectively interrupting the nonradiative deactivation pathway of the excited state. This fact accounts for the observed *switch on* fluorescence response, which was also readily detectable by the naked eye (Figure 7, left).

A similar behavior was observed for compounds **4b** and **6a** (Figure 7 right and Figures S13 and S14), although the protonated form of **4b** showed a poor fluorescence signal because of a greater ICT. In contrast, the optical properties of **4c** and **6b** remained almost unaltered in the presence of an excess of TFA, which denoted an ineffective protonation. The strong electron-withdrawing character of the CF_3_ groups should decrease the basicity of the pyrimidine nitrogen atoms.^57,58^ Titrations of acetonitrile solutions of **4a** and **6a** with aqueous HCl gave similar results to those obtained with TFA, although less acid was required for complete protonation due to the higher acidity of HCl. All these data seem to rule out the formation of a dication at least at the concentration of acid used.

**Figure 8:**
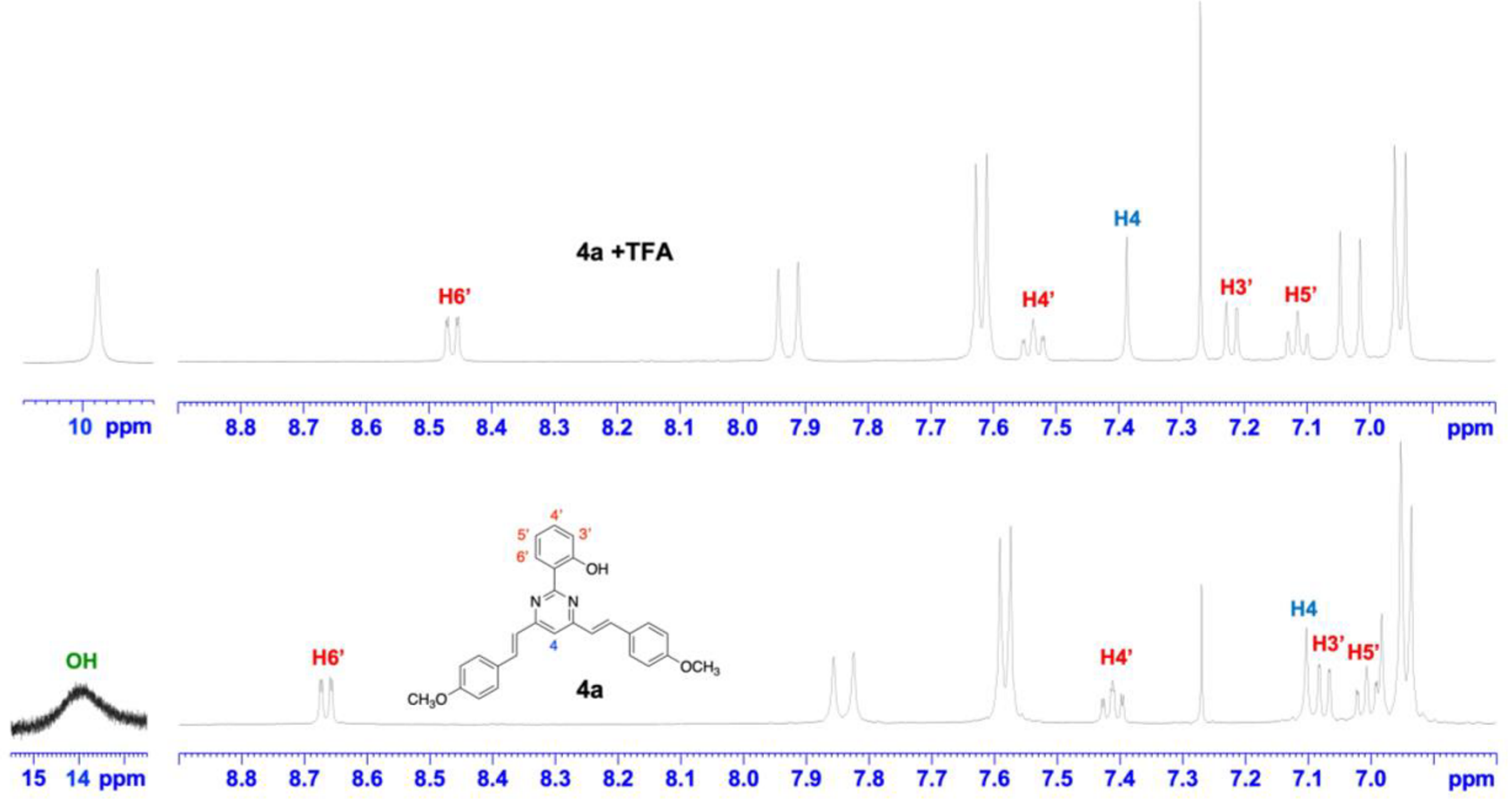
Expanded regions of the ^1^H NMR spectrum of **4a** before (bottom) and after (top) the addition of an excess of TFA (CDCl_3_, 500 MHz).

**Figure 9:**
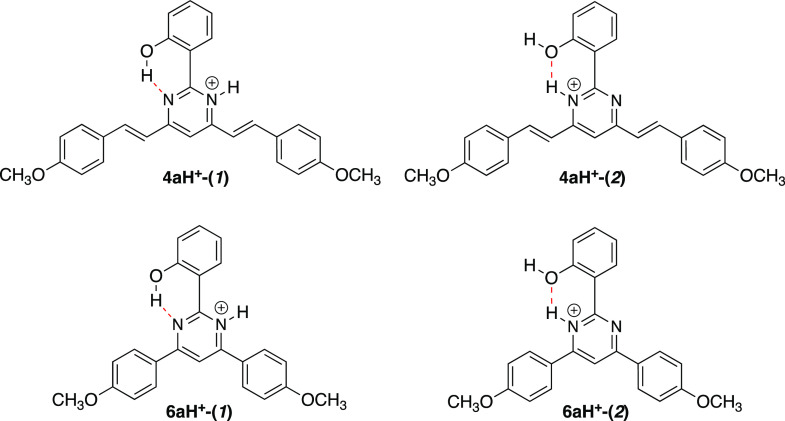
Possibilities of protonation for compounds **4a** and **6a**.

Protonation of **4a** and **6a** was also studied by ^1^H NMR spectroscopy. Upon addition of an excess of TFA to a CDCl_3_ solution of **4a**, most of the signals were shifted downfield, except proton H6′ of the 2′-hydroxyphenyl group, which experienced an upfield shift (Figure 8). A similar behavior was observed for the protonation of **6a** (Figure S15). The addition of acid should lead to an equilibrium between the two possible monoprotonated species (Figure 9). This equilibrium is fast on the NMR time scale, and the only set of signals observed is an average of both species. If only one of these species was formed, each branch at positions 4 and 6 of the pyrimidine ring would give a different set of signals.

TD-DFT calculations were also performed on protonated compounds **4a** and **6a** in order to investigate the origin of the emission after protonation. The molecular geometry for the ground and excited states were optimized at the M06-2*X*/6-31+G** level of theory in CH_2_Cl_2_ solution exploring the two possibilities of protonation as shown in Figure 9 (see geometrical parameters in Figure S16). Protonation leading to **4aH**^**+**^**-(2)** and **6aH**^**+**^**-(2)** provides further stabilization and lack of ESIPT. These protonated species are slightly more stable (Δ*E* ≈ 1 kcal/mol) than **4aH**^**+**^**-(1)** and **6aH**^**+**^**-(1)** (relative energies are listed in Table S5).

Table 4 lists the vertical electronic transitions along with the experimental absorption and emission data (see also Table S6 for more transitions). The theoretical predictions are in good agreement with the experimental observations, with deviations in the range of 0.1–0.4 eV for **4aH**^**+**^**-(1)** and **6aH**^**+**^**-(1)** and up to 0.5 eV for **4aH**^**+**^**-(2)** and **6aH**^**+**^**-(2)**. The lowest-energy S_0_ → S_1_ transition is predicted to be the strongest with a high contribution of the HOMO → LUMO transition and therefore charge-transfer character. Figure S17 plots the HOMO and LUMO molecular orbitals for the protonated compounds.

**Table 4:** Maximum Absorption (λ_ab_^max^) and Emission Wavelengths (λ_em_^max^) Determined for Protonated Compounds **4a** and **6a**. Calculated Lowest-Energy Transition Wavelengths (λ_vert-abs_^calc^ and λ_vert-em_^calc^) and Oscillator Strengths (*f*) for These Transitions^a^

compd	λ_ab_^max^ eV (nm)	λ_vert-ab_^calc^ eV (nm)	*f*	% contr.	λ_em_^max^ eV (nm)	λ_vert-em_^calc^ eV (nm)	*f*
**4aH**^**+**^**-(1)**	2.65 (468)	2.76 (450)	2.06	H → L (92)	2.25 (550)	2.61 (476) E	2.39
						0.82 (1516) K	0.09
**4aH**^**+**^**-(2)**		2.92 (425)	2.04	H → L (91)		2.72 (456)	2.37
**6aH**^**+**^**-(1)**	3.14 (395)	3.46 (359)	1.09	H → L (90)	2.84 (436)	3.14 (396) E	1.42
						1.20 (1036) K	0.05
**6aH**^**+**^**-(2)**		3.62 (342)	1.12	H → L (89)		3.33 (372)	1.47

a Calculations were performed at the M06-2*X*/6-31+G** level of theory in CH_2_Cl_2_ solution. The absorption corresponds to S_0_ → S_1_, and the emission corresponds to S_1_ → S_0_ transition.

**Figure 10:**
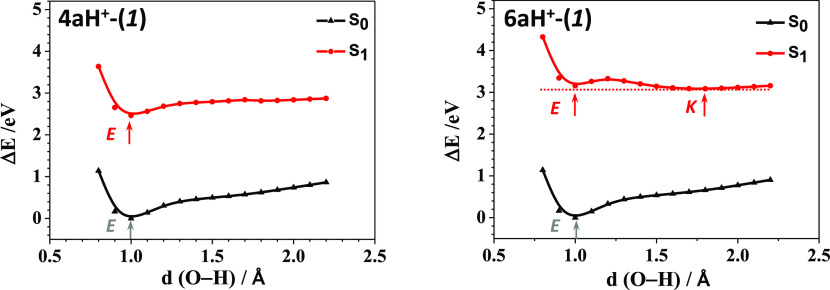
PES curves computed for protonated **4aH**^**+**^**-(1)** and **6aH**^**+**^**-(1)** in the Δ*E* scale at the M06-2*X*/6-31+G** level of theory in CH_2_Cl_2_ solution. The enol (E) and keto (K) forms are indicated at short and long O–H distances, respectively.

The relaxed PES from the enol form (E) to the keto form (K) were also computed for **4aH**^**+**^**-(1)** and **6aH**^**+**^**-(1)**, enlarging the oxygen···hydrogen bond length of the OH group to the nitrogen atoms of the pyrimidine ring. As shown in Figure 10, the enol form is more stable for both protonated species in the ground state S_0_. The calculated energy barrier predicts the stabilization of the enol form of **4aH**^**+**^**-(1)** in the first excited state S_1_; that is, the ESIPT would not occur in **4aH**^**+**^**-(1)** (Table S5 lists the relative energies of the minima). As shown in Table 4, a significant oscillator strength (*f* = 2.39) is predicted not only for the S_1_ → S_0_ electronic transition of the enol form of **4aH**^**+**^**-(1)** but also for **4aH**^**+**^**-(2)** (*f* = 2.37), which could account for the observed emission after the addition of acid (Φ_F_ = 11%). Namely, according to our calculations, both protonation positions would result in emissive species in the case of compound **4a**.

With regard to **6aH**^**+**^**-(1)**, the keto form in S_1_ is 1.6 kcal/mol more stable than the enol form (Figure S18 shows an enlarged view of the calculated energy barrier in the excited state S_1_). The energy barrier from the enol to keto form in S_1_ is 0.17 eV (3.9 kcal/mol), and the reversed barrier is 0.24 eV (5.5 kcal/mol). These results do not justify the emission increase observed for compound **6a** after protonation (Φ_F_ = 34%). Nevertheless, the calculated oscillator strength for the S_1_ → S_0_ transition of **6aH**^**+**^**-(2)** (*f* = 1.47) does explain the observed emission.

**Figure 11:**
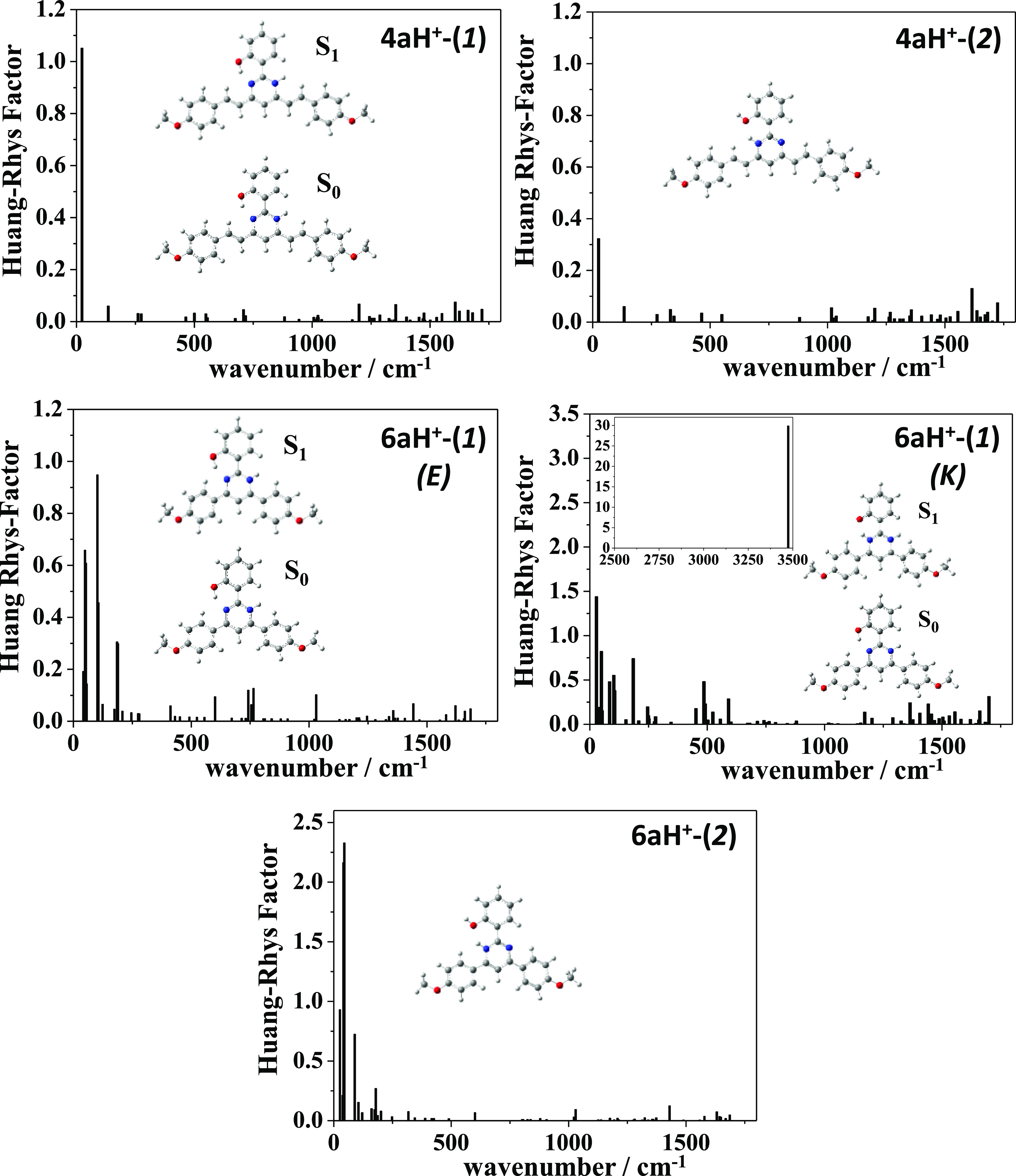
HR factors for the ground state of protonated **4a** and **6a** calculated at the M06-2*X*/6-31+G** level of theory in CH_2_Cl_2_.

Furthermore, small HR factors were calculated for protonated **4a** and **6a**, which supported the *switch on* fluorescence response observed for these compounds after protonation (Figure 11, Table S7). The largest HR factor for **4aH**^**+**^**-(1)** was ∼1 for the vibrational mode at 23 cm^–1^ in the low-frequency region. The calculated values for **4aH**^**+**^**-(2)** were even smaller than those for **4aH**^**+**^**-(1)**. Therefore, both protonation positions would favor the radiative relaxation. In **6aH**^**+**^**-(1)**, if the relaxation occurs from the enol form, there are several modes in the low-energy region with HR < 1, which could favor the radiative relaxation. In contrast, if the molecule relaxes from the keto form (predicted more stable than the enol form), a large value of HR = 30 is calculated in the high-frequency region for the mode at 3474 cm^–1^ in detriment of the emission. For **6aH**^**+**^**-(2)**, small HR factors (∼2) were obtained in the low-energy region, thus clearly favoring the radiative relaxation.

### 2.6 Hydrogen-Bonding Strength

The potential energy barrier of the C–C–C–N dihedral angle between the pyrimidine and the phenyl ring at position 2 was calculated in the ground state in order to estimate the ease of rotation of the phenyl ring. As expected, the highest rotational energy barrier was obtained for compounds **4a** and **6a** (∼11 kcal/mol), which is indicative of the strength of the hydrogen bond (Figure S19). The phenyl ring may undergo twisting motions more easily for the rest of the compounds. With regard to the protonated species, the height of the barrier correlates well with the strength of the hydrogen bonds; that is, **4a** > **4aH**^**+**^**-(1)** > **4aH**^**+**^**-(2)**, and **6a** > **6aH**^**+**^**-(1)** > **6aH**^**+**^**-(2)** (see below).

**Figure 12:**
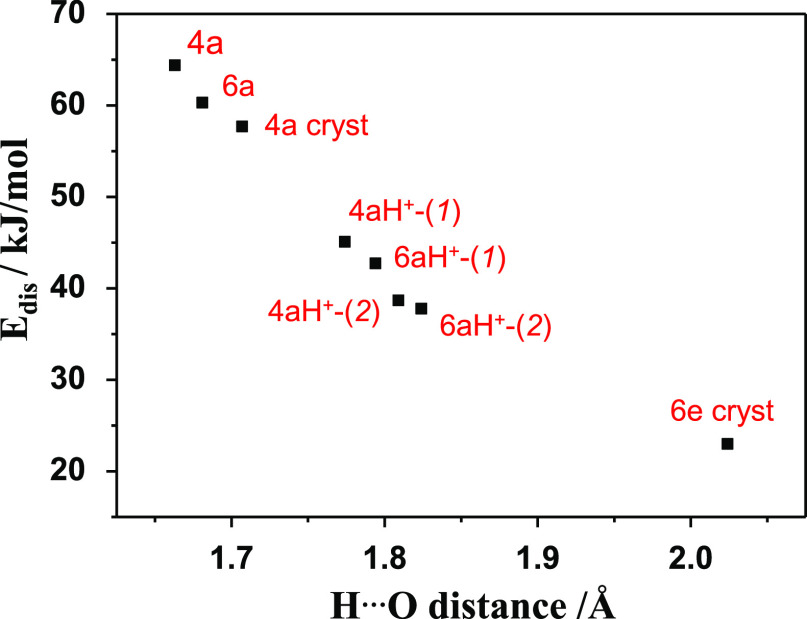
Dependence of the dissociation energy (*E*_dis_) vs H···O distance.

Additionally, to confirm the relative strength of the intramolecular hydrogen bonds predicted in compounds **4a** and **6a**, before and after protonation, we performed Quantum Theory of Atoms In Molecules (QTAIM) calculations in the context of Bader’s theory using the ground-state optimized molecular geometry of **4a** and **6a** in CH_2_Cl_2_ solution as well as the molecular geometry derived from the crystal structure of **4a** and **6e**.^59,60^Figure S20 shows the distribution of critical points (CPs) and bond paths for inter- and intramolecular hydrogen bonds. Table S8 lists the calculated QTAIM parameters of the hydrogen bonds. The dissociation energy (*E*_dis_) was calculated to evaluate the strength of the hydrogen bonds by using the methodology proposed by Espinosa et al.^61^Figure 12 plots the *E*_dis_ versus the hydrogen bond (HB) distance. The QTAIM analysis revealed that *E*_dis_ for compounds **4a** and **6a** are higher and that, consequently, the intramolecular HBs are stronger than those of the corresponding protonated species. These results indicate that the ESIPT process is more favored before protonation. *E*_dis_ is also higher for **4aH**^**+**^**-(1)** (45.1 kJ/mol) and **6aH**^**+**^**-(1)** (42.7 kJ/mol) compared to **4aH**^**+**^**-(2)** (38.7 kJ/mol) and **6aH**^**+**^**-(2)** (37.8 kJ/mol). In fact, the keto form of **6aH**^**+**^**-(1)** becomes more stable than the enol form in the excited state, thus favoring the ESIPT process and the absence of emission. Although the hydrogen bonds for **4aH**^**+**^**-(2)** and **6aH**^**+**^**-(2)** are weaker than for **4aH**^**+**^**-(1)** and **6aH**^**+**^**-(1)**, these intramolecular interactions cause a rigidification in that part of the molecule (see Figure S16), preventing the ESIPT process and increasing the fluorescence emission. Regarding the crystal data, the weakest HB corresponds to the intermolecular HB in the dimer of **6e** (*E*_dis_ = 23.0 kJ/mol), while the intramolecular HB for compound **4a** (*E*_dis_ = 57.7 kJ/mol) is close to that in solution (*E*_dis_ = 64.4 kJ/mol), which again favors the ESIPT process and the loss of emission in the solid state.

### 2.7 Anticounterfeiting Applications

**Figure 13:**
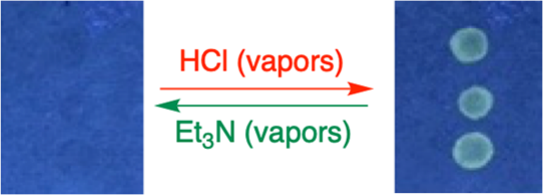
Digital photographs of the reversible color change of Whatman filter paper under UV light (365 nm) using compound **4a** as an anticounterfeiting agent.

The illicit trafficking of counterfeit goods is one of the largest money-making sources for organized crime and is a global serious concern.^62^ Fluorescent security inks are among the most widely used techniques to prevent counterfeiting. A higher level of security can be achieved simply by implementing a passive invisible ink as a tag.^63,64^ On the basis of the remarkable luminescence response aroused by acid, the fluorescence probes **4a** and **6a** look to be appropriate for anticounterfeiting applications. Thus, three drops of a solution of **4a** (4.12 × 10^–5^ M in CH_2_Cl_2_) were deposited on a piece of Whatman filter paper, which were invisible both under daylight and under UV light. Upon exposure to acid vapors (HCl), the droplets were prominent as greenish-yellow circles under UV light (Figure 13) with a response time of a few seconds (1–5 s). The paper was further exposed to triethylamine vapor to check for reversibility. The emission could be *switched on* and *off* for at least 10 cycles allowing the material to be used multiple times. The fluorescent color on the paper also slowly faded over time. A similar phenomenon was observed for **6a** (Figure S21).

## 3 Experimental Section

All experimental methods and materials as well as the synthesis and characterization of all compounds are fully described in detail in the Supporting Information. Here we include a few representative examples.

### 3.1 2-(2′-Hydroxyphenyl)-4,6-dimethylpyrimidine (**3a**)^65^

2-Chloro-4,6-dimethylpyrimidine (1000 mg, 7.02 mmol), 2-hydroxyphenylboronic acid (1065 mg, 7.72 mmol), and sodium carbonate (3720 mg, 35.1 mmol, dissolved in a minimum amount of water) were mixed with 1,2-dimethoxyethane (8 mL). Palladium acetate (79 mg, 0.35 mmol) and triphenylphosphine (183 mg, 0.70 mmol) were then added. The mixture was bubbled with argon for 5 min and heated at 100 °C in a flask sealed with a screw cap for 48 h. The solvent was evaporated, water was added, and the mixture extracted with dichloromethane (three times). The combined organic extracts were dried (MgSO_4_), and the solvent was evaporated. The crude product was purified by column chromatography (alumina, hexanes/ethyl acetate (EtOAc) mixtures, 10:0 to 9:1) to give a colorless solid (1230 mg, 88%). Further purification was achieved by crystallization from hexanes. ^1^H NMR (CDCl_3_, 500 MHz) δ: 2.56 (s, 6H, 2 × CH_3_), 6.94 (s, 1H, pyr), 6.94–6.97 (m, 1H, ArH), 7.02 (dd, 1H, *J* = 8.0 Hz, *J* = 1.0 Hz, ArH), 7.38 (m, 1H, ArH), 8.54 (dd, 1H, *J* = 8.0 Hz, *J* = 1.5 Hz, ArH). ^13^C NMR and distortionless enhancement by polarization transfer (DEPT) (CDCl_3_, 125 MHz) δ: 165.9 (C), 164.2 (C), 160.7 (C), 133.0 (CH), 129.2 (CH), 118.9 (CH), 118.6 (C), 117.8 (CH), 117.5 (CH), 23.9 (CH_3_). IR (ATR) ν: 1561, 1434, 1366, 1250, 848, 759 cm^–1^.

### 3.2 (E,E)-2-(2′-Hydroxyphenyl)-4,6-bis(4′-methoxystyryl)pyrimidine (**4a**)

A stirred mixture of 2-(2′-hydroxyphenyl)-4,6-dimethylpyrimidine (100 mg, 0.5 mmol), 4-methoxybenzaldehyde (136 mg, 1 mmol), Aliquat 336 (22 mg, 0.05 mmol), and 5 M NaOH (8 mL) was heated at 50 °C for 22 h. After this mixture was cooled, the precipitated yellow solid was collected by filtration and purified by washing with boiling methanol (200 mg, 92%). mp: 175–177 °C (EtOAc/MeOH). ^1^H NMR (CDCl_3_, 500 MHz) δ: 3.86 (s, 6H, 2 × OCH_3_), 6.94 (A of AB_q_, 4H, *J* = 8.5 Hz, ArH), 6.97 (A of AB_q_, 2H, *J* = 16.0 Hz, 2 × CH=), 6.99–7.02 (m, 1H, ArH), 7.07 (dd, 1H, *J* = 8.0 Hz, ArH), 7.10 (s, 1H, pyr), 7.39–7.43 (m, 1H, ArH), 7.58 (B of AB_q_, 4H, *J* = 8.5 Hz, ArH), 7.84 (B of AB_q_, 2H, *J* = 16.0 Hz, 2 × CH=), 8.67 (dd, 1H, *J* = 8.0 Hz, *J* = 1.5 Hz, ArH), 13.95 (br s, 1H, OH). ^13^C NMR and DEPT (CDCl_3_, 125 MHz) δ: 164.1 (C), 161.9 (C), 161.0 (C), 160.8 (C), 137.5 (CH), 133.0 (CH), 129.4 (CH), 128.2 (C), 122.8 (CH), 119.0 (C), 118.9 (CH), 117.7 (CH), 114.4 (CH), 113.1 (CH), 55.4 (CH_3_). Matrix-assisted laser desorption/ionization time-of-flight mass spectrometry (MALDI-TOF MS) (2,5-dihydroxybenzoic acid (DHB)) *m*/*z*: 437.3 [M + H]^+^. IR (attenuated total reflectance (ATR)) ν: 1526, 1593, 1560, 1508, 1367, 1242, 1161, 1022, 972, 840, 754 cm^–1^. Anal. Calcd for C_28_H_24_N_2_O_3_: C, 77.04; H, 5.54; N, 6.42. Found: C, 76.86; H, 5.39, N, 6.62%.

### 3.3 2-Chloro-4,6-bis(4′-methoxyphenyl)pyrimidine (**5a**).^66,67^

2,4,6-Trichloropyrimidine (500 mg, 2.73 mmol), 4-methoxyphenylboronic acid (830 mg, 5.46 mmol), and sodium carbonate (1810 mg, 17.1 mmol, dissolved in a minimum amount of water) were mixed with 1,2-dimethoxyethane (10 mL). Palladium acetate (15 mg, 0.068 mmol) and triphenylphosphine (36 mg, 0.137 mmol) were then added. The mixture was bubbled with argon for 10 min and heated at 50 °C in a flask sealed with a screw cap for 24 h. The solvent was evaporated, water was added, and the mixture was extracted with dichloromethane (three times). The combined organic extracts were dried (MgSO_4_), concentrated under vacuum, and filtered through a short pad of diatomaceous earth and alumina. Finally, the solvent was evaporated, and the crude product was washed with boiling methanol to give a colorless solid (880 mg, 99%). Further purification was achieved by crystallization from EtOAc/hexanes. ^1^H NMR (CDCl_3_, 500 MHz) δ: 3.91 (s, 6H, 2 × CH_3_), 7.03 (A of AB_q_, 4H, *J* = 9.0 Hz, ArH), 7.88 (s, 1H, pyr), 8.13 (B of AB_q_, 4H, *J* = 9.0 Hz, ArH). ^13^C NMR and DEPT (CDCl_3_, 125 MHz) δ: 166.7 (C), 162.5 (C), 161.8 (C), 129.0 (CH), 128.2 (C), 114.4 (CH), 109.0 (CH), 55.5 (CH_3_).

### 3.4 2-(2′-Hydroxyphenyl)-4,6-bis(4′-methoxyphenyl)pyrimidine (**6a**)

This compound was prepared from the pyrimidine derivative **5a** (640 mg, 2.02 mmol) and 2-hydroxyphenylboronic acid (306 mg, 2.22 mmol) following the same procedure described above for **3a**. In this case the mixture was heated at 100 °C for 24 h. Purification was performed by filtration of a dichloromethane solution through a short pad of diatomaceous earth and alumina. Finally, the solvent was evaporated, and the crude product was washed with boiling methanol to give a colorless solid (570 mg, 81%). mp: 187–188 °C. ^1^H NMR (CDCl_3_, 500 MHz) δ: 3.92 (s, 6H, 2 × OCH_3_), 7.02 (m, 1H, ArH), 7.06–7.09 (m, 5H, *J* = 8.5 Hz, ArH), 7.43 (m, 1H, ArH), 7.86 (s, 1H, pyr), 8.16 (B of AB_q_, 4H, *J* = 8.5 Hz, ArH), 8.73 (dd, 1H, *J* = 8.0 Hz, *J* = 2.0 Hz, ArH), 14.02 (br s, 1H, OH). ^13^C NMR and DEPT (CDCl_3_, 125 MHz) δ: 164.8 (C), 163.2 (C), 162.3 (C), 160.9 (C), 133.0 (CH), 129.5 (CH), 129.0 (C), 128.9 (CH), 119.4 (C), 118.9 (CH), 117.7 (CH), 114.5 (CH), 108.4 (CH), 55.5 (CH_3_). MALDI-TOF MS (DHB) *m*/*z*: 385.3 [M + H]^+^. IR (ATR) ν: 1598, 1584, 1509, 1364, 1297, 1235, 1172, 1030, 827, 752 cm^–1^. Anal. Calcd for C_24_H_20_N_2_O_3_: C, 74.98; H, 5.24; N, 7.29. Found: C, 74.74; H, 5.01, N, 7.58%.

## 4 Conclusions

Efficient synthetic routes that combine Suzuki-Miyaura cross coupling and Knoevenagel condensation reactions have been developed for the synthesis of a new family of 2-(2′-hydroxyphenyl)pyrimidines. These compounds exhibited very little or no luminescence both in solution and in the solid state, which is explained by an ESIPT process from the OH group to the nitrogen atoms of the pyrimidine ring and confirmed by the emissive properties of analogous 2′-unsubstituted derivatives. A single-crystal X-ray structure analysis determined inter- and intramolecular interactions and molecular packing structures, which helped us to rationalize the different luminescent behaviors in the solid state. The compounds could be easily protonated at the nitrogen atom of the pyrimidine ring. Protonation provided a substantial enhancement in the fluorescence response of 2-(2′-hydroxyphenyl)pyrimidines and, consequently, allowed us to use these pyrimidines as solid-state acid–base vapor sensors and anticounterfeiting agents. All of the results were interpreted with the aid of extensive DFT and TD-DFT calculations.

## Supporting Information Available

Additional experimental details, material, and methods; synthesis of compounds; values of relative energies for the conformers of compounds **4a**, **4d**, **4e**, **6a**, and **6c**–**6e**; maximum absorption wavelengths, calculated lowest-energy transition wavelengths, oscillator strength and main components of the S_0_ → S_n_ transitions (**4a**, **4d**, **4e**, **6a**, and **6c**–**6e**); wavenumber, reorganization energy, and Huang–Rhys factors (**4a**, **4d**, **4e**, **6a**, and **6c**–**6e**); calculated transition wavelengths and oscillator strengths in the crystal for the S_1_ → S_0_ transitions of the enol and keto forms for the central molecule of 4a and the dimer of 6e; values of relative energies for protonated **4a** and **6a**; maximum absorption wavelengths, calculated lowest-energy transition wavelengths, oscillator strength and main components of the S_0_ → S_n_ transitions (protonated **4a** and **6a**); wavenumber, reorganization energy and Huang–Rhys factors calculated for protonated 4a and 6a; calculated QTAIM parameters of the hydrogen bonds; UV/vis spectra of **4a**–**4c**, **6a**, and **6b** in CH_2_Cl_2_ solution; emission spectra of **4d**, **4e** and **6a**, **6c**, and **6d** in the solid state; bond length and dihedral angles in the S_0_ and S_1_ states for **4a**, **4d**, **4e**, **6a**, and **6c**–**6e**; reorganization energy versus normal mode wavenumbers calculated for the ground state of compounds **4a**, **4d**, **4e**, **6a**, and **6c**–**6e**; atomic displacements of selected vibrational modes calculated for compounds **4a**, **4d**, **4e**, **6a**, and **6c**–**6e**; molecular structure of compounds **4a**, **4e**, and **6c**–**6e** extracted from X-ray analysis; pattern of the π–π interactions between molecules and crystal packing of **6d** and **6e**; potential energy surface of the excited state S_1_ for the dimer **6e**; absorption and emission spectra of **4b** and **6a** upon addition of TFA; ^1^H NMR spectrum of **6a** before and after the addition of an excess of TFA; bond lengths and dihedral angles in the S_0_ and S_1_ states for protonated **4a** and **6a**; frontier molecular orbitals calculated for protonated **4a** and **6a** in the ground state S_0_ and excited state S_1_; potential energy surface of the excited state S_1_ state for protonated **6a**; relative rotational energy barrier of the phenyl ring in the ground state; molecular graphs from the QTAIM analysis; photographs of the reversible color change of **6a** as an anticounterfeiting agent; and ^1^H and ^13^C NMR spectra of all compounds (PDF)
Three-dimensional molecular structures (ZIP)
