# Supplementary material for: Excited-State Intramolecular Proton Transfer in 2-(2′-Hydroxyphenyl)pyrimidines: Synthesis, Optical Properties, and Theoretical Studies
Source: ACS Appl Mater Interfaces. 2022 May 17;14(21):24964–79. doi: 10.1021/acsami.2c05439 (PMC9164210; doi:10.1021/acsami.2c05439)
Supplement: Supplementary file 1 — am2c05439_si_001.pdf [file am2c05439_si_001.pdf]

SUPPORTING INFORMATION

Excited state intramolecular proton transfer (ESIPT) in 2-(2′-hydroxyphenyl)pyrimidines: synthesis, optical properties, and theoretical studies

Rodrigo Plaza-Pedroche,[a] M. Paz Fernández-Liencres,[b] Sonia B. Jiménez-Pulido,[c] Nuria A. Illán-Cabeza,[c] Sylvain Achelle,[d] Amparo Navarro,[b,] and Julián Rodríguez-López[a,*]*

[a] Universidad de Castilla-La Mancha, Área de Química Orgánica, Facultad de Ciencias y Tecnologías Químicas, Avda. Camilo José Cela 10, 13071 Ciudad Real, Spain. E-mail: julian.rodriguez@uclm.es
[b] Universidad de Jaén, Dpto. de Química Física y Analítica, Facultad de Ciencias Experimentales, Campus Las Lagunillas, 23071 Jaén (Spain). E-mail: anavarro@ujaen.es
[c] Universidad de Jaén, Dpto. de Química Inorgánica y Orgánica, Facultad de Ciencias Experimentales, Campus Las Lagunillas, 23071 Jaén (Spain).
[d] Univ Rennes, CNRS, ISCR (Institut des Sciences Chimiques de Rennes) - UMR 6226, F-35000 Rennes, France.

Table of contents

General Information S2
Computational details S2
Crystallography S3
Synthesis of compounds S4-S13
Table S1. Values of relative energies (**4a**, **4d-e**, **6a**, and **6c-e)** S14
Table S2. λ_{ab}^{max}, $\lambda_{vert\text{-}ab}^{calc}$, *f*, and % contribution (**4a**, **4d-e**, **6a**, and **6c-e**) S15
Table S3. v_i, λ_i, and HR factors for **4a**, **4d-e**, **6a**, and **6c-e** (M06-2X/6-31+G**) S16-S17
Table S4. Calculated emission in the crystal for the $S_1 \rightarrow S_0$ transition S18
Table S5. Values of relative energies (protonated **4a** and **6a)** S18
Table S6. λ_{ab}^{max}, $\lambda_{vert\text{-}ab}^{calc}$, *f*, and % contribution (protonated **4a** and **6a**) S18
Table S7. v_i, λ_i, and HR factors for protonated **4a** and **6a** (M06-2X/6-31+G**) S19
Table S8. Calculated QTAIM parameters of the hydrogen bonds S20
Figure S1. UV/vis spectra of **4a-c** and **6a-b** in CH_2Cl_2 solution S21
Figures S2-S4. Emission spectra of **4d-e** and **6a,c-d** in the solid state S22
Figure S5. Bond lengths and dihedral angles in the S_0 and S_1 states for **4a** and **4d-e**. S23
Figure S6. Bond lengths and dihedral angles in the S_0 and S_1 states for **6a** and **6c-e**. S24
Figure S7. Reorganization energy *vs* normal mode wavenumbers (**4a**, **4d-e**, **6a**, and **6c-e**) S25
Figure S8. Atomic displacements of selected vibrational modes (**4a** and **4d-e**). S26
Figure S9. Atomic displacements of selected vibrational modes (**6a** and **6c-e**). S27
Figure S10. Molecular structure of **4a**, **4e**, and **6c-e** extracted from X-ray analysis S27
Figure S11. Pattern of the π-π interactions between molecules and crystal packing of **6d** and **6e** S28
Figure S12. Potential energy surface of the excited state S_1 for dimer **6e** S28
Figures S13-S14. Absorption and emission spectra of **4b** and **6a** upon addition of TFA S29
Figure S15. ^{1}H NMR spectrum of **6a** before and after the addition of an excess of TFA S29
Figure S16. Bond lengths and dihedral angles in the S_0 and S_1 states for protonated **4a** and **6a**. S30
Figure S17. Frontier molecular orbitals calculated for protonated **4a** and **6a** in CH_2Cl_2 solution. S31
Figure S18. Potential energy surface of the excited state S_1 for protonated **6a**. S31
Figure S19. Relative rotational energy barrier of the phenyl ring in the ground state (M06-2X/6-31+G**) ... S32
Figure S20. Molecular graphs from the QTAIM analysis S33
Figure S21. Photographs of the reversible color change of **6a** as counterfeiting agent S34
Figure S22. ^{1}H and ^{13}C NMR spectra of **1b** S35
Figures S23-S25. ^{1}H and ^{13}C NMR spectra of **2a-c** S36-S38
Figures S26-S28. ^{1}H and ^{13}C NMR spectra of **3a-c** S39-S41
Figures S29-S33. ^{1}H and ^{13}C NMR spectra of **4a-e** S42-S46
Figures S34-S36. ^{1}H and ^{13}C NMR spectra of **5a-c** S47-S49
Figures S37-S41. ^{1}H and ^{13}C NMR spectra of **6a-e** S50-S54

General information. All reagents obtained from commercial sources were used as received. All solvents were reagent grade for synthesis and spectroscopic grade for photophysical measurements. A CEM Discover® focused monomode microwave reactor was used for microwave-assisted syntheses. NMR spectra were recorded at room temperature on a Bruker Avance Neo 500 spectrometer. The chemical shifts (δ) are reported in ppm and are referenced internally to the solvent signals of $CDCl_3$ (^1H, 7.27 ppm; ^{13}C, 77.0 ppm) or DMSO-d_6 (^1H, 2.50 ppm; ^{13}C, 39.5 ppm), and externally to $CFCl_3$ (^{19}F, 0.0 ppm). The coupling constants *J* are given in Hz. In the ^1H NMR spectra, the following abbreviations are used to describe the peak patterns: s (singlet), d (doublet), t (triplet), q (quartet), and m (multiplet). In the ^{13}C NMR spectra, the nature of the carbons (C, CH, CH_2 or CH_3) was determined by performing a DEPT experiment. Acidic impurities in $CDCl_3$ were removed by treatment with solid K_2CO_3. Melting points (°C) were measured on a Büchi M-565 apparatus and are uncorrected. MALDI-TOF mass spectra were obtained on a Bruker Autoflex II spectrometer. Elemental analyses were performed in a Thermo Scientific Flash Smart elemental analyzer. UV-visible and fluorescence spectroscopy studies in solution were conducted on a Jasco V-750 spectrophotometer and Jasco FP-8300 spectrofluorometer, respectively. Compounds were excited at their absorption maxima (band of lowest energy) to record the emission spectra. All solutions were measured with optical densities below 0.1. Fluorescence quantum yields (±10%) were determined relative to the indicated reference.

Computational details. The molecular geometries of the ground state, S_0, and the first excited state, S_1, were optimized using the Gaussian16 (revision A.03) suite of programs.[1] The M06-2X[2] and CAM-B3LYP[3] and functionals were chosen along with the 6-31+G** and 6-31G** basis sets. The vibrational modes were calculated for S_0 and S_1 to check the absence of imaginary frequencies. Both the enol and keto forms of each compound have been considered. The solvent environment was described by the polarizable continuum model (PCM) as implemented in the Gaussian package.[4] The ESIPT in solution was investigated by computing the relaxed potential energy scan (PES) from the enol form (E) to the keto form (K) in CH_2Cl_2, constraining one internal coordinate (the oxygen···hydrogen bond length) and optimizing all other coordinates at each scan point. The vertical electronic transitions (absorption and emission) were computed using time dependent (TD)-DFT calculations. The vertical electronic transitions $S_1 \rightarrow S_0$ in solution were calculated as $\Delta E_{em} = E_{S1}(G_{S1}) - E_{S0}(G_{S1})$, where $E_{S1}(G_{S1})$ is the energy of the S_1 state at its equilibrium geometry (state-specific solvation approach)[5] and $E_{S0}(G_{S1})$ is the energy of the S_0 state at the S_1 state geometry and with the static solvation from

[1] Frisch, M. J.; Trucks, G. W.; Schlegel, H. B.; Scuseria, G. E.; Robb, M. A.; Cheeseman, J. R.; Scalmani, G.; Barone, V.; Petersson, G. A.; Nakatsuji, H.; Li, X. et al. Gaussian, Inc., Wallingford CT, 2016.
[2] Zhao, Y.; Truhlar, D. G. *Theor. Chem. Acc.* **2008**, 120, 215–241.
[3] Yanai T.; Tew, D. P.; Handy, N. C. *Chem. Phys. Lett.* **2004**, 393, 51–7.
[4] (a) Cossi, M.; Rega, N.; Scalmani, G.; Barone, V. *J. Comput. Chem.* **2003**, *24*, 669–81. (b) Tomasi, J.; Mennucci, B.; Cammi, R. *Chem. Rev.* **2005**, *105*, 2999–3094. (c) Cammi, R.; Corni, S.; Mennucci, B.; Tomasi, J. *J. Chem. Phys.* **2005**, *122*, 104513.
[5] Improta, R.; Barone, V.; Scalmani, G.; Frisch, M. J. *Chem. Phys.* **2006**, *125*, 054103.

the excited state.[6] The Huang-Rhys (HR) factors for each vibrational mode, S_i, were calculated from the reorganization energy, λ_i, associated to the electronic relaxation using the DUSHIN program[7] which are related as follows: $\lambda = \sum_i \lambda_i = \sum_i \hbar\, \omega_i S_i$, where ω_i is the wavenumber associated to the vibrational mode *i*. For compounds **4a** and **6a**, the keto (S_1) geometry has been considered for the HR factors calculations.

The ONIOM approach[8] was used to simulate the solid state building a model cluster from the X-ray crystal structure. One central molecule (high level) was treated with M06-2X/6-31G** and both S_0 and S_1 electronic state geometries were fully optimized. This central molecule was surrounded by several molecules (low level) which were treated by molecular mechanics (MM) using the UFF[9] force field with their molecular geometries frozen. In addition, TD-DFT calculations were also performed for this central molecule in order to predict the vertical electronic transitions $S_1 \rightarrow S_0$ from the excited state.

The ESIPT in the crystal was investigated by computing the relaxed potential energy scan (PES) from the enol form (E) to the keto form (K), constraining one internal coordinate (the oxygen···hydrogen bond length) and optimizing all other coordinates at each scan point. In the case of compound 4a, the PES was calculated only for the central molecule (intramolecular proton transfer) while in the case of compound 6e, the PES was calculated considering two molecules to simulate the intermolecular proton transfer.

Quantum Theory of Atoms In Molecules (QTAIM) calculations in the context of the Bader's theory were performed using the wavefunction generated by Gaussian16 as input for the AIM2000 program.[10]

Crystallography. The X-ray data were collected with a Bruker-Apex-II CCD diffractometer with graphite monochromated Mo-K_α (λ= 0.71073 Å) radiation at 100 K. Lorentz, polarization and multiscan absorption corrections were applied with SADABS.[11] The structures were solved by conventional direct methods and refined using SHELXL-2018/3[12] integrated in the WinGX 2021.3[13] employing full-matrix least-squares methods on F^2. All non-H atoms were refined anisotropically; hydrogen atoms have been located and refined isotropically, except in compound **4a**, where some of them were placed in idealized positions and treated using riding models. The structural models were analyzed with PLATON[14] and graphics were obtained

---

[6] Scalmani, G.; Frisch, M. J.; Mennucci, B.; Tomasi, J.; Cammi, R.; Barone, V. *J. Chem. Phys.* **2006**, *124*, 094107.

[7] Reimers, J. R. *J. Chem. Phys.* **2001**, *115*, 9103-9109.

[8] (a) Dapprich, S.; Komáromi, I.; Byun, K. S.; Morokuma, K.; Frisch, M. J. *J. Mol. Struct. (Theochem)* **1999**, *461-462*, 1–21. (b) Vreven, T.; Morokuma, K.; Farkas, O.; Schlegel, H. B.; Frisch, M. J. *J. Comput. Chem.* **2003**, *24*, 760–769. (c) Lin, H.; Truhlar, D. *Theor. Chem. Acc.* **2007**, *117*, 185–199.

[9] Casewit, C. J.; Colwell, K. S.; Rappe, A. K. *J. Am. Chem. Soc.* **1992**, *114*, 10035–10046.

[10] (a) Biegler-Köning, F.; Schönbohm, J.; Bayles, D. *J. Comput. Chem.* **2001**, *22*, 545-559. (b) Biegler-Köning, F.; Schönbohm, J. *J. Comput. Chem.* **2002**, *23*, 1489-1494.

[11] SADABS, version 2016/2. Bruker AXS Inc., Madison, WI, USA, 2016.

[12] Sheldrick, G. M. SHELXL-2018/3. University of Göttingen, Göttingen, Germany, 2018.

[13] Farrugia, L. J. *J. Appl. Cryst.* **2012**, *45*, 849−854.

[14] Spek, A. L. PLATON, a multipurpose crystallographic tool. Utrecht University, Utrecht, The Netherlands, 2003.

using MERCURY.[15] The CIF files have been deposited at the Cambridge Crystallographic Data Center and allocated the deposition numbers CCDC 2113241 (**4a**), 2113242 (**4e**), 2113243 (**6c**), 2113244 (**6d**), and 2113245 (**6e**).

Synthesis of compounds

*2-Iodo-4,6-dimethylpyrimidine (**1b**).*

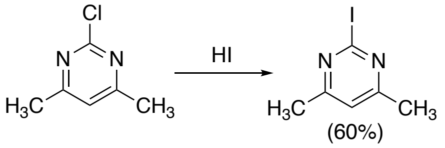

Compound **1b** was prepared according to a previously reported procedure.[16] A heterogeneous mixture of 2-chloro-4,6-dimethylpyrimidine (2 g, 14 mmol) and hydroiodic acid (5 mL, 57%) was vigorously stirred at room temperature for 16 h. The suspension was neutralized with saturated aqueous solution of K_2CO_3 and solid $Na_2S_2O_3 \cdot 5H_2O$ was added until decolorization. After diluting with water, the white precipitated was collected by filtration, washed, dried, and purified by crystallization from hexanes. Compound **1b** was obtained as colorless needles (1.96 g, 60%). ^{1}H-RMN ($CDCl_3$, 500 MHz) δ: 2.43 (s, 6H, 2xCH_3), 7.00 (s, 1H, H5). ^{13}C-RMN and DEPT ($CDCl_3$, 125 MHz) δ: 168.9 (C4, C6), 129.5 (C2), 119.7 (C5), 23.7 (CH_3).

*(E,E)-2-Chloro-4,6-bis(4'-methoxystyryl)pyrimidine (**2a**).*

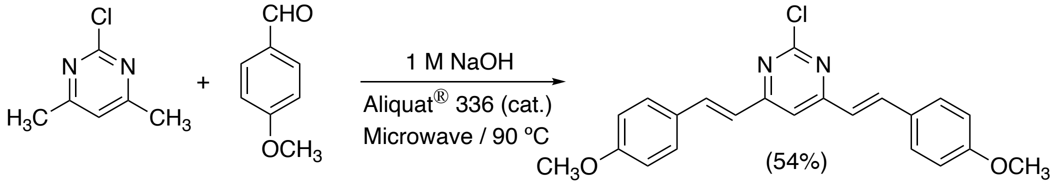

A microwave reactor tube was loaded with a mixture of 2-chloro-4,6-dimethylpyrimidine (500 mg, 3.5 mmol), 4-methoxybenzaldehyde (952 mg, 7.0 mmol), aliquat® 336 (155 mg, 0.35 mmol), and 1 M NaOH (6 mL). The tube was sealed with a snap cap and the reaction mixture was stirred and irradiated at 90 ºC (external surface sensor) for 5 min (power 200 W, pressure 250 psi). After cooling, the precipitated solid was filtered off, dissolved in CH_2Cl_2 and filtered through a short pad of neutral alumina. The solvent was evaporated, and the yellow solid was washed with boiling MeOH (710 mg, 54%). ^{1}H NMR ($CDCl_3$, 500 MHz) δ: 3.86 (s, 6H, 2×OCH_3), 6.88 (A of AB_q, 2H, J = 16.0 Hz, 2×CH=), 6.94 (A of AB_q, 4H, J = 8.5 Hz, ArH), 7.10 (s, 1H, pyr), 7.56 (B of AB_q, 4H, J = 8.5 Hz, ArH), 7.89 (B of AB_q, 2H, J = 16.0 Hz, 2×CH=). ^{13}C NMR and DEPT ($CDCl_3$, 125 MHz) δ: 165.8 (C), 161.4 (C), 161.0 (C), 138.2 (CH), 129.4 (CH), 128.1 (C), 122.2 (CH), 114.4 (CH), 114.0 (CH), 55.4 (CH_3). MALDI-TOF MS (dithranol) *m/z*: 379.3 $[M+H]^+$. Anal. Calcd for $C_{22}H_{19}ClN_2O_2$: C, 69.75; H, 5.06; N, 7.39. Found: C, 69.94; H, 4.97, N, 7.41.

---

[15] Macrae, C. F.; Sovago, I.; Cottrell, S. J.; Galek, P. T. A.; McCabe, P.; Pidcock, E.; Platings, M.; Shields, G. P.; Stevens, J. S.; Towler, M.; Wood, P. A. *J. Appl. Cryst.* **2020**, *53*, 226−235.
[16] Vlád, G.; Horváth, I. T. *J. Org. Chem.* **2002**, *67*, 6550-6552.

*(E,E)-2-Iodo-4,6-bis(4'-methoxystyryl)pyrimidine (**2b**).*

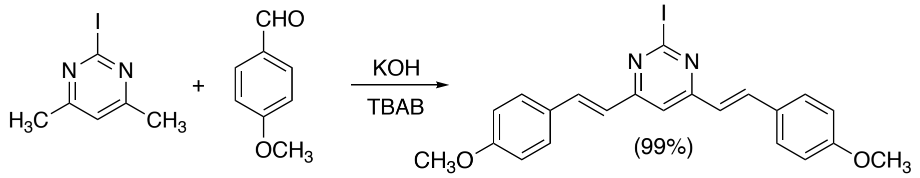

Compound **2b** was prepared according to a slightly modified procedure previously reported.[17] A solid mixture of 2-iodo-4,6-dimethylpyrimidine (1.4 g, 5.98 mmol), powdered potassium hydroxide (1.68 g, 30 mmol), and tetrabutylammonium bromide (98 mg, 0.3 mmol) was stirred a room temperature for 30 min. Then, 4-methoxybenzaldehyde (1.96 g, 14.4 mmol) was added and the stirring was continued for 23 h. Dichloromethane was used to dissolve the product and the insoluble KOH was eliminated by filtration. The solvent was evaporated to afford a yellow solid that was washed successively with water, ethanol, and boiling ethyl ether (1.40 g, 99%). ^{1}H NMR ($CDCl_3$, 500 MHz) δ: 3.86 (s, 6H, 2×OCH_3), 6.80 (A of AB_q, 2H, J = 16.0 Hz, 2×CH=), 6.94 (A of AB_q, 4H, J = 8.5 Hz, ArH), 7.15 (s, 1H, pyr), 7.55 (B of AB_q, 4H, J = 8.5 Hz, ArH), 7.82 (B of AB_q, 2H, J = 16.0 Hz, 2×CH=). ^{13}C NMR and DEPT ($CDCl_3$, 125 MHz) δ: 164.7 (C), 160.9 (C), 138.0 (CH), 130.5 (C), 129.4 (CH), 128.2 (C), 122.3 (CH), 114.5 (CH), 114.4 (CH), 55.4 (CH_3). MALDI-TOF MS (dithranol) *m/z*: 471.2 $[M+H]^+$. Anal. Calcd for $C_{22}H_{19}IN_2O_2$: C, 56.18; H, 4.07; N, 5.96. Found: C, 55.99; H, 3.97, N, 6.20.

*(E,E)-2-Chloro-4,6-bis(4'-dimethylaminostyryl)pyrimidine (**2c**).*[18]

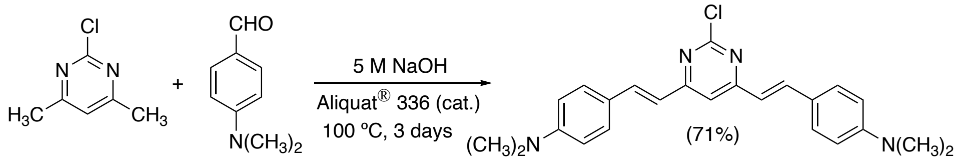

A stirred mixture of 2-chloro-4,6-dimethylpyrimidine (300 mg, 2.1 mmol), 4-dimethylaminobenzaldehyde (627 mg, 4.2 mmol), aliquat® 336 (93 mg, 0.21 mmol), and 5 M NaOH (10 mL) was heated at 100 ºC in a flask sealed with a screw cap for 3 days. After cooling, the red precipitated solid was filtered off and purified by washing with water and boiling methanol (600 mg, 71%). Further purification was achieved by crystallization from $CHCl_3$/EtOH. ^{1}H NMR ($CDCl_3$, 500 MHz) δ: 3.04 (s, 12H, 2×NMe_2), 6.71 (A of AB_q, 4H, J = 9.0 Hz, ArH), 6.80 (A of AB_q, 2H, J = 16.0 Hz, 2×CH=), 7.05 (s, 1H, pyr), 7.51 (B of AB_q, 4H, J = 9.0 Hz, ArH), 7.84 (B of AB_q, 2H, J = 16.0 Hz, 2×CH=). ^{13}C NMR and DEPT ($CDCl_3$, 125 MHz) δ: 166.0 (C), 161.3 (C), 151.4 (C), 138.7 (CH), 129.4 (CH), 123.4 (C), 119.7 (CH), 113.1 (CH), 112.0 (CH), 40.2 (CH_3). MALDI-TOF MS (dithranol) *m/z*: 379.3 $[M+H]^+$.

---

[17] Martin, F.-A; Baudequin, C.; Fiol-Petit, C.; Darabantu, M.; Ramondenc, Y.; Plé, N. *Tetrahedron* **2014**, *70*, 2546-2555.
[18] Brown, D. M.; Kon, G. A. R. *J. Chem. Soc.* **1948**, 2147-2154.

*2-(2'-Hydroxyphenyl)-4,6-dimethylpyrimidine (**3a**).*[19]

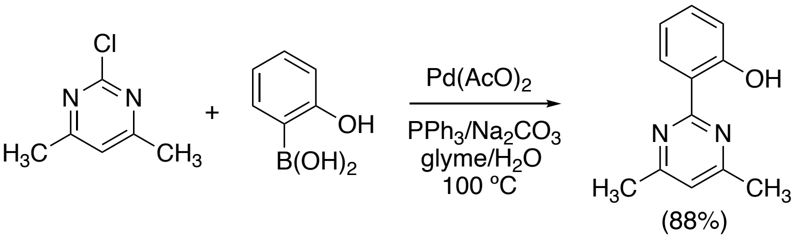

2-Chloro-4,6-dimethylpyrimidine (1000 mg, 7.02 mmol), 2-hydroxyphenylboronic acid (1065 mg, 7.72 mmol), and sodium carbonate (3720 mg, 35.1 mmol, dissolved in a minimum amount of water) were mixed with 1,2-dimethoxyethane (8 mL). Palladium acetate (79 mg, 0.35 mmol) and triphenylphosphine (183 mg, 0.70 mmol) were then added. The mixture was bubbled with argon for 5 min and heated at 100 ºC in a flask sealed with a screw cap for 48 h. The solvent was evaporated, water was added, and the mixture extracted with dichloromethane (×3). The combined organic extracts were dried ($MgSO_4$) and the solvent evaporated. The crude product was purified by column chromatography (alumina, hexanes/EtAcO mixtures, 10:0 to 9:1) to give a colorless solid (1230 mg, 88%). Further purification was achieved by crystallization from hexanes. ^1H NMR ($CDCl_3$, 500 MHz) δ: 2.56 (s, 6H, 2×CH_3), 6.94 (s, 1H, pyr), 6.94-6.97 (m, 1H, ArH), 7.02 (dd, 1H, J = 8.0 Hz, J = 1.0 Hz, ArH), 7.38 (m, 1H, ArH), 8.54 (dd, 1H, J = 8.0 Hz, J = 1.5 Hz, ArH). ^{13}C NMR and DEPT ($CDCl_3$, 125 MHz) δ: 165.9 (C), 164.2 (C), 160.7 (C), 133.0 (CH), 129.2 (CH), 118.9 (CH), 118.6 (C), 117.8 (CH), 117.5 (CH), 23.9 (CH_3). IR (ATR) ν: 1561, 1434, 1366, 1250, 848, 759 cm^{-1}.

*4,6-Dimethyl-2-phenylpyrimidine (**3b**).*[20]

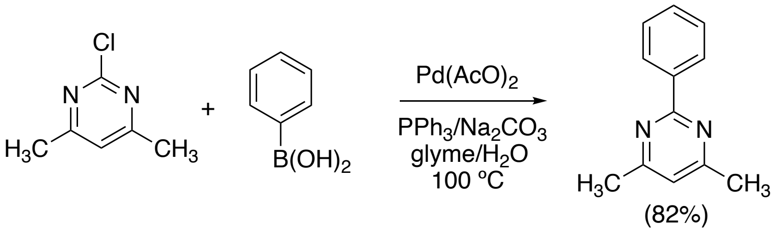

This compound was prepared from 2-chloro-4,6-dimethylpyrimidine (500 mg, 3.51 mmol) and phenylboronic acid (470 mg, 3.85 mmol) following the same procedure described above for **3a**. The crude product was purified by column chromatography (alumina, hexanes) to give a colorless oil which solidified upon standing (530 mg, 82%). ^1H NMR ($CDCl_3$, 500 MHz) δ: 2.56 (s, 6H, 2×CH_3), 6.94 (s, 1H, pyr), 7.46-7.49 (m, 3H, ArH), 8.43-8.45 (m, 2H, ArH). ^{13}C

[19] Stolle, W. A. W.; Frissen, A. E.; Marcelis, A. T. M.; van der Plas, H. C. *J. Org. Chem.* **1992**, *57*, 3000-3007.

[20] (a) Schmitt, J.-L.; Stadler, A.-M.; Kyritsakas, N.; Lehn, J.-M. *Helv. Chim. Acta* **2003**, *86*, 1598-1624. (b) Vadagaonkar, K. S.; Kalmode, H. P.; Murugan, K.; Chaskar, A. C. *Lett. Org. Chem.* **2015**, *12*, 447-458. (c) Vadagaonkar, K. S.; Kalmode, H. P.; Prakash, S.; Chaskar, A. C. *New J. Chem.* **2015**, *39*, 3639-3645. (d) Chowrasia, R.; Katla, R.; Darbem, M. P.; Branquinho, T. A.; Rufino de Oliveira, A.; Manjari, P. S.; Domingues, N. L. C. *Tetrahedron Lett.* **2016**, *57*, 1656-1660. (e) Chu, X.-Q.; Cao, W.-B.; Xu, X.-P.; Ji, S.-J. *J. Org. Chem.* **2017**, *82*, 1145-1154.

NMR and DEPT ($CDCl_3$, 125 MHz) δ: 166.8 (C), 164.1 (C), 137.9 (C), 130.4 (CH), 128.4 (CH), 128.3 (CH), 118.0 (CH), 24.1 (CH_3).

*2-(4'-Methoxyphenyl)-4,6-dimethylpyrimidine (**3c**).*[21]

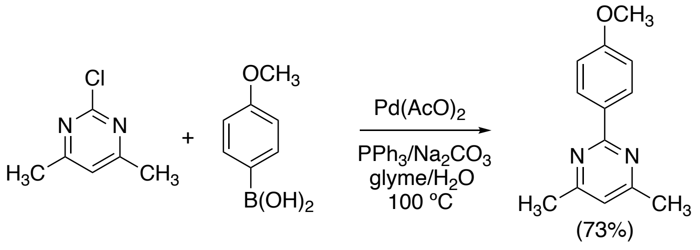

This compound was prepared from 2-chloro-4,6-dimethylpyrimidine (500 mg, 3.51 mmol) and 4-methoxyphenylboronic acid (561 mg, 3.69 mmol) following the same procedure described above for **3a**. The crude product was purified by column chromatography (alumina, hexanes to hexanes/EtAcO, 9:1) and recrystallized from hexanes to give a colorless solid (550 mg, 73%). ^{1}H NMR ($CDCl_3$, 500 MHz) δ: 2.51 (s, 6H, 2×CH_3), 3.87 (s, 3H, OCH_3), 6.86 (s, 1H, pyr), 6.98 (A of AB_q, 2H, J = 9.0 Hz, ArH), 8.41 (B of AB_q, 2H, J = 9.0 Hz, ArH). ^{13}C NMR and DEPT ($CDCl_3$, 125 MHz) δ: 166.5 (C), 163.9 (C), 161.5 (C), 130.8 (C), 129.8 (CH), 117.2 (CH), 113.7 (CH), 55.3 (OCH_3), 24.1 (CH_3).

*(E,E)-2-(2'-Hydroxyphenyl)-4,6-bis(4'-methoxystyryl)pyrimidine (**4a**).*

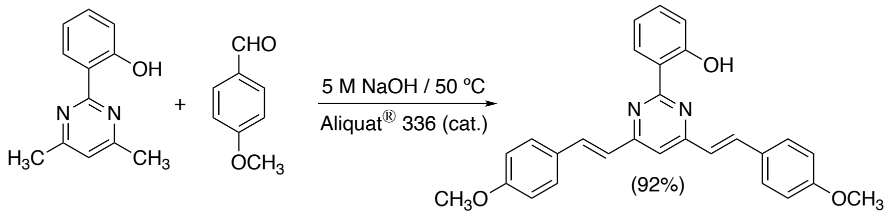

A stirred mixture of 2-(2′-hydroxyphenyl)-4,6-dimethylpyrimidine (100 mg, 0.5 mmol), 4-methoxybenzaldehyde (136 mg, 1 mmol), aliquat® 336 (22 mg, 0.05 mmol), and 5 M NaOH (8 mL) was heated at 50 ºC for 22 h. After cooling, the precipitated yellow solid was collected by filtration and purified by washing with boiling methanol (200 mg, 92%). Mp: 175-177 ºC (EtAcO/MeOH). ^{1}H NMR ($CDCl_3$, 500 MHz) δ: 3.86 (s, 6H, 2×OCH_3), 6.94 (A of AB_q, 4H, J = 8.5 Hz, ArH), 6.97 (A of AB_q, 2H, J = 16.0 Hz, 2×CH=), 6.99-7.02 (m, 1H, ArH), 7.07 (dd, 1H, J = 8.0 Hz, ArH), 7.10 (s, 1H, pyr), 7.39-7.43 (m, 1H, ArH), 7.58 (B of AB_q, 4H, J = 8.5 Hz, ArH), 7.84 (B of AB_q, 2H, J = 16.0 Hz, 2×CH=), 8.67 (dd, 1H, J = 8.0 Hz, J = 1.5 Hz, ArH), 13.95 (br s, 1H, OH). ^{13}C NMR and DEPT ($CDCl_3$, 125 MHz) δ: 164.1 (C), 161.9 (C), 161.0 (C), 160.8 (C), 137.5 (CH), 133.0 (CH), 129.4 (CH), 128.2 (C), 122.8 (CH), 119.0 (C), 118.9 (CH), 117.7 (CH), 114.4 (CH), 113.1 (CH), 55.4 (CH_3). MALDI-TOF MS (DHB) *m/z*: 437.3 $[M+H]^+$. IR (ATR) ν: 1526, 1593, 1560, 1508, 1367, 1242, 1161, 1022, 972, 840, 754 cm^{-1}. Anal. Calcd for $C_{28}H_{24}N_2O_3$: C, 77.04; H, 5.54; N, 6.42. Found: C, 76.86; H, 5.39, N, 6.62.

[21] (a) Dutta, M.; Movassat, M.; Brook, D. J. R.; Oliver, A.; Ward, D. *Supramol. Chem.* **2011**, *23*, 630-640. (b) Yang, B.; Wang, Z.-X. *Org. Lett.* **2017**, *19*, 6220-6223.

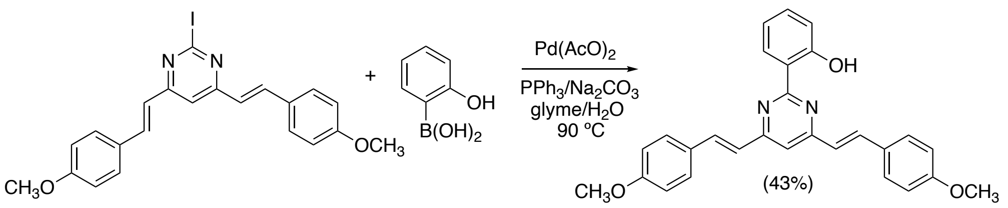

Compound **4a** could also be prepared from the iodo derivative **2b** (150 mg, 0.32 mmol) and 2-hydroxyphenylboronic acid (52 mg, 0.38 mmol) following a similar procedure to that described above for **3a**. In this case the mixture was heated at 90 ºC for 24 h. The crude product was washed with MeOH and purified by crystallization from EtAcO/MeOH (60 mg, 43%).

*(E,E)-2-(2'-Hydroxyphenyl)-4,6-bis(4'-dimethylaminostyryl)pyrimidine (**4b**).*

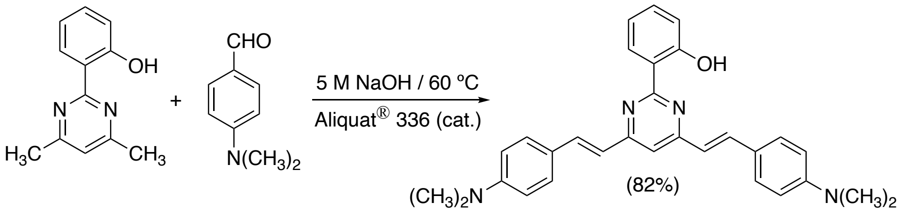

A stirred mixture of 2-(2′-hydroxyphenyl)-4,6-dimethylpyrimidine (100 mg, 0.5 mmol), 4-dimethylaminobenzaldehyde (164 mg, 1 mmol), aliquat® 336 (22 mg, 0.05 mmol), and 5 M NaOH (8 mL) was heated at 60 ºC for 23 h. After cooling, the orange precipitated solid was collected by filtration and purified by washing with boiling methanol (190 mg, 82%). Mp: 243-245 ºC (EtAcO). ^{1}H NMR ($CDCl_3$, 500 MHz) δ: 3.04 (s, 6H, 2×CH_3), 6.73 (A of AB_q, 4H, J = 8.5 Hz, ArH), 6.89 (A of AB_q, 2H, J = 16.0 Hz, 2×CH=), 7.00 (t, 1H, J = 8.0 Hz, ArH), 7.05 (s, 1H, pyr), 7.06 (d, 1H, J = 8.0 Hz, ArH), 7.38-7.42 (m, 1H, ArH), 7.55 (B of AB_q, 4H, J = 8.5 Hz, ArH), 7.84 (B of AB_q, 2H, J = 16.0 Hz, 2×CH=), 8.67 (dd, 1H, J = 7.5 Hz, J = 1.5 Hz, ArH), 14.34 (br s, 1H, OH). ^{13}C NMR and DEPT ($CDCl_3$, 125 MHz) δ: 164.2 (C), 162.1 (C), 160.9 (C), 151.2 (C), 137.7 (CH), 132.5 (CH), 129.3 (CH), 129.3 (CH), 123.7 (C), 120.5 (CH), 119.6 (C), 118.6 (CH), 117.6 (CH), 112.6 (CH), 112.1 (CH), 40.2 (CH_3). MALDI-TOF MS (DHB) *m/z*: 463.4 $[M+H]^+$. IR (ATR) ν: 1600, 1557, 1511, 1488, 1355, 1146, 798, 748 cm^{-1}. Anal. Calcd for $C_{30}H_{30}N_4O$: C, 77.89; H, 6.54; N, 12.11. Found: C, 77.68; H, 6.30, N, 12.33.

*(E,E)- 4,6-Bis(4'-trifluoromethylstyryl)-2-(2'-hydroxyphenyl)pyrimidine (**4c**).*

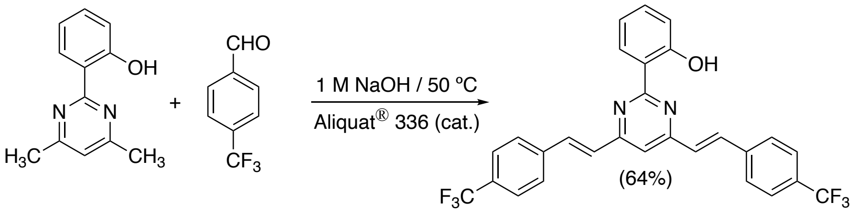

A stirred mixture of 2-(2′-hydroxyphenyl)-4,6-dimethylpyrimidine (300 mg, 1.5 mmol), 4-trifluoromethylbenzaldehyde (576 mg, 3.3 mmol), aliquat® 336 (66 mg, 0.15 mmol), and 1 M NaOH (8 mL) was heated at 50 ºC for 20 h. After cooling, the pale-yellow precipitated solid was collected by filtration and purified by washing with ethanol (510 mg, 64%). Mp: 166-168 ºC (EtAcO/EtOH). ^{1}H NMR ($CDCl_3$, 500 MHz) δ: 7.04 (m, 1H, ArH), 7.08 (dd, 1H, J = 8.0 Hz, J = 1.0 Hz, ArH), 7.19 (A of AB_q, 2H, J = 16.0 Hz, 2×CH=), 7.24 (s, 1H, pyr), 7.44 (m,

1H, ArH), 7.69 (A of AB_q, 4H, J = 8.0 Hz, ArH), 7.74 (B of AB_q, 4H, J = 8.0 Hz, ArH), 7.92 (B of AB_q, 2H, J = 16.0 Hz, 2×CH=), 8.65 (dd, 1H, J = 8.0 Hz, J = 1.5 Hz, ArH). ^{13}C NMR and DEPT ($CDCl_3$, 125 MHz) δ: 164.0 (C), 161.4 (C), 160.7 (C), 138.5 (C), 137.0 (CH), 133.8 (CH), 131.4 (q, J = 32.3 Hz, C), 129.6 (CH), 128.0 (CH), 126.8 (CH), 125.9 (q, J = 3.7 Hz, CH), 123.8 (q, J = 270.6 Hz, C), 119.3 (CH), 118.1 (C), 118.0 (CH), 114.2 (CH). ^{19}F NMR ($CDCl_3$, 471 MHz) δ: –62.8. MALDI-TOF MS (DHB) *m/z*: 513.2 $[M+H]^+$. IR (ATR) ν: 1628, 1565, 1524, 1496, 1320, 1107, 1065, 965, 950, 848, 764 cm^{-1}. Anal. Calcd for $C_{28}H_{18}F_6N_2O$: C, 65.63; H, 3.54; N, 5.47. Found: C, 65.52; H, 3.60, N, 5.68.

*(E,E)-4,6-Bis(4'-methoxystyryl)-2-phenylpyrimidine (**4d**).*

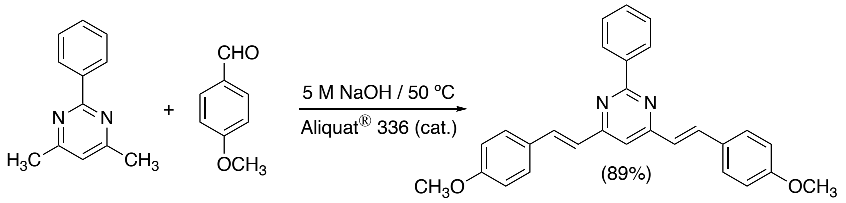

A stirred mixture of 4,6-dimethyl-2-phenylpyrimidine (300 mg, 1.63 mmol), 4-methoxybenzaldehyde (466 mg, 3.42 mmol), aliquat® 336 (72 mg, 0.16 mmol), and 5 M NaOH (10 mL) was heated at 50 ºC for 23 h. After cooling, the precipitated yellow solid was collected by filtration and purified by washing with boiling methanol (610 mg, 89%). Mp: 171-174 ºC (EtAcO/MeOH). ^{1}H NMR ($CDCl_3$, 500 MHz) δ: 3.87 (s, 6H, 2×OCH_3), 6.96 (A of AB_q, 4H, J = 9.0 Hz, ArH), 7.03 (A of AB_q, 2H, J = 16.0 Hz, 2×CH=), 7.13 (d, 1H, J = 2.0 Hz, pyr), 7.48-7.56 (m, 3H, Ar), 7.61 (B of AB_q, 4H, J = 9.0 Hz, ArH), 8.01 (B of AB_q, 2H, J = 16.0 Hz, 2×CH=), 8.62 (dd, 2H, J = 8.5 Hz, J = 1.5 Hz, ArH). ^{13}C NMR and DEPT ($CDCl_3$, 125 MHz) δ: 164.0 (C), 163.1 (C), 160.5 (C), 138.4 (C), 136.0 (CH), 130.3 (CH), 129.1 (CH), 128.8 (C), 128.4 (CH), 124.3 (CH), 114.3 (CH), 113.7 (CH), 55.3 (CH_3). MALDI-TOF MS (dithranol) *m/z*: 421.3 $[M+H]^+$. Anal. Calcd for $C_{28}H_{24}N_2O_2$: C, 79.98; H, 5.75; N, 6.66. Found: C, 79.79; H, 5.51, N, 6.89.

*(E,E)-2-(4'-methoxyphenyl)-4,6-Bis(4'-methoxystyryl)pyrimidine (**4e**).*

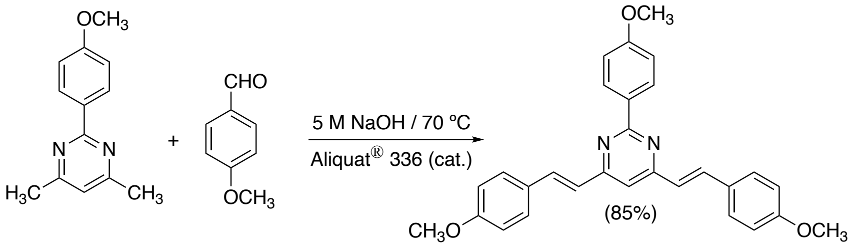

A stirred mixture of 2-(4′-methoxyphenyl)-4,6-dimethylpyrimidine (100 mg, 0.47 mmol), 4-methoxybenzaldehyde (127 mg, 0.94 mmol), aliquat® 336 (21 mg, 0.05 mmol), and 5 M NaOH (10 mL) was heated at 70 ºC for 23 h. After cooling, the precipitated solid was collected by filtration and purified by washing with boiling methanol (180 mg, 85%). Mp: 148.5-150.4 ºC. ^{1}H NMR ($CDCl_3$, 500 MHz) δ: 3.86 (s, 6H, 2×OCH_3), 3.91 (s, 3H, OCH_3), 6.95 (A of AB_q, 4H, J = 8.5 Hz, ArH), 7.00 (A of AB_q, 2H, J = 16.0 Hz, 2×CH=), 7.05 (A of AB_q, 2H, J = 9.0 Hz, ArH), 7.07 (s, 1H, pyr), 7.60 (B of AB_q, 4H, J = 8.5 Hz, ArH), 7.97 (B of AB_q, 2H, J = 16.0

Hz, 2×CH=), 8.59 (B of AB_q, 2H, $J = 9.0$ Hz, ArH). ^{13}C NMR and DEPT ($CDCl_3$, 125 MHz) δ: 163.8 (C), 162.9 (C), 161.6 (C), 160.4 (C), 135.8 (CH), 131.1 (C), 129.9 (CH), 129.0 (CH), 128.8 (C), 124.5 (CH), 114.3 (CH), 113.7 (CH), 113.1 (CH), 55.3 (CH_3). MALDI-TOF MS (dithranol) *m/z*: 451.4 $[M+H]^+$. Anal. Calcd for $C_{29}H_{26}N_2O_3$: C, 77.31; H, 5.82; N, 6.22. Found: C, 77.06; H, 5.68, N, 6.45.

*2-Chloro-4,6-bis(4'-methoxyphenyl)pyrimidine (**5a**).*[22]

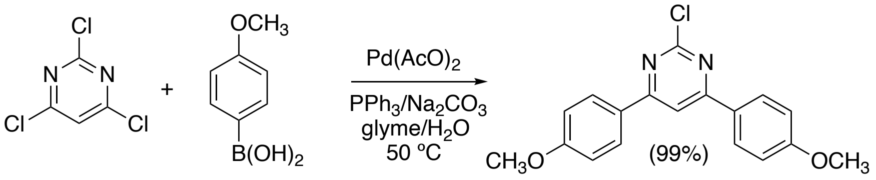

2,4,6-Trichloropyrimidine (500 mg, 2.73 mmol), 4-methoxyphenylboronic acid (830 mg, 5.46 mmol), and sodium carbonate (1810 mg, 17.1 mmol, dissolved in a minimum amount of water) were mixed with 1,2-dimethoxyethane (10 mL). Palladium acetate (15 mg, 0.068 mmol) and triphenylphosphine (36 mg, 0.137 mmol) were then added. The mixture was bubbled with argon for 10 min and heated at 50 ºC in a flask sealed with a screw cap for 24 h. The solvent was evaporated, water was added, and the mixture extracted with dichloromethane (×3). The combined organic extracts were dried ($MgSO_4$), concentrated under vacuum and filtered through a short pad of Celite and alumina. Finally, the solvent was evaporated and the crude product washed with boiling methanol to give a colorless solid (880 mg, 99%). Further purification was achieved by crystallization from EtAcO/hexanes. ^{1}H NMR ($CDCl_3$, 500 MHz) δ: 3.91 (s, 6H, 2×CH_3), 7.03 (A of AB_q, 4H, $J = 9.0$ Hz, ArH), 7.88 (s, 1H, pyr), 8.13 (B of AB_q, 4H, $J = 9.0$ Hz, ArH). ^{13}C NMR and DEPT ($CDCl_3$, 125 MHz) δ: 166.7 (C), 162.5 (C), 161.8 (C), 129.0 (CH), 128.2 (C), 114.4 (CH), 109.0 (CH), 55.5 (CH_3).

*2-Chloro-4,6-bis(4'-trifluoromethylphenyl)pyrimidine (**5b**).*[23]

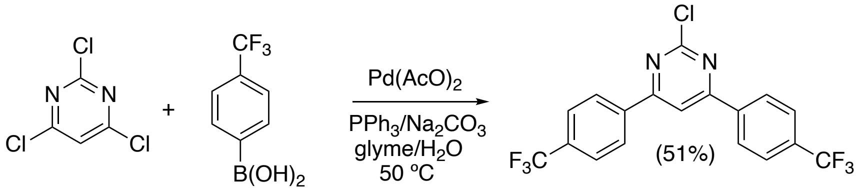

This compound was prepared from 2,4,6-trichloropyrimidine (150 mg, 2.73 mmol) and 4-(trifluoromethyl)phenylboronic acid (312 mg, 1.64 mmol) following a similar procedure to that described above for **5a**. In this case the mixture was heated at 50 ºC for 21 h. The crude product was purified by column chromatography (alumina, hexanes/EtAcO mixtures, 9.5:0.5 to 8:2) to

[22] (a) Achelle, S.; Ramondenc, Y.; Marsais, F.; Plé, N. *Eur. J. Org. Chem.* **2008**, 3129-3007. (b) Kayamba, F.; Malimabe, T.; Ademola, I. K.; Pooe, O. J.; Kushwaha, N. D.; Mahlalela, M.; van Zyl, R. L.; Gordon, M.; Mudau, P. T.; Zininga, T.; Shonhai, A.; Nyamori, V. O.; Karpoormath, R. *Eur. J. Med. Chem.* **2021**, *217*, 113330.

[23] Trist, I. M. L.; Nannetti, G.; Tintori, C.; Fallacara, A. L.; Deodato, D.; Mercorelli, B.; Palù, G.; Wijtmans, M.; Gospodova, T.; Edink, E.; Verheij, M.; de Esch, I.; Viteva, L.; Loregian, A.; Botta, M. *J. Med. Chem.* **2016**, *59*, 2688-2703.

give a colorless oil which solidified upon standing (170 mg, 51%). ^{1}H NMR ($CDCl_3$, 500 MHz) δ: 7.79 (A of AB_q, 4H, J = 8.5 Hz, ArH), 8.05 (s, 1H, pyr), 8.25 (B of AB_q, 4H, J = 8.5 Hz, ArH). ^{13}C NMR and DEPT ($CDCl_3$, 125 MHz) δ: 166.4 (C), 162.4 (C), 138.5 (C), 133.4 (q, J = 32.5 Hz, C), 127.8 (CH), 126.0 (q, J = 3.6 Hz, CH), 123.7 (q, J = 270.7 Hz, CF_3), 111.4 (CH). ^{19}F NMR ($CDCl_3$, 471 MHz) δ: – 63.0. MALDI-TOF MS (DHB) *m/z*: 403.2 $[M+H]^+$.

*2-Chloro-4,6-bis[4'-(9H-carbazol-9-yl)phenyl]pyrimidine (**5c**).*

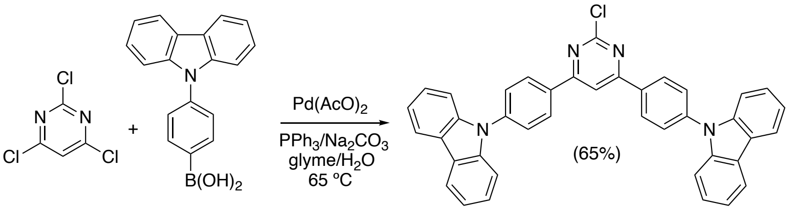

This compound was prepared from 2,4,6-trichloropyrimidine (500 mg, 2.73 mmol) and [4-(9H-carbazol-9-yl)phenyl]boronic acid (1570 mg, 5.46 mmol) following a similar procedure to that described above for **5a**. In this case the mixture was heated at 65 ºC for 16 h. The crude product was washed with boiling methanol to give a yellow solid (1.06 g, 65%). Further purification was achieved by crystallization from $CHCl_3$/hexanes. ^{1}H NMR ($CDCl_3$, 500 MHz) δ: 7.35 (dt, 4H, J = 8.0 Hz, J = 1.0 Hz, ArH), 7.47 (dt, 4H, J = 8.0 Hz, J = 1.0 Hz, ArH), 7.54 (d, 4H, J = 8.0 Hz, ArH), 7.83 (A of AB_q, 4H, J = 8.5 Hz, ArH), 8.18 (d, 4H, J = 8.0 Hz, ArH), 8.21 (s, 1H, pyr), 8.46 (B of AB_q, 4H, J = 8.5 Hz, ArH). ^{13}C NMR and DEPT ($CDCl_3$, 125 MHz) δ: 166.8 (C), 162.4 (C), 141.1 (C), 140.3 (C), 134.2 (C), 129.1 (CH), 127.2 (CH), 126.2 (CH) 123.8 (C), 120.5 (CH), 120.5 (CH), 110.7 (CH), 109.7 (CH). MALDI-TOF MS (DHB) *m/z*: 597.3 $[M+H]^+$. Anal. Calcd for $C_{40}H_{25}ClN_4$: C, 80.46; H, 4.22; N, 9.38. Found: C, 80.27; H, 4.04; N, 9.58.

*2-(2'-Hydroxyphenyl)-4,6-bis(4'-methoxyphenyl)pyrimidine (**6a**).*

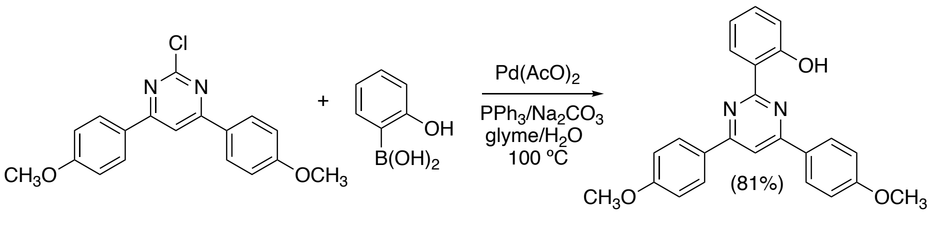

This compound was prepared from the pyrimidine derivative **5a** (640 mg, 2.02 mmol) and 2-hydroxyphenylboronic acid (306 mg, 2.22 mmol) following the same procedure described above for **3a**. In this case the mixture was heated at 100 ºC for 24 h. Purification was performed by filtration of a dichloromethane solution through a short pad of Celite and alumina. Finally, the solvent was evaporated and the crude product washed with boiling methanol to give a colorless solid (570 mg, 81%). Mp: 187-188 ºC. ^{1}H NMR ($CDCl_3$, 500 MHz) δ: 3.92 (s, 6H, 2×OCH_3), 7.02 (m, 1H, ArH), 7.06-7.09 (m, 5H, J = 8.5 Hz, ArH), 7.43 (m, 1H, ArH), 7.86 (s, 1H, pyr), 8.16 (B of AB_q, 4H, J = 8.5 Hz, ArH), 8.73 (dd, 1H, J = 8.0 Hz, J = 2.0 Hz, ArH), 14.02 (br s, 1H, OH). ^{13}C NMR and DEPT ($CDCl_3$, 125 MHz) δ: 164.8 (C), 163.2 (C), 162.3 (C), 160.9 (C), 133.0 (CH), 129.5 (CH), 129.0 (C), 128.9 (CH), 119.4 (C), 118.9 (CH), 117.7 (CH), 114.5 (CH), 108.4 (CH), 55.5 (CH_3). MALDI-TOF MS (DHB) *m/z*: 385.3 $[M+H]^+$. IR

(ATR) ν: 1598, 1584, 1509, 1364, 1297, 1235, 1172, 1030, 827, 752 cm^{-1}. Anal. Calcd for $C_{24}H_{20}N_2O_3$: C, 74.98; H, 5.24; N, 7.29. Found: C, 74.74; H, 5.01, N, 7.58.

*2-(2'-Hydroxyphenyl)-4,6-bis(4'-trifluoromethylphenyl)pyrimidine (**6b**).*

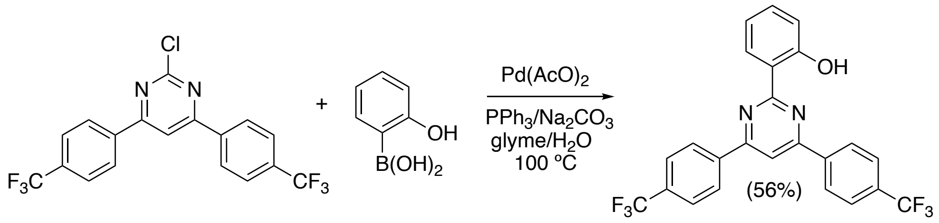

This compound was prepared from 2-chloro-4,6-(4′-trifluoromethylphenyl)pyrimidine (300 mg, 0.75 mmol), 2-hydroxyphenylboronic acid (114 mg, 0.83 mmol) following the same procedure described above for **6a**. The crude product was washed with boiling methanol to give a pale-yellow solid (190 mg, 56%). Further purification was achieved by crystallization from EtAcO/MeOH. Mp: 193-195 ºC. ^{1}H NMR ($CDCl_3$, 500 MHz) δ: 7.02 (m, 1H, ArH), 7.05 (dd, 1H, *J* = 8.5 Hz, *J* = 1.0 Hz, ArH), 7.44 (m, 1H, ArH), 7.83 (A of AB_q, 4H, *J* = 8.5 Hz, ArH), 7.96 (s, 1H, pyr), 8.25 (B of AB_q, 4H, *J* = 8.5 Hz, ArH), 8.66 (dd, 1H, *J* = 8.0 Hz, *J* = 1.5 Hz, ArH), 13.23 (broad s, 1H, OH). ^{13}C NMR and DEPT ($CDCl_3$, 125 MHz) δ: 165.5 (C), 163.1 (C), 160.8 (C), 139.5 (C), 133.8 (CH), 133.2 (q, *J* = 32.6 Hz, C), 129.6 (CH), 127.7 (CH), 126.2 (q, *J* = 3.6 Hz, CH), 123.7 (q, *J* = 270.8 Hz, CF_3), 119.3 (CH), 118.7 (C), 118.0 (CH), 110.8 (CH). ^{19}F NMR ($CDCl_3$, 471 MHz) δ: –62.9. MALDI-TOF MS (DHB) *m/z*: 461.2 [M+H]$^+$. IR (ATR) ν: 1594, 1570, 1530, 1321, 1112, 1067, 1013, 841, 758 cm^{-1}. Anal. Calcd for $C_{24}H_{14}F_6N_2O$: C, 62.61; H, 3.07; N, 6.08. Found: C, 62.97; H, 2.91, N, 6.30.

*4,6-Bis(4'-methoxyphenyl)-2-phenylpyrimidine (**6c**).*[24]

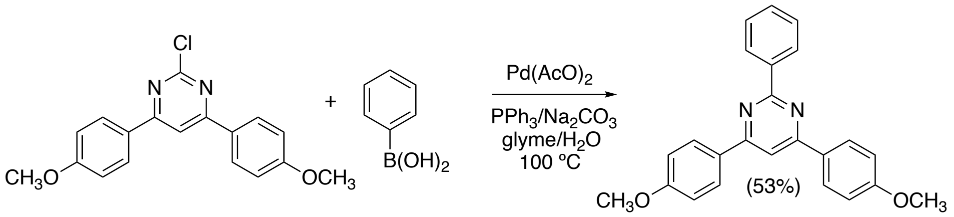

This compound was prepared from 2-chloro-4,6-(4′-methoxyphenyl)pyrimidine (100 mg, 0.31 mmol) and phenylboronic acid (42 mg, 0.34 mmol) following the same procedure described above for **6a**. The crude product was washed with boiling hexanes to give a pale-yellow solid (60 mg, 53%). Mp: 164-166 ºC (EtAcO/hexanes). ^{1}H NMR ($CDCl_3$, 500 MHz) δ: 3.92 (s, 6H, 2×OCH_3), 7.08 (A of AB_q, 4H, *J* = 9.0 Hz, ArH), 7.51-7.57 (m, 3H, ArH), 7.90 (s, 1H, pyr), 8.28 (B of AB_q, 4H, *J* = 9.0 Hz, ArH), 8.70-8.73 (m, 2H, ArH). ^{13}C NMR and DEPT ($CDCl_3$, 125 MHz) δ: 164.2 (C), 163.9 (C), 161.8 (C), 138.4 (C), 130.4 (CH), 130.1 (C), 128.7 (CH), 128.4 (CH), 128.3 (CH), 114.2 (CH), 108.5 (CH), 55.4 (CH_3). MALDI-TOF MS (dithranol) *m/z*: 369.3 [M+H]$^+$.

[24] (a) Bagley, M. C.; Lin, Z.; Pope, J. A. *Tetrahedron Lett.* **2009**, *50*, 6818-6822. (b) Deibl, N.; Kempe, R. *Angew. Chem. Int. Ed.* **2017**, *56*, 1663-1666. (c) Shen, J.; Meng, X. *Catal. Commun.* **2020**, *138*, 105846.

*2,4,6-Tris(4′-methoxyphenyl)pyrimidine (**6d**).*[22a,25]

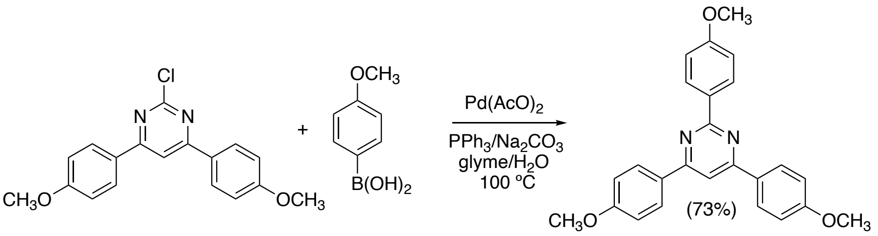

This compound was prepared from 2-chloro-4,6-(4′-methoxyphenyl)pyrimidine (100 mg, 0.31 mmol) and 4-methoxyphenylboronic acid (50 mg, 0.33 mmol) following the same procedure described above for **6a**. The crude product was purified by column chromatography (alumina, hexanes/EtAcO, 9:1) to give a colorless solid (90 mg, 73%). Mp: 171.2-173.1 ºC. ^{1}H NMR ($CDCl_3$, 500 MHz) δ: 3.91 (s, 6H, 2×OCH_3), 3.92 (s, 3H, OCH_3), 7.05 (A of AB_q, 2H, J = 9.0 Hz, ArH), 7.07 (A of AB_q, 4H, J = 9.0 Hz, Ar), 7.84 (s, 1H, pyr), 8.26 (B of AB_q, 4H, J = 9.0 Hz, ArH), 8.67 (B of AB_q, 2H, J = 9.0 Hz, ArH). ^{13}C NMR and DEPT ($CDCl_3$, 125 MHz) δ: 164.0 (C), 163.8 (C), 161.7 (C), 161.6 (C), 131.2 (C), 130.3 (C), 130.0 (CH), 128.7 (CH), 114.2 (CH), 113.7 (CH), 107.9 (CH), 55.4 (CH_3), 55.3 (CH_3). MALDI-TOF MS (dithranol) *m/z*: 399.3 $[M+H]^+$.

*2-(4′-Hydroxyphenyl)-4,6-bis(4′-methoxyphenyl)pyrimidine (**6e**).*

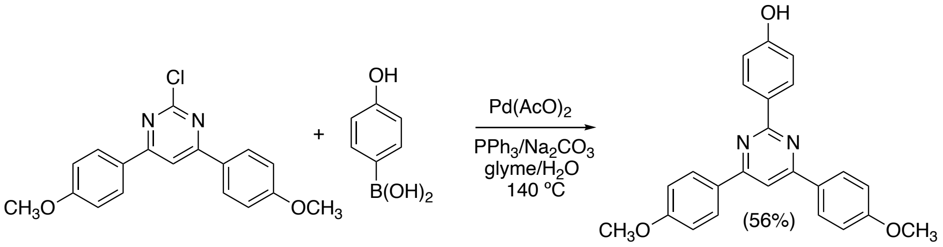

This compound was prepared from 2-chloro-4,6-(4′-methoxyphenyl)pyrimidine (150 mg, 0.46 mmol) and 4-hydroxyphenylboronic acid (70 mg, 0.51 mmol) following a similar procedure to that described above for **6a**. Purification was performed by filtration of an acetone solution through a short pad of Celite and alumina. Finally, the solvent was evaporated and the crude product washed a boiling mixture of EtAcO/hexanes (1:1) to give a colorless solid (100 mg, 56%). ^{1}H NMR (DMSO-d_6, 500 MHz) δ: 3.87 (s, 6H, 2×OCH_3), 6.95 (A of AB_q, 2H, J = 8.5 Hz, ArH), 7.13 (A of AB_q, 4H, J = 8.5 Hz, ArH), 8.27 (s, 1H, pyr), 8.43 (B of AB_q, 4H, J = 8.5 Hz, ArH), 8.48 (B of AB_q, 2H, J = 8.5 Hz, ArH), 9.95 (br s, 1H, OH). ^{13}C NMR and DEPT (DMSO-d_6, 125 MHz) δ: 163.2 (C), 163.2 (C), 161.6 (C), 160.0 (C), 129.7 (CH), 129.2 (C), 128.9 (CH), 128.7 (C), 115.3 (CH), 114.2 (CH), 107.5 (CH), 55.4 (CH_3). MALDI-TOF MS (dithranol) *m/z*: 385.3 $[M+H]^+$. IR (ATR) ν: 3131 (broad, OH), 1604, 1566, 1506, 1256, 1237, 1169, 829, 575, 520 cm^{-1}. Anal. Calcd for $C_{24}H_{20}N_2O_3$: C, 74.98; H, 5.24; N, 7.29. Found: C, 74.79; H, 5.13, N, 7.35.

---

[25] (a) Isfahani, A. L.; Mohammadpoor-Baltork, I.; Mirkhani, V.; Khosropour, A. R.; Moghadam, M.; Tangestaninejad, S.; Kia, R. *Adv. Synth. Catal.* **2013**, *355*, 957-972. (b) Rezaei, S.; Landarani-Isfahani, A.; Moghadam, M.; Tangestaninejad, S.; Mirkhani, V.; Mohammadpoor-Baltork, I. *RSC Adv.* **2016**, *6*, 92463-92472.

Table S1. Values of relative energies, in Kcal/mol, for the conformers of compounds **4a**, **4d**, **4e**, **6a**, **6c, 6d**, and **6e** in CH_2Cl_2, calculated at the M06-2X/6-31+G** level of theory. *K* and *E* mean keto and enol forms, respectively.

Compound	State	E (hartree)	ΔE (kcal/mol)
**4a**	S_0	-1416.092314	
	S_1 (*E*)	-1415.977475	4.32
	S_1 (*K*)	-1415.984361	0.00
**4d**	S_0	-1340.878748	
**4e**	S_0	-1455.364253	
**6a**	S_0	-1261.347544	
	S_1 (*E*)	-1261.206975	12.64
	S_1 (*K*)	-1261.227121	0.00
**6c**	S_0	-1186.133950	
**6d**	S_0	-1300.619595	
**6e**	S_0	-1261.340013	
	S_1	-1261.196334	

Table S2. Maximum absorption wavelengths (λ_{ab}^{max}), calculated transition wavelengths ($\lambda_{vert\text{-}ab}^{calc}$), and oscillator strengths (*f*) for $S_0 \rightarrow S_n$ transitions. Calculations were carried out at the M06-2X/6-31+G** level of theory in CH_2Cl_2 solution.

Compd	λ_{ab}^{max} eV(nm)	$\lambda_{vert\text{-}ab}^{calc}$ eV(nm)	Transition	*f*	% Contr. (≥10%)
**4a**	3.23 (384)	3.45 (360)	$S_0 \rightarrow S_1$	1.86	H → L (90)
		3.84 (323)	$S_0 \rightarrow S_2$	0.30	H-1 → L (84)
		4.16 (298)	$S_0 \rightarrow S_3$	0.15	H-1 → L+1 (82)
**4d**	3.34 (371)	3.55 (350)	$S_0 \rightarrow S_1$	1.89	H → L (89)
		3.91 (317)	$S_0 \rightarrow S_2$	0.24	H-1 → L (86)
		4.46 (278)	$S_0 \rightarrow S_4$	0.35	H-1 → L+1 (50), H-2 → L (36)
**4e**	3.32 (373)	3.54 (350)	$S_0 \rightarrow S_1$	1.78	H → L (89)
		3.91 (317)	$S_0 \rightarrow S_2$	0.30	H-1 → L (84)
		4. 26 (291)	$S_0 \rightarrow S_4$	0.34	H-2 → L (74), H-1 → L +1 (16)
**6a**	3.76 (330)	4.17 (297)	$S_0 \rightarrow S_1$	0.80	H → L (79), H-1 → L (12)
		4.31 (288)	$S_0 \rightarrow S_2$	0.46	H → L+1 (55), H-1 → L+1 (27)
		4. 06 (269)	$S_0 \rightarrow S_4$	0.26	H-1 → L (41), H-2 → L (15), H-6 → L (11)
**6c**	3.81 (325)	4.28 (290)	$S_0 \rightarrow S_1$	0.90	H → L (91)
		4.67 (265)	$S_0 \rightarrow S_4$	0.37	H-1 → L (82)
		4.74 (262)	$S_0 \rightarrow S_5$	0.17	H → L+1 (76), H-2 → L+1 (11)
**6d**	3.72 (333)	4.22 (294)	$S_0 \rightarrow S_1$	0.67	H → L (87)
		4.53 (273)	$S_0 \rightarrow S_3$	0.90	H → L+1 (60), H-2 → L+1 (16), H-1 → L (14)
		4.68 (265)	$S_0 \rightarrow S_4$	0.15	H-6 → L+1 (41), H-4→L+1 (28), H-1 → L+1 (12)
**6e**	3.75 (331)	4.23 (293)	$S_0 \rightarrow S_1$	0.70	H → L (89)
		4.58 (271)	$S_0 \rightarrow S_3$	0.83	H → L+1 (50), H-1 → L (21), H-2 → L+1 (20)
		4.69 (265)	$S_0 \rightarrow S_4$	0.13	H-6 → L+1 (44), H-4→L+1 (27), H-1 → L+1 (11)

Table S3. Wavenumber (ν_i in cm^{-1}), reorganization energy (λ_i in meV), and Huang-Rhys factors (adimensional) calculated for the studied compounds in CH_2Cl_2 solution at the M06-2X/6-31+G** level of the theory.

**4a**			**4d**			**4e**			**6a**			**6c**			**6d**			**6e**		
ν_i	λ_i	HR	ν_i	λ_i	HR	ν_i	λ_i	HR	ν_i	λ_i	HR	ν_i	λ_i	HR	ν_i	λ_i	HR	ν_i	λ_i	HR
107	1	0.08	25	2	0.64	24	2	0.67	24	9	3.02	24	16	5.37	21	16	6.14	21	1	0.38
149	2	0.11	103	1	0.08	132	4	0.24	31	9	2.34	38	26	5.51	28	2	0.58	23	14	4.91
327	5	0.12	136	4	0.24	166	2	0.10	34	25	5.93	46	11	1.93	30	4	1.07	35	24	5.53
355	4	0.09	198	4	0.16	252	1	0.03	39	2	0.41	86	6	0.56	35	5	1.15	45	10	1.79
444	1	0.02	352	1	0.02	541	1	0.01	71	4	0.45	107	10	0.75	44	2	0.37	76	15	1.59
503	31	0.50	540	1	0.01	550	2	0.03	157	2	0.10	176	12	0.55	45	5	0.90	98	3	0.25
551	1	0.01	550	2	0.03	670	2	0.02	184	20	0.88	230	6	0.21	63	1	0.13	179	12	0.54
579	14	0.19	667	3	0.04	878	2	0.02	191	3	0.13	238	1	0.03	65	13	1.61	186	6	0.26
648	1	0.01	877	2	0.02	885	1	0.01	207	9	0.35	320	1	0.03	93	1	0.09	226	2	0.07
670	14	0.17	885	1	0.01	1023	1	0.01	229	10	0.35	354	1	0.02	109	4	0.30	324	1	0.02
711	1	0.01	1015	1	0.01	1023	10	0.08	244	5	0.17	416	3	0.06	170	2	0.09	408	5	0.10
849	6	0.06	1024	11	0.09	1026	1	0.01	261	1	0.03	428	1	0.02	177	6	0.27	427	1	0.02
945	1	0.01	1026	1	0.01	1028	1	0.01	283	2	0.06	503	1	0.02	186	5	0.22	500	1	0.02
1042	1	0.01	1175	2	0.01	1172	2	0.01	319	6	0.15	600	5	0.07	212	1	0.04	594	4	0.05
1129	1	0.01	1180	3	0.02	1178	4	0.03	336	1	0.02	680	1	0.01	332	1	0.02	613	1	0.01
1165	12	0.08	1201	5	0.03	1201	2	0.01	354	3	0.07	785	1	0.01	400	4	0.08	675	1	0.01
1181	2	0.01	1202	8	0.05	1201	7	0.05	404	2	0.04	814	5	0.05	421	1	0.02	754	1	0.01
1190	2	0.01	1213	1	0.01	1202	3	0.02	413	1	0.02	843	1	0.01	427	1	0.02	805	3	0.03
1246	3	0.02	1235	1	0.01	1212	1	0.01	458	2	0.04	912	1	0.01	504	1	0.02	820	1	0.01
1260	1	0.01	1243	1	0.01	1235	1	0.01	486	14	0.23	985	1	0.01	594	2	0.03	840	1	0.01
1271	2	0.01	1269	13	0.08	1243	1	0.01	495	1	0.02	1014	1	0.01	601	1	0.01	843	1	0.01
1282	8	0.05	1311	2	0.01	1269	13	0.08	502	1	0.02	1028	30	0.24	614	1	0.01	909	1	0.01
1297	21	0.13	1328	2	0.01	1311	2	0.01	522	3	0.05	1056	2	0.02	681	1	0.01	985	1	0.01
1313	1	0.01	1350	1	0.01	1328	2	0.01	537	1	0.02	1101	1	0.01	795	2	0.02	1026	16	0.13
1320	1	0.01	1351	12	0.07	1349	16	0.10	590	3	0.04	1182	2	0.01	827	3	0.03	1029	15	0.12
1327	2	0.01	1353	4	0.02	1351	1	0.01	601	2	0.03	1200	4	0.03	908	1	0.01	1101	1	0.01
1329	5	0.03	1374	2	0.01	1373	2	0.01	604	14	0.19	1205	1	0.01	1026	13	0.10	1175	1	0.01
1340	2	0.01	1429	3	0.02	1426	1	0.01	642	1	0.01	1215	1	0.01	1028	16	0.13	1199	3	0.02
1351	8	0.05	1473	1	0.01	1430	2	0.01	661	8	0.10	1314	4	0.02	1075	1	0.01	1215	1	0.01
1352	33	0.20	1473	6	0.03	1472	1	0.01	679	2	0.02	1423	18	0.10	1180	1	0.01	1312	1	0.01
1354	1	0.01	1513	1	0.01	1473	6	0.03	757	1	0.01	1423	34	0.19	1196	1	0.01	1313	1	0.01
1376	1	0.01	1605	54	0.27	1502	1	0.01	765	1	0.01	1468	2	0.01	1197	3	0.02	1314	1	0.01
1424	2	0.01	1635	6	0.03	1567	1	0.01	788	2	0.02	1487	2	0.01	1312	1	0.01	1421	36	0.20
1430	91	0.51	1648	3	0.01	1605	52	0.26	808	3	0.03	1575	20	0.10	1313	1	0.01	1426	16	0.09
1458	91	0.50	1650	2	0.01	1632	4	0.02	819	24	0.24	1612	19	0.09	1421	34	0.19	1467	1	0.01
1473	1	0.01	1667	1	0.00	1648	3	0.01	840	4	0.04	1659	4	0.02	1425	19	0.11	1487	2	0.01
1474	5	0.03	1683	1	0.00	1650	2	0.01	841	8	0.08	1682	1	0.00	1466	1	0.01	1568	1	0.01
1487	1	0.01	1687	19	0.09	1660	2	0.01	842	9	0.09	1689	35	0.17	1486	1	0.01	1570	1	0.01
1531	33	0.17	1688	11	0.05	1686	14	0.07	855	9	0.08				1486	1	0.01	1575	19	0.10
1555	37	0.19	1723	26	0.12	1687	15	0.07	860	14	0.13				1570	3	0.02	1611	14	0.07
1567	1	0.01	1736	63	0.29	1688	2	0.01	864	2	0.02				1575	18	0.09	1659	2	0.01
1602	84	0.42				1723	27	0.13	881	4	0.04				1611	14	0.07	1672	1	0.00
1636	14	0.07				1736	62	0.29	890	4	0.04				1659	3	0.01	1687	1	0.00
1666	1	0.28							907	1	0.01				1686	1	0.00	1688	18	0.09
1686	1	0.01							1020	2	0.02				1687	19	0.09	1690	13	0.06
1699	57	0.09							1075	2	0.01				1690	12	0.06			

Table S3 (continued).

4a			4d			4e			6a			6c			6d			6e		
v_i	λ_i	HR	v_i	λ_i	HR	v_i	λ_i	HR	v_i	λ_i	HR	v_i	λ_i	HR	v_i	λ_i	HR	v_i	λ_i	HR
3131	2	28.44							1138	1	0.01									
3202	20	0.02							1167	11	0.08									
3211	1	0.01							1196	1	0.01									
3212	1	0.01							1198	2	0.01									
3218	11046	0.02							1202	2	0.01									
3240	6	0.01							1255	9	0.06									
									1278	1	0.01									
									1293	15	0.09									
									1315	1	0.01									
									1347	6	0.04									
									1352	11	0.07									
									1356	4	0.02									
									1424	88	0.50									
									1441	7	0.04									
									1483	3	0.02									
									1486	1	0.01									
									1488	11	0.06									
									1511	1	0.01									
									1529	28	0.15									
									1545	3	0.02									
									1576	17	0.09									
									1610	44	0.22									
									1639	38	0.19									
									1654	30	0.15									
									1659	34	0.17									
									1668	31	0.15									
									1686	1	0.00									
									1689	3	0.01									
									3062	1	0.00									
									3176	562	1.43									
									3218	2	0.01									

Table S4. Calculated transition wavelengths ($\lambda_{vert\text{-}em}^{calc}$) and oscillator strengths (*f*) in the crystal for the $S_1 \rightarrow S_0$ transitions of the enol (E) and keto (K) forms for the central molecule of **4a** and the dimer of **6e** at the M06-2X/6-31G** level of theory.

Compound	$\lambda_{vert\text{-}em}^{calc}$ eV (nm)	*f*
**4a**	2.98 (417) E	1.75
	1.19 (1045) K	0.008
**6e dimer**	3.98 (312) E	0.40
	1.13 (1094) K	0.03

Table S5. Values of relative energies, in kcal/mol, for protonated **4a** and **6a** in CH_2Cl_2, calculated at the M06-2X/6-31+G** level of theory. K and E mean keto and enol forms, respectively.

Compound	State	E (hartree)	ΔE (kcal/mol)
**4aH$^+$-(*1*)**	S_0	-1416.519037	1.12
	S_1 (E)	-1416.428386	0.00
	S_1 (K)	-1416.415472	8.10
**4aH$^+$-(*2*)**	S_0	-1416.520823	0.00
	S_1	-1416.424385	
**6aH$^+$-(*1*)**	S_0	-1261.772180	1.25
	S_1 (E)	-1261.656001	1.62
	S_1 (K)	-1261.658585	0.00
**6aH$^+$-(*2*)**	S_0	-1261.774173	0.00
	S_1	-1261.652118	

Table S6. Maximum absorption wavelengths (λ_{ab}^{max}) determined for protonated compounds **4a** and **6a.** Calculated transition wavelengths ($\lambda_{vert\text{-}ab}^{calc}$) and oscillator strengths (*f*) for $S_0 \rightarrow S_n$ transitions. Calculations were carried out at the M06-2X/6-31+G** level of theory in CH_2Cl_2 solution.

Compd	λ_{ab}^{max} eV(nm)	$\lambda_{vert\text{-}ab}^{calc}$ eV (nm)	Transition	*f*	% Contr. (≥10%)
**4aH$^+$-(*1*)**	2.65 (468)	2.76 (450)	$S_0 \rightarrow S_1$	2.06	H → L (92)
	3.04 (408)	3.29 (379)	$S_0 \rightarrow S_2$	0.33	H-1 → L (83)
**4aH$^+$-(*2*)**		2.92 (425)	$S_0 \rightarrow S_1$	2.04	H → L (91)
		3.42 (363)	$S_0 \rightarrow S_3$	0.29	H → L+1 (87)
		4.15 (299)	$S_0 \rightarrow S_4$	0.12	H-2 → L (43), H-1→L+1 (20), H → L+1 (19)
**6aH$^+$-(*1*)**	3.14 (395)	3.46 (359)	$S_0 \rightarrow S_1$	1.09	H → L (90)
	3.45 (359)	3.67 (337)	$S_0 \rightarrow S_2$	0.45	H-1 → L (79), H-1 → L+1(10)
**6aH$^+$-(*2*)**		3.62 (342	$S_0 \rightarrow S_1$	1.12	H → L (89)
		3.95 (314)	$S_0 \rightarrow S_2$	0.54	H-1 → L (80)

Table S7. Wavenumber (ν_i in cm^{-1}), reorganization energy (λ_i in meV) and Huang-Rhys factors (adimensional) calculated for protonated **4a** and **6a** in CH_2Cl_2 solution at the M06-2X/6-31+G** level of theory.

**4aH⁺-(*1*)**			**4aH⁺-(*2*)**			**6aH⁺-(*1*)**			**6aH⁺-(*2*)**		
ν_i	λ_i	HR	ν_i	λ_i	HR	ν_i	λ_i	HR	ν_i	λ_i	HR
23	3	1.05	25	1	0.32	28	5	1.44	26	3	0.93
135	1	0.06	134	1	0.06	42	1	0.19	38	1	0.21
261	1	0.03	274	1	0.03	49	5	0.82	41	11	2.16
275	1	0.03	331	2	0.05	53	1	0.15	45	13	2.33
464	1	0.02	346	1	0.02	84	5	0.48	89	8	0.72
500	2	0.03	464	2	0.03	102	7	0.55	105	2	0.15
549	2	0.03	550	2	0.03	106	5	0.38	121	1	0.07
555	1	0.01	881	2	0.02	154	1	0.05	161	2	0.10
674	1	0.01	1017	7	0.06	185	17	0.74	174	2	0.09
709	4	0.05	1024	2	0.02	208	1	0.04	179	6	0.27
717	2	0.02	1037	3	0.02	246	6	0.20	187	1	0.04
883	2	0.02	1173	3	0.02	250	3	0.10	201	2	0.08
944	1	0.01	1193	1	0.01	252	2	0.06	248	1	0.03
1007	2	0.02	1201	8	0.05	275	1	0.03	318	3	0.08
1014	1	0.01	1262	3	0.02	280	3	0.09	345	1	0.02
1021	2	0.02	1267	6	0.04	346	1	0.02	389	1	0.02
1025	3	0.02	1285	2	0.01	452	10	0.18	417	1	0.02
1026	2	0.02	1308	2	0.01	486	29	0.48	426	1	0.02
1040	1	0.01	1322	2	0.01	492	14	0.23	490	1	0.02
1170	1	0.01	1350	4	0.02	503	3	0.05	601	5	0.07
1199	10	0.07	1357	8	0.05	524	9	0.14	802	1	0.01
1243	3	0.02	1401	4	0.02	556	4	0.06	806	1	0.01
1254	2	0.01	1442	5	0.03	591	21	0.29	825	1	0.01
1263	2	0.01	1468	2	0.01	601	2	0.03	838	1	0.01
1287	4	0.03	1476	1	0.01	672	1	0.01	879	2	0.02
1326	2	0.01	1480	5	0.03	681	1	0.01	906	1	0.01
1328	1	0.01	1504	3	0.02	714	3	0.03	1023	4	0.03
1332	1	0.01	1522	4	0.02	742	4	0.04	1030	12	0.09
1346	1	0.01	1555	8	0.04	755	2	0.02	1126	1	0.01
1350	1	0.01	1615	26	0.13	764	3	0.03	1138	1	0.01
1355	11	0.07	1636	9	0.04	791	2	0.02	1176	3	0.02
1400	3	0.02	1651	4	0.02	841	1	0.01	1194	1	0.01
1416	1	0.01	1671	6	0.03	875	1	0.01	1208	3	0.02
1454	3	0.02	1682	8	0.04	880	4	0.04	1212	1	0.01
1470	2	0.01	1702	1	0.00	1017	2	0.02	1214	1	0.01
1475	6	0.03	1724	16	0.07	1030	1	0.01	1276	1	0.01
1478	1	0.01			0.01	1057	1	0.01	1281	2	0.01
1501	1	0.01			0.13	1140	1	0.01	1313	1	0.01
1528	3	0.02			0.01	1154	2	0.01	1324	4	0.02
1551	6	0.03			0.00	1171	20	0.14	1339	1	0.01
1607	15	0.08			0.05	1202	10	0.07	1356	2	0.01
1625	8	0.04			0.04	1291	12	0.07	1359	2	0.01
1661	9	0.04			0.01	1326	6	0.04	1362	1	0.01
1681	7	0.03			0.04	1339	1	0.01	1373	4	0.02
1720	10	0.05				1364	49	0.29	1430	22	0.12
						1376	9	0.05	1489	1	0.01
						1406	22	0.13	1558	1	0.01
						1442	41	0.23	1578	7	0.04
						1455	21	0.12	1631	15	0.07
						1468	8	0.04	1643	7	0.03
						1482	1	0.01	1648	6	0.03
						1487	2	0.01	1669	4	0.02
						1488	12	0.06	1686	10	0.05
						1504	15	0.08			
						1508	6	0.03			
						1509	7	0.04			
						1531	18	0.09			
						1555	27	0.14			
						1581	11	0.06			
						1621	11	0.05			
						1645	2	0.01			
						1657	10	0.05			
						1661	31	0.15			
						1685	5	0.02			
						1695	1	0.00			
						1700	66	0.31			
						3239	1	0.00			
						3474	12884	29.89			

Table S8. Bond critical points (BCP) properties for the hydrogen bonds (electron density φ_r; Laplacian of electron density $\nabla^2\varphi_r$; kinetic energy density G_r; potential energy density V_r; electronic energy density H_r; dissociation energy E_{dis}).

BCP Properties	4a	6a	4a cryst	4aH$^+$-(*1*)	6aH$^+$-(*1*)	4aH$^+$-(*2*)	6aH$^+$-(*2*)	6e cryst
H···O distance (Å)	1.663	1.681	1.707	1.774	1.794	1.809	1.824	2.024
φ_r (eÅ^{-3})	0.0584	0.0557	0.0506	0.0438	0.0417	0.0339	0.0330	0.0238
$\nabla^2\varphi_r$ (eÅ^{-5})	0.1261	0.1255	0.1507	0.1164	0.1138	0.1277	0.1244	0.0700
G_r (a.u.)	0.0403	0.0387	0.0408	0.0317	0.0305	0.0307	0.0299	0.0175
V_r (a.u.)	-0.0490	-0.0459	-0.0440	-0.0344	-0.0326	-0.0295	-0.0288	-0.0175
H_r (a.u.)	-0.0087	-0.0073	-0.0032	-0.0026	-0.0020	0.0012	0.0012	0.00001
E_{dis} (kJ/mol)	64.4	60.3	57.7	45.1	42.7	38.7	37.8	23.0

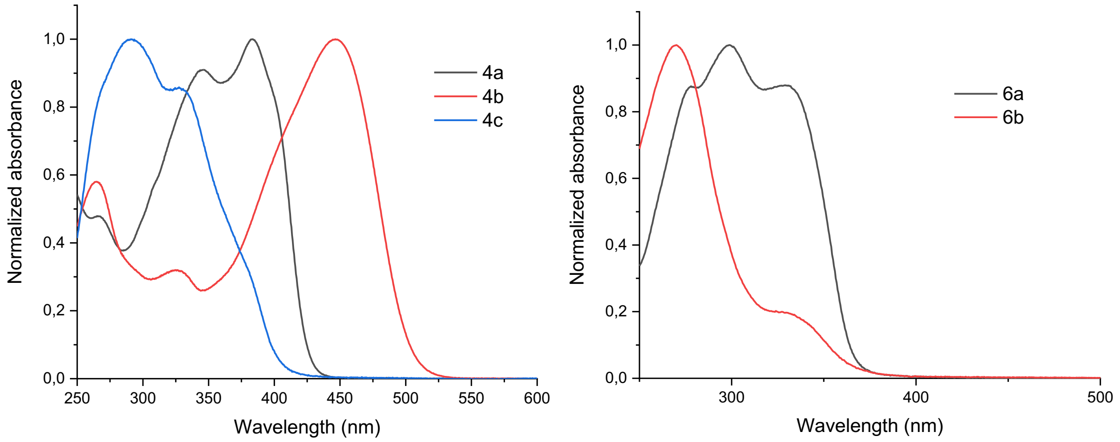

Figure S1. UV/vis spectra of **4a-c** (c = 2.77-4.12 × 10^{-6} M) and **6a-b** (c = 2.96-5.00 × 10^{-6} M) in CH_2Cl_2 solution.

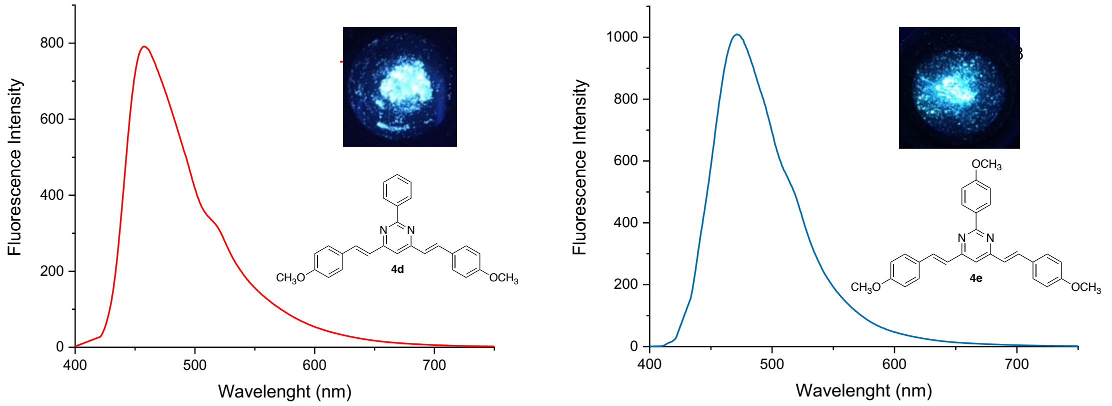

Figure S2. Emission spectra of **4d** (λ_{exc} = 413 nm) and **4e** (λ_{exc} = 426 nm) in the solid state. The pictures were taken in the dark upon irradiation with a UV hand-held lamp (λ_{exc} = 365 nm, 24 W).

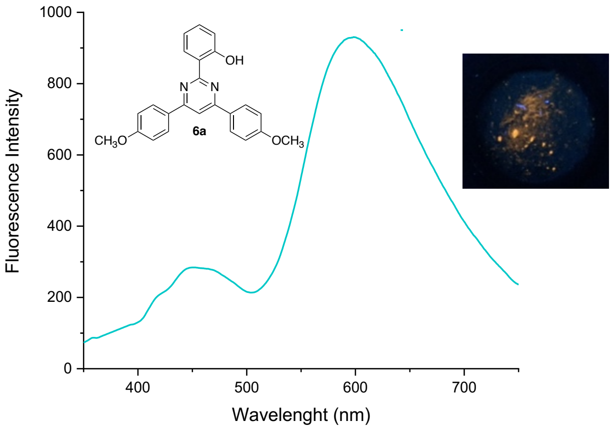

Figure S3. Emission spectra of **6a** (λ_{exc} = 378 nm) in the solid state. The pictures were taken in the dark upon irradiation with a UV hand-held lamp (λ_{exc} = 365 nm, 24 W).

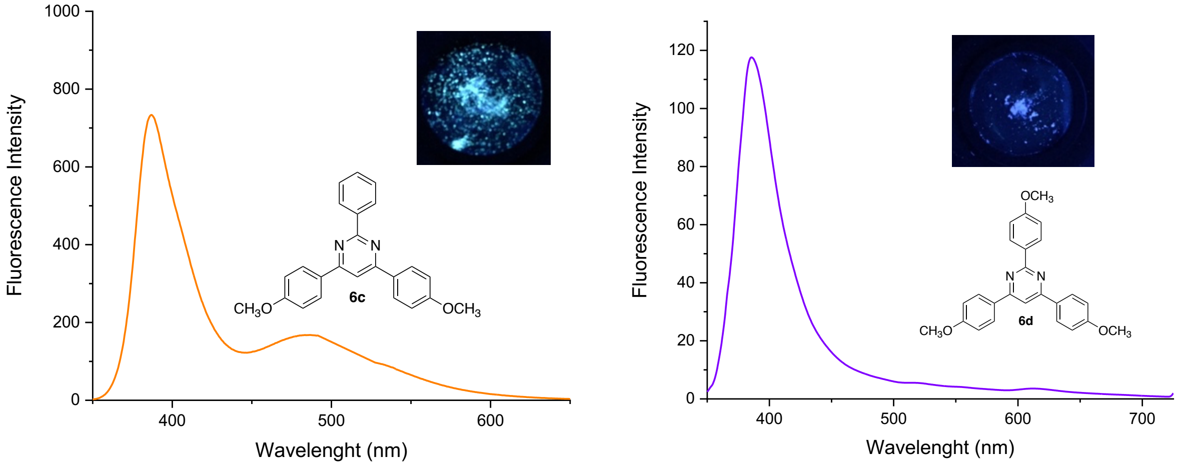

Figure S4. Emission spectra of **6c** (λ_{exc} = 330 nm) and **6d** (λ_{exc} = 365 nm) in the solid state. The pictures were taken in the dark upon irradiation with a UV hand-held lamp (λ_{exc} = 365 nm, 24 W).

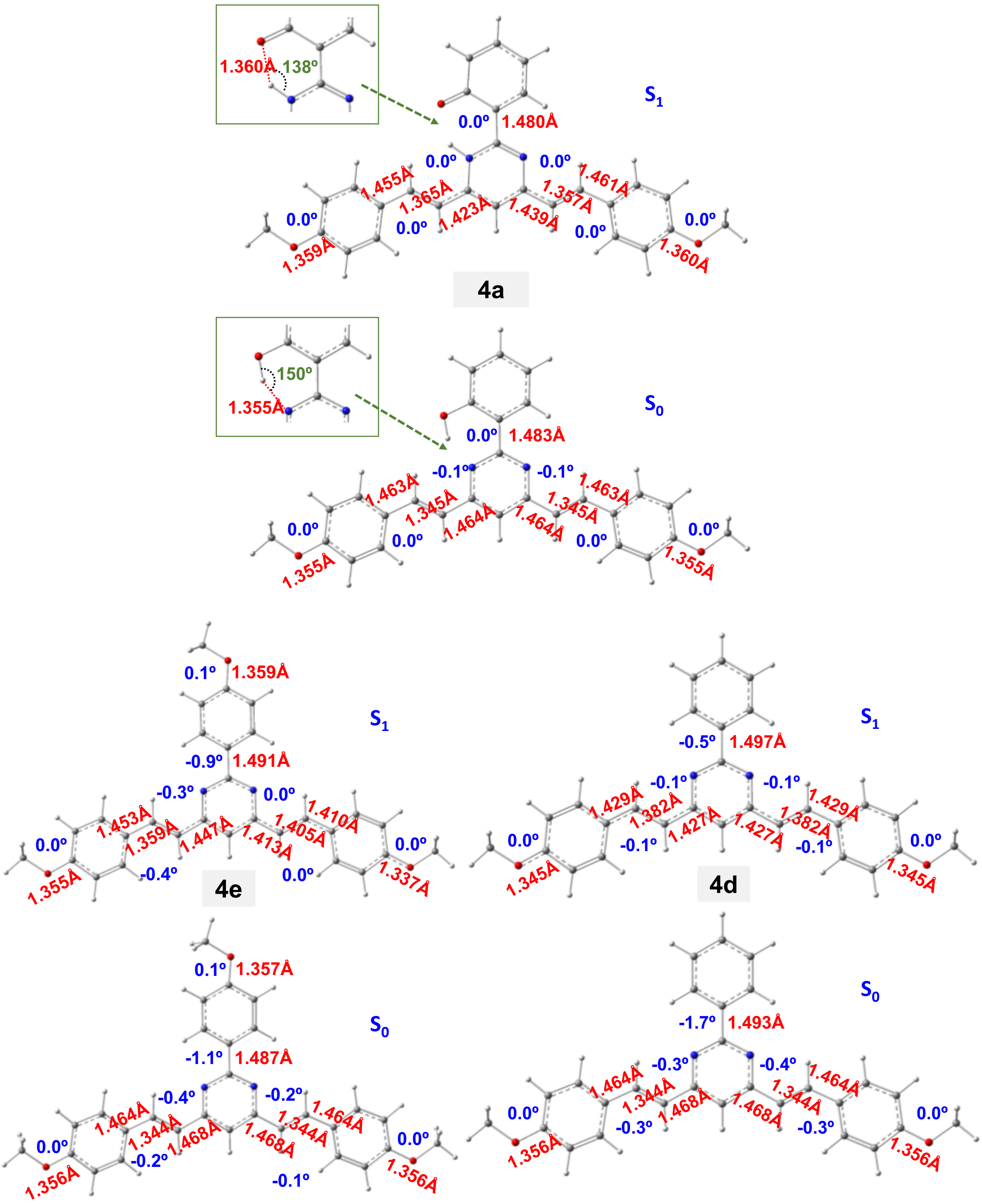

Figure S5. Selected bond lengths and dihedral angles in the S_0 and S_1 states for compounds **4a** and **4d-e** calculated in CH_2Cl_2 at the M06-2X/6-31+G** level of theory.

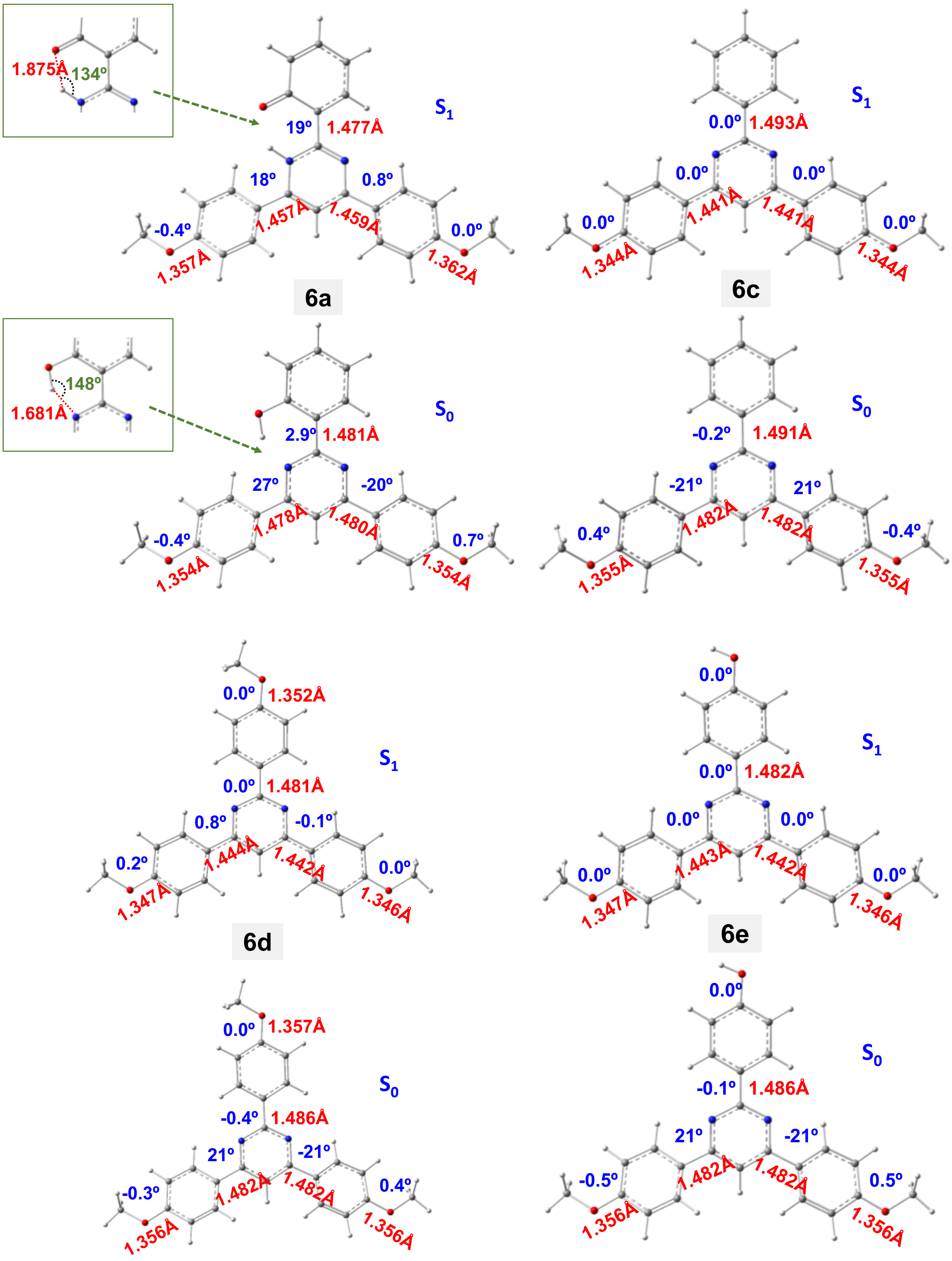

Figure S6. Selected bond lengths and dihedral angles in the S_0 and S_1 states for compounds **6a**, and **6c-e** calculated in CH_2Cl_2 at the M06-2X/6-31+G** level of theory.

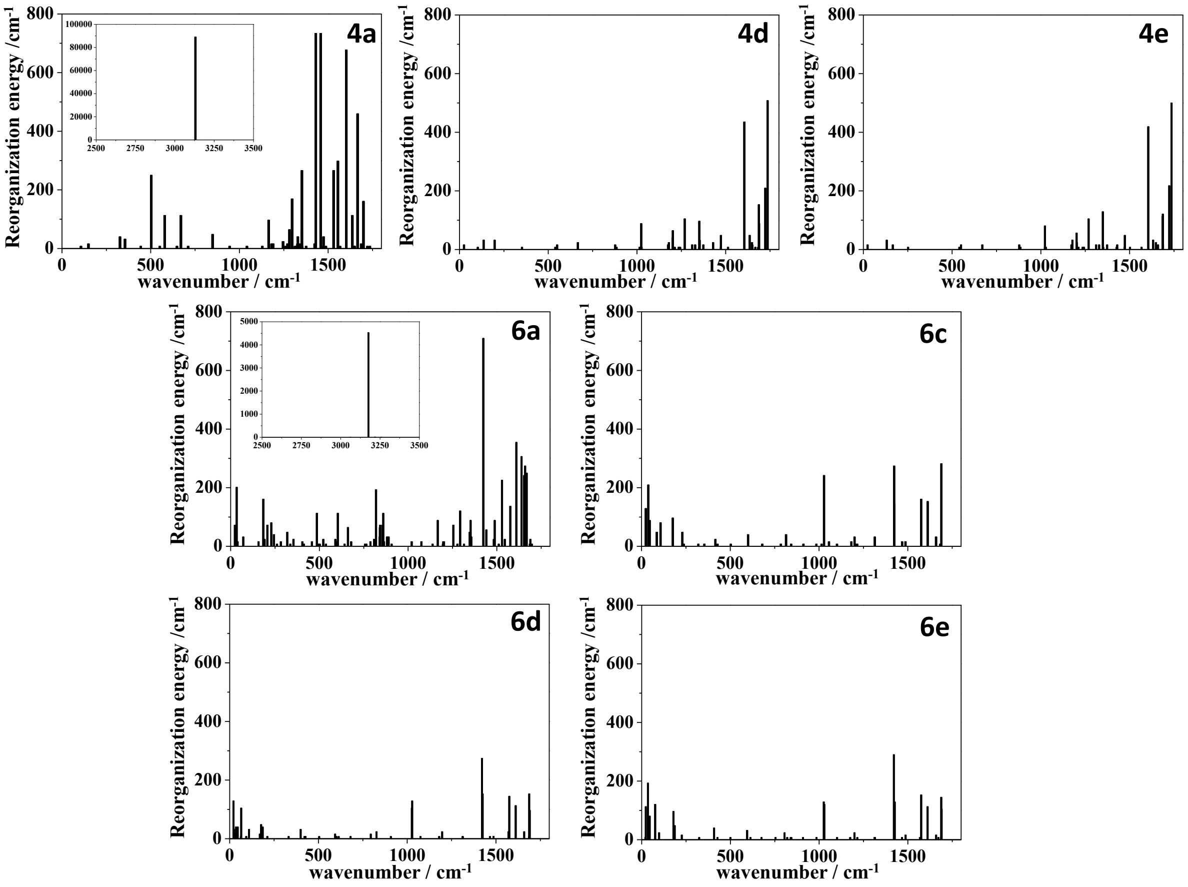

Figure S7. Reorganization energy (in cm^{-1}) *versus* normal mode wavenumbers (in cm^{-1}) calculated for the ground state of compounds **4a**, **4d-e**, **6a**, and **6c-e** in CH_2Cl_2 at the M06-2X/6-31+G** level of theory.

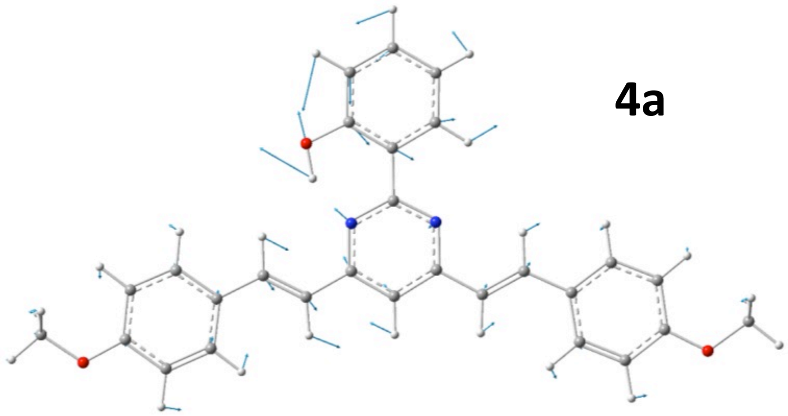

4a

$\boldsymbol{\omega_i}$ **= 24 cm^{-1},** $\boldsymbol{S_i}$ **= 3.02**

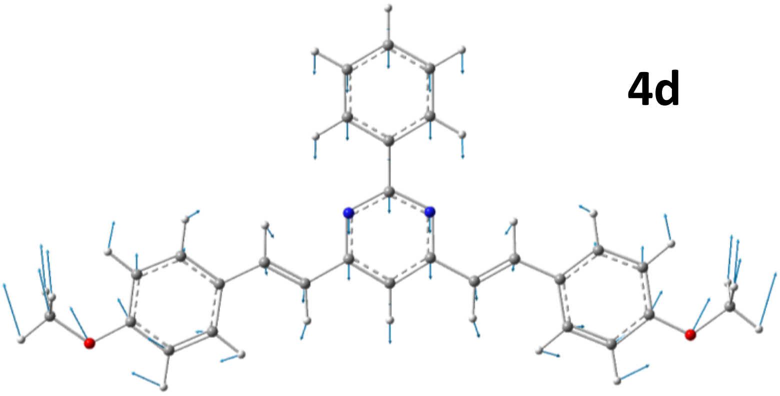

4d

$\boldsymbol{\omega_i}$ **= 25 cm^{-1},** $\boldsymbol{S_i}$ **= 0.64**

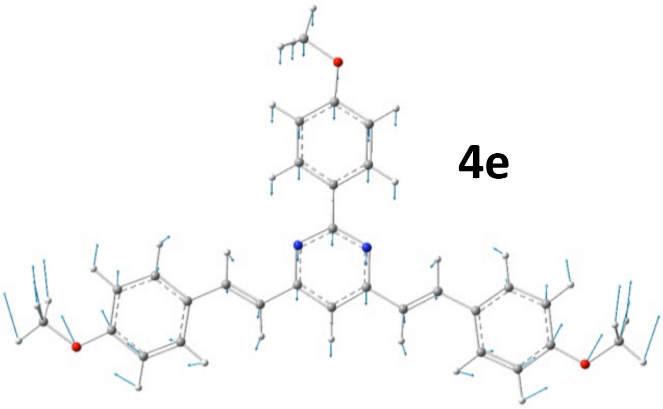

4e

$\boldsymbol{\omega_i}$ **= 24 cm^{-1},** $\boldsymbol{S_i}$ **= 0.67**

Figure S8. Atomic displacements of selected vibrational modes calculated for compounds **4a** and **4d-e** in CH_2Cl_2 solution at the M06-2X/6-31+G** level of theory.

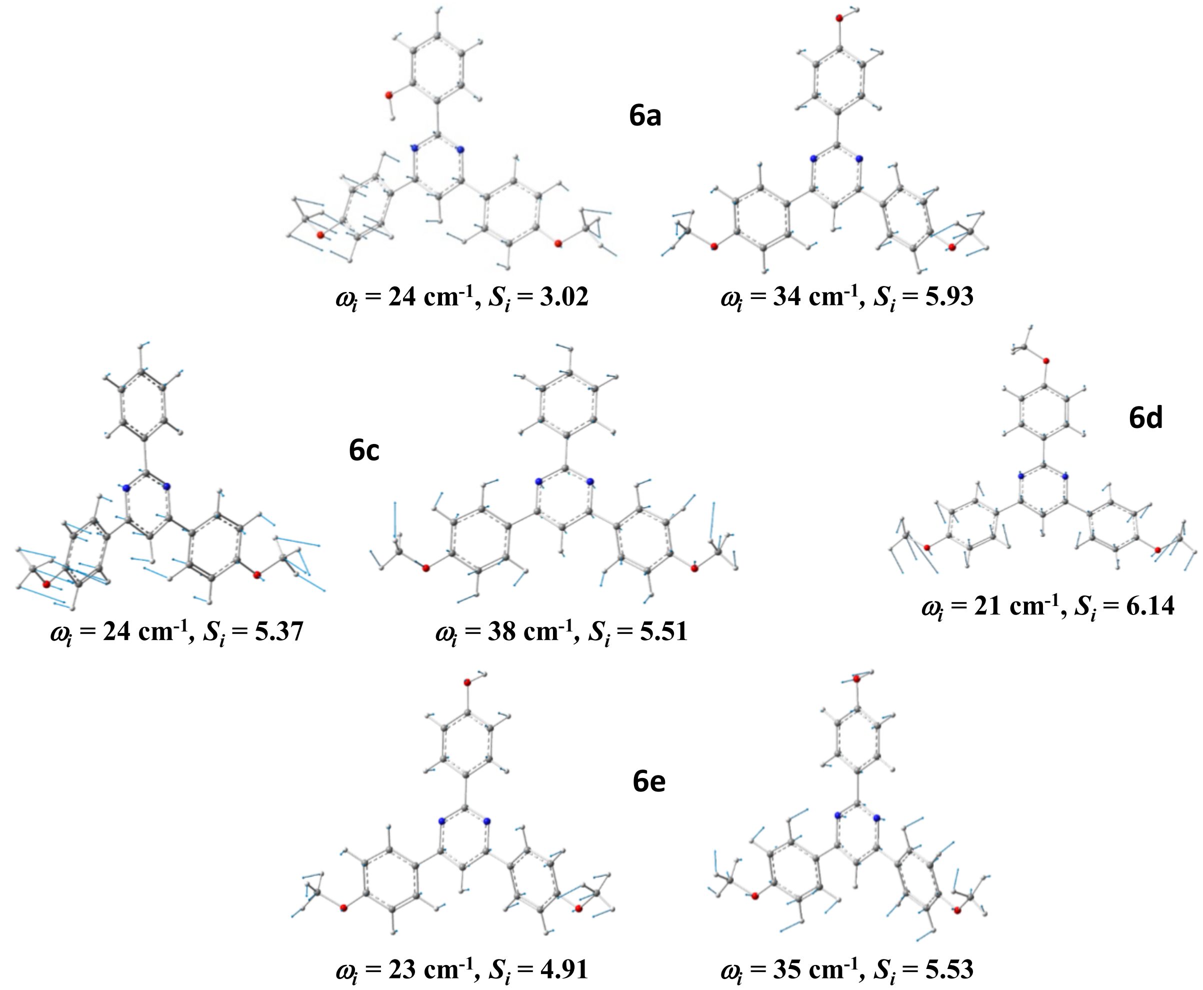

Figure S9. Atomic displacements of selected vibrational modes calculated for compounds **6a** and **6c-e** in CH_2Cl_2 solution at the M06-2X/6-31+G** level of theory.

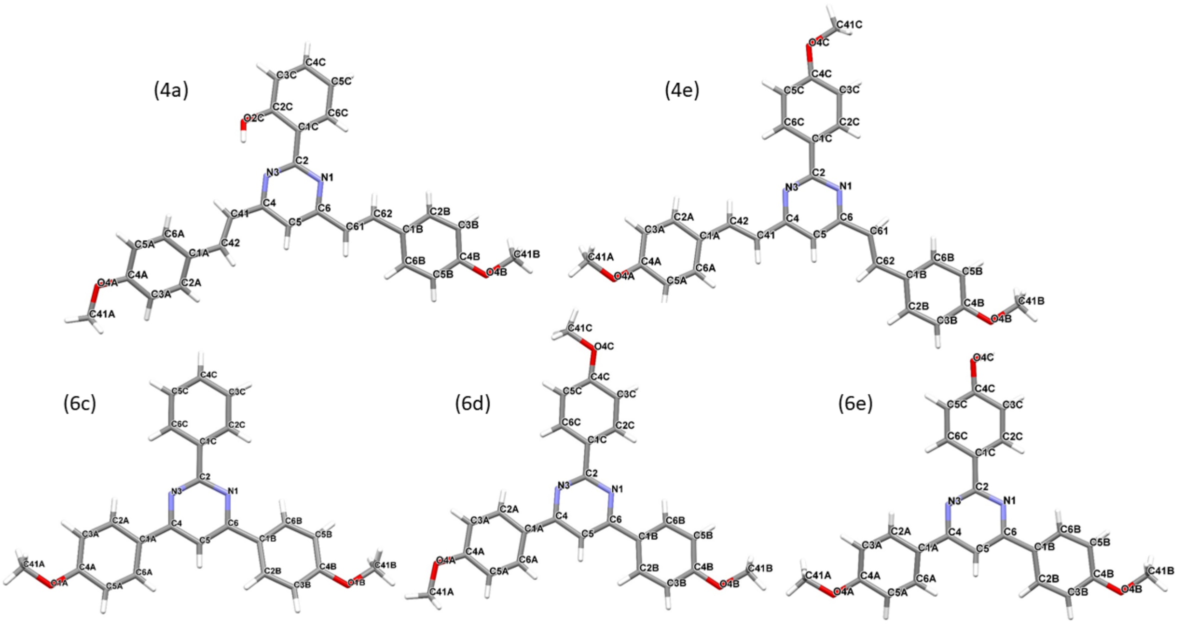

Figure S10. Molecular structure of compounds **4a**, **4e**, and **6c-e** extracted from X-ray analysis.

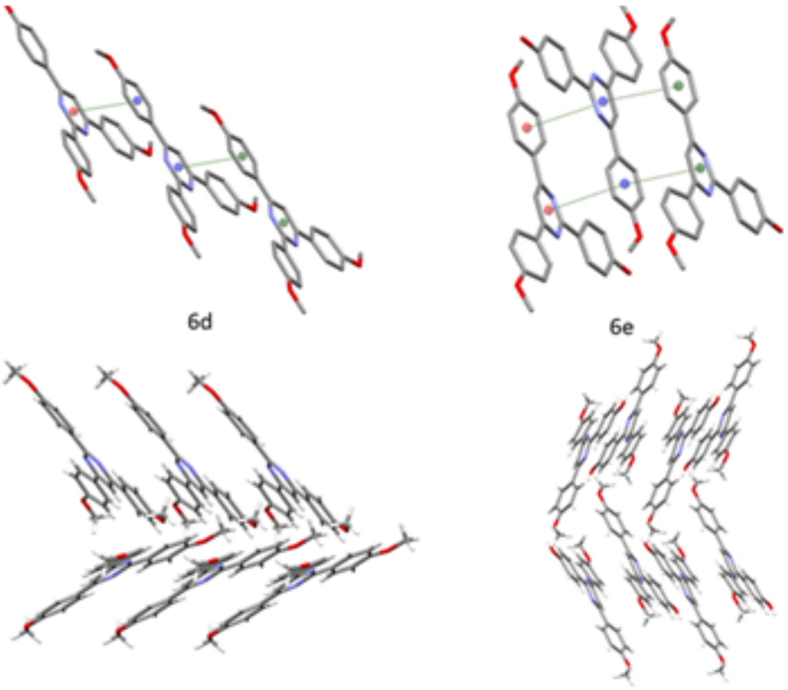

Figure S11. Pattern of the π-π interactions between molecules (top) and crystal packing (bottom) of compounds **6d** and **6e**. For clarity, neither hydrogen atoms (upper) nor atom labels are displayed.

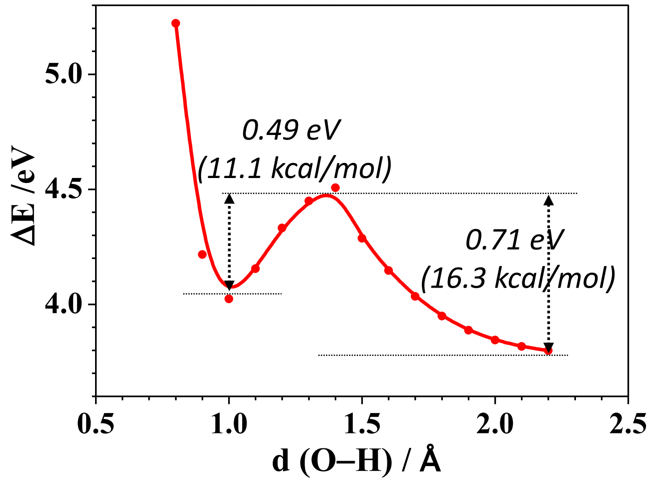

Figure S12. Potential energy surface of excited state S_1 for the dimer **6e** in CH_2Cl_2 solution calculated at the M06-2X/6-31G** level of theory.

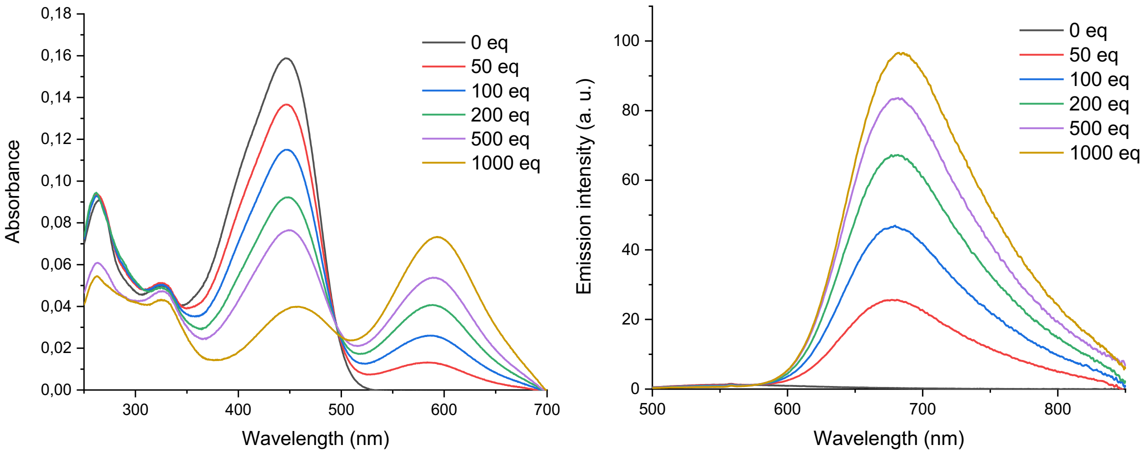

Figure S13. Changes in the absorption (left) and emission (right, λ_{exc} = 477 nm) spectra of a CH_2Cl_2 solution of **4b** ($c = 2.77 \times 10^{-6}$ M) upon addition of TFA.

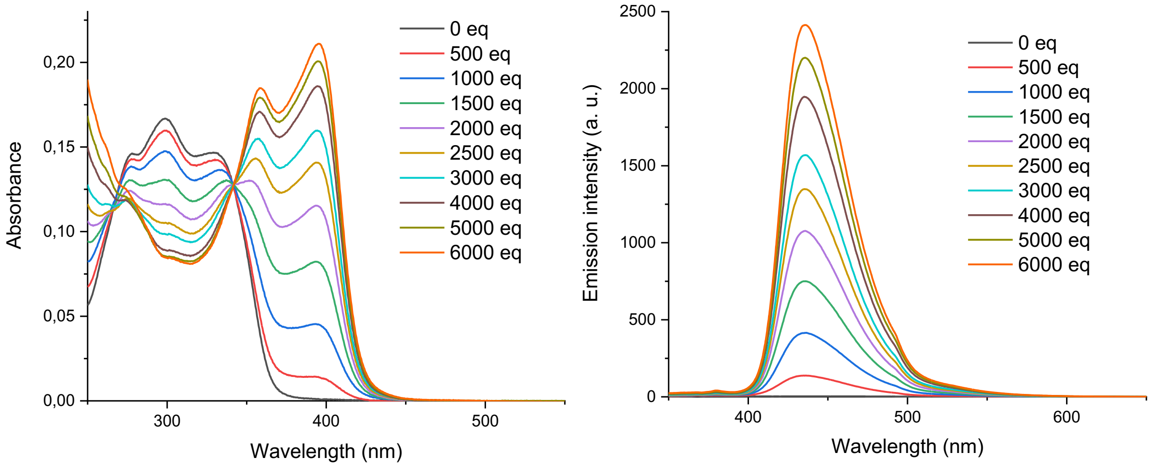

Figure S14. Changes in the absorption (left) and emission (right, λ_{exc} = 329 nm) spectra of a CH_2Cl_2 solution of **6a** ($c = 5.00 \times 10^{-6}$ M) upon addition of TFA.

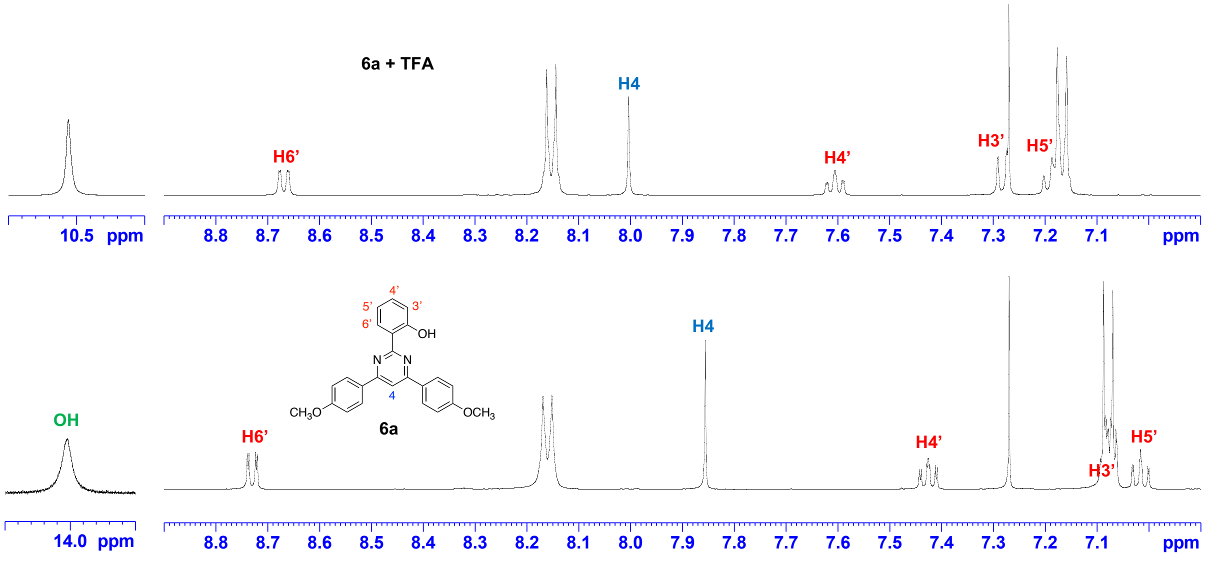

Figure S15. Expanded regions of the ^{1}H NMR spectrum of **6a** before (bottom) and after (top) the addition of an excess of TFA ($CDCl_3$, 500 MHz).

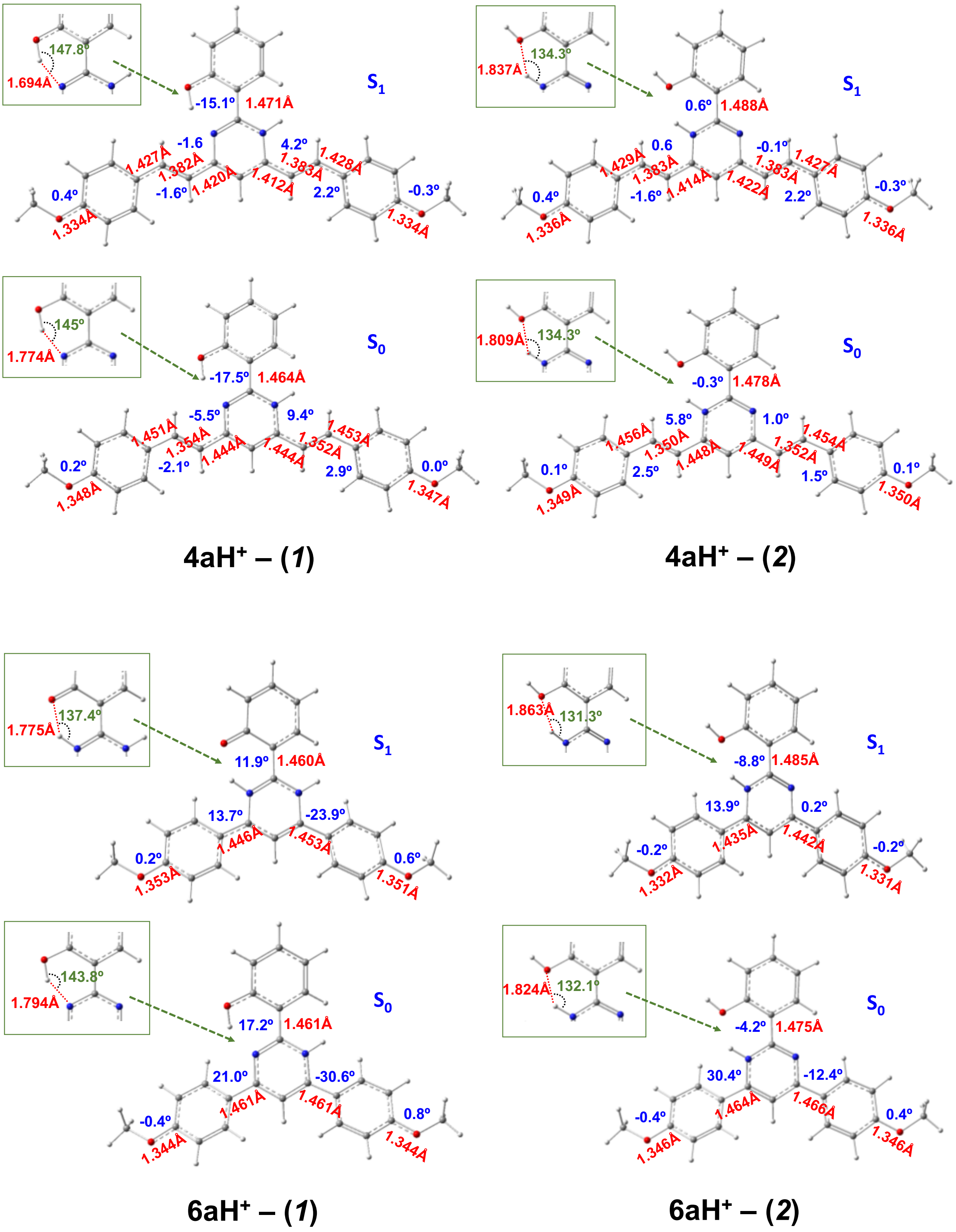

Figure S16. Selected bond lengths and dihedral angles in the S_0 and S_1 states for protonated **4a** and **6a** calculated in CH_2Cl_2 at the M06-2X/6-31+G** level of theory.

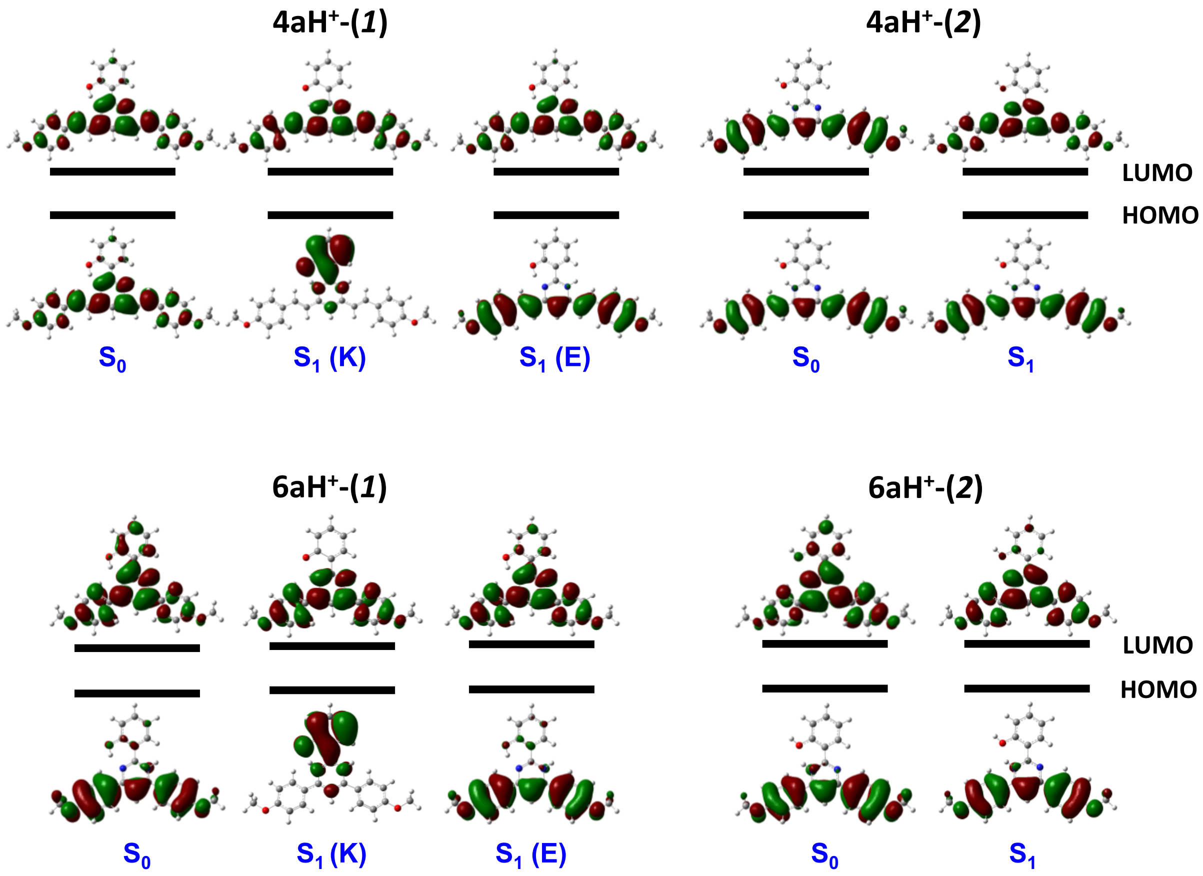

Figure S17. Frontier molecular orbitals calculated for protonated **4a** and **6a** in CH_2Cl_2 solution in the ground state S_0 and excited state S_1 calculated at the M06-2X/6-31+G** level of theory (isocontour plots 0.02 a.u.).

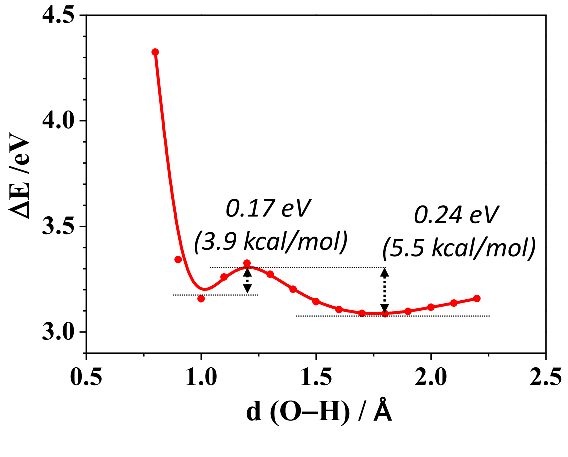

Figure S18. Potential energy surface of the excited state S_1 state for **6aH+-(*1*)** in CH_2Cl_2 solution calculated at the M06-2X/6-31+G** level of theory.

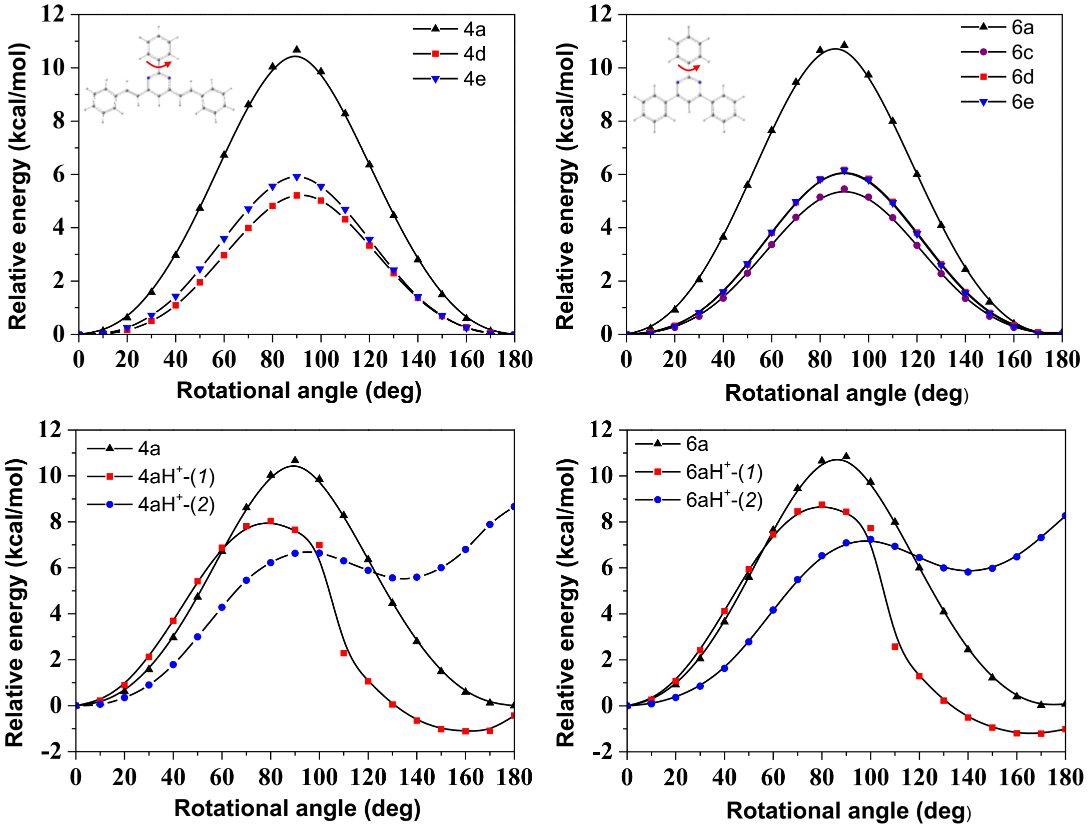

Figure S19. Relative rotational energy barrier of the phenyl ring in the ground state calculated at the M06-2X/6-31+G** level of theory in CH_2Cl_2 solution.

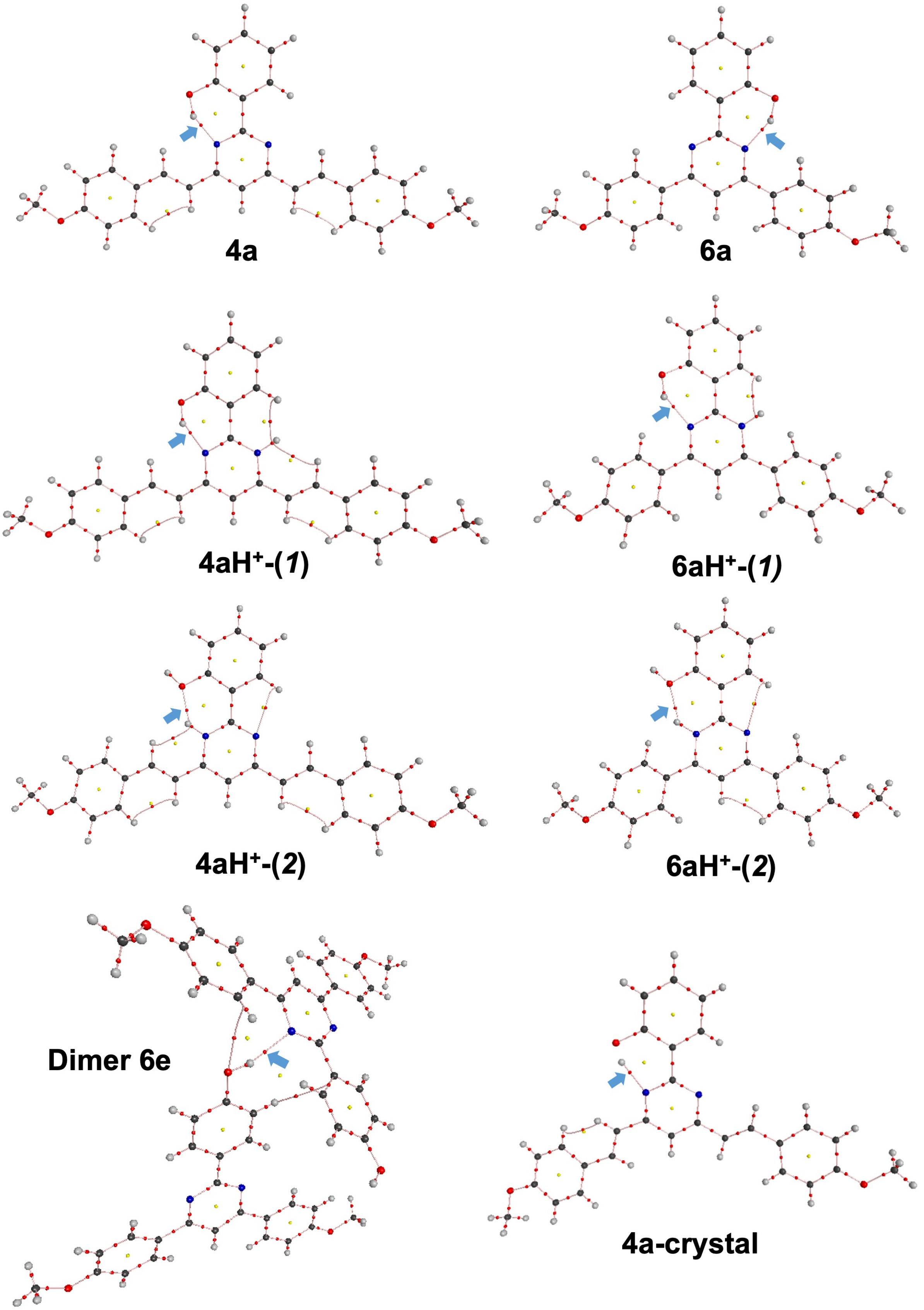

Figure S20. Molecular graphs from the QTAIM analysis. The spheres represent the nuclei of atoms: black = C, red = O, blue = N, gray = H. The lines connecting the nuclei are bond paths and the small dots represent the critical points: red = BCP (bond critical point), yellow = RCP (ring critical point).

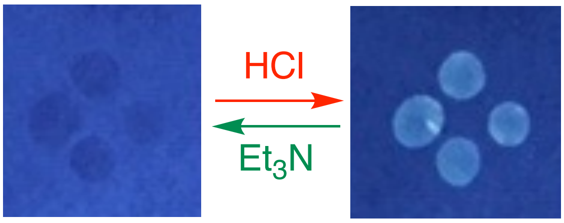

Figure S21. Digital photographs of the reversible color change of Whatman filter paper under UV light (365 nm) using compound **6a** as an anti-counterfeiting agent.

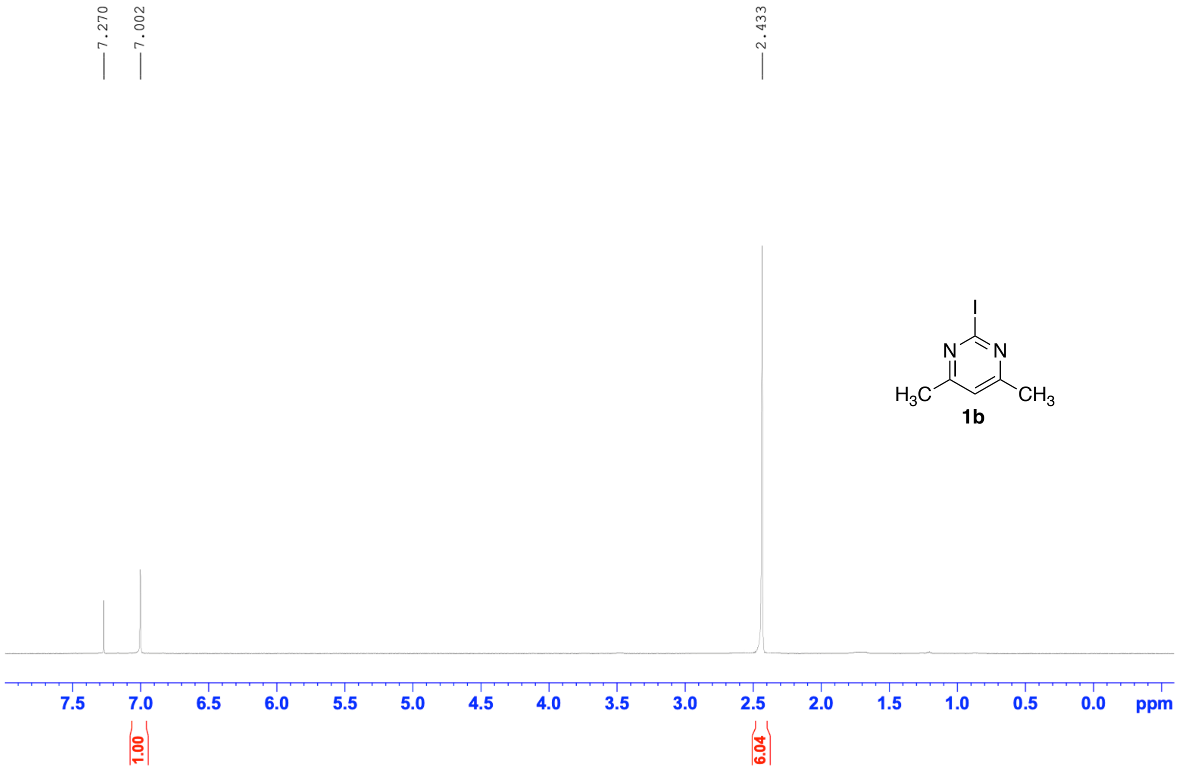

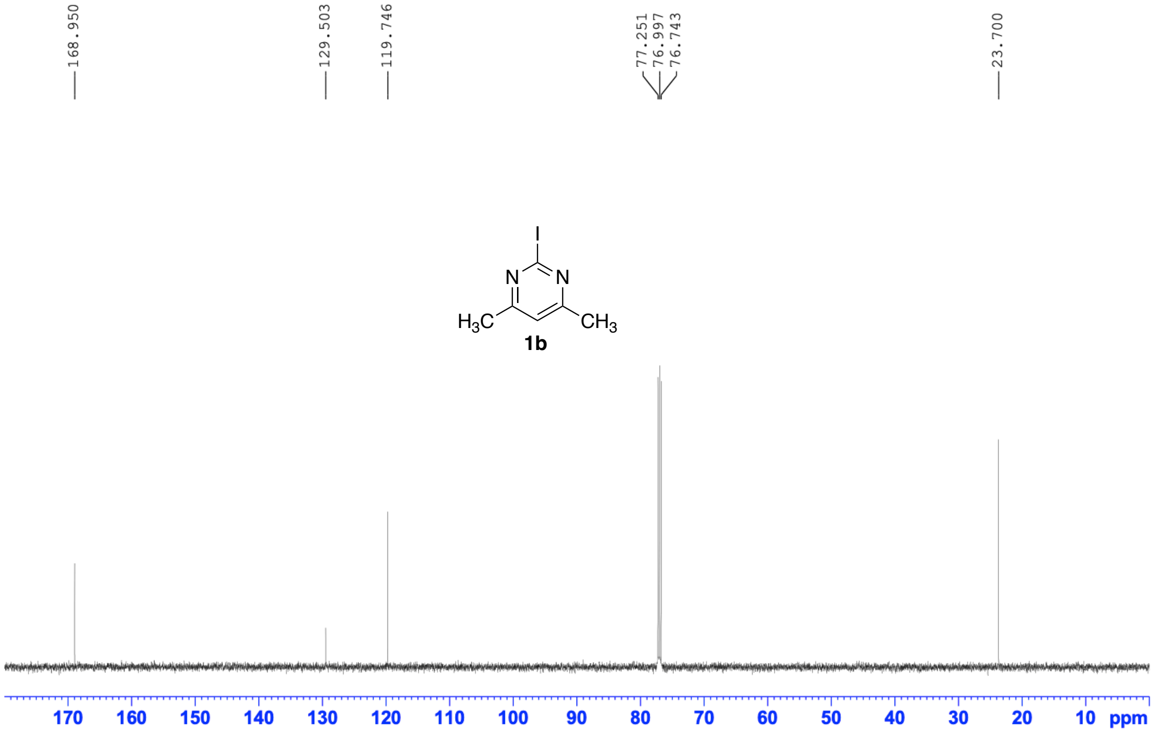

Figure S22. ^{1}H NMR ($CDCl_3$, 500 MHz) and ^{13}C NMR spectra of ($CDCl_3$, 125 MHz) of **1b**.

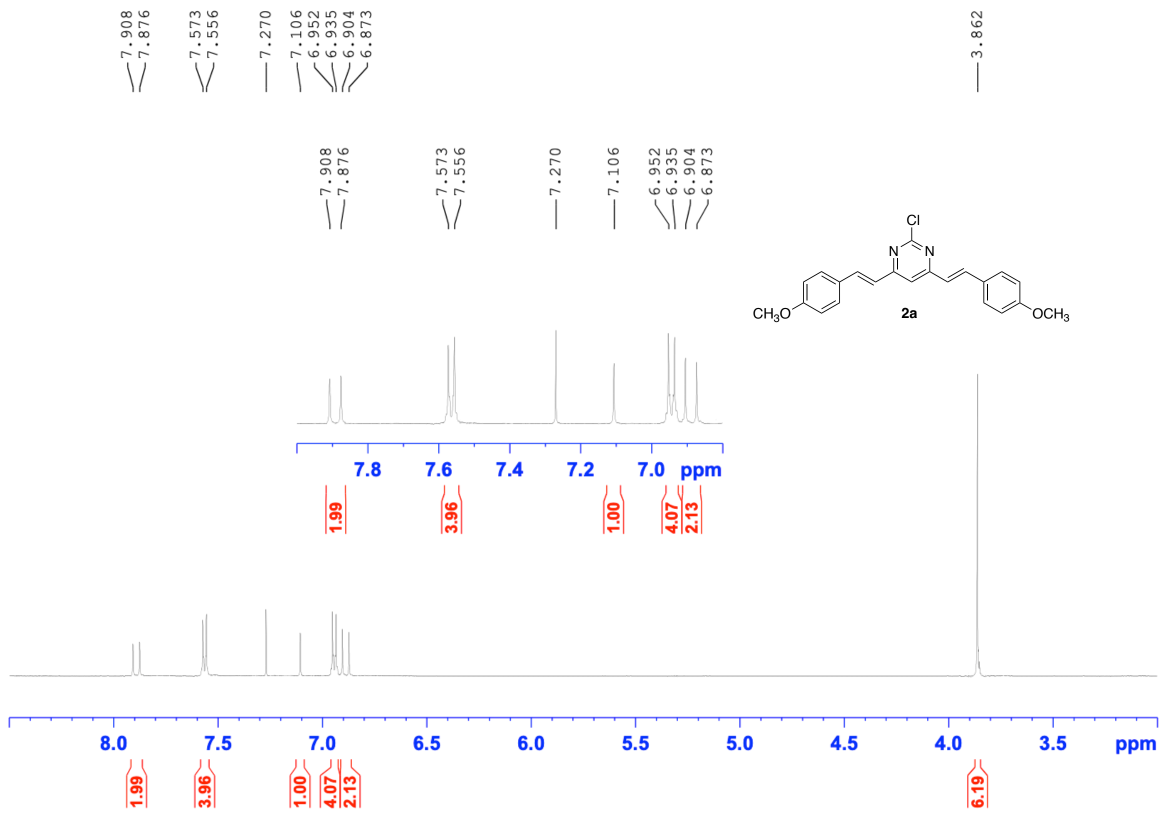

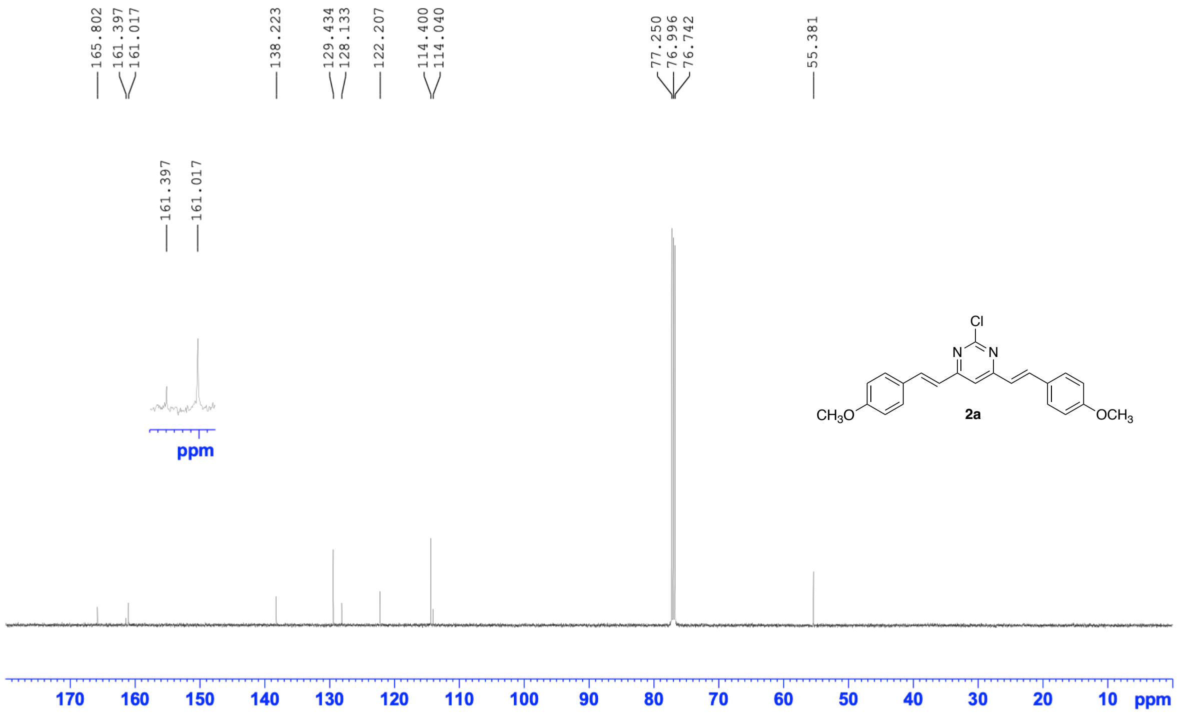

Figure S23. ^{1}H NMR ($CDCl_3$, 500 MHz) and ^{13}C NMR spectra of ($CDCl_3$, 125 MHz) of **2a**.

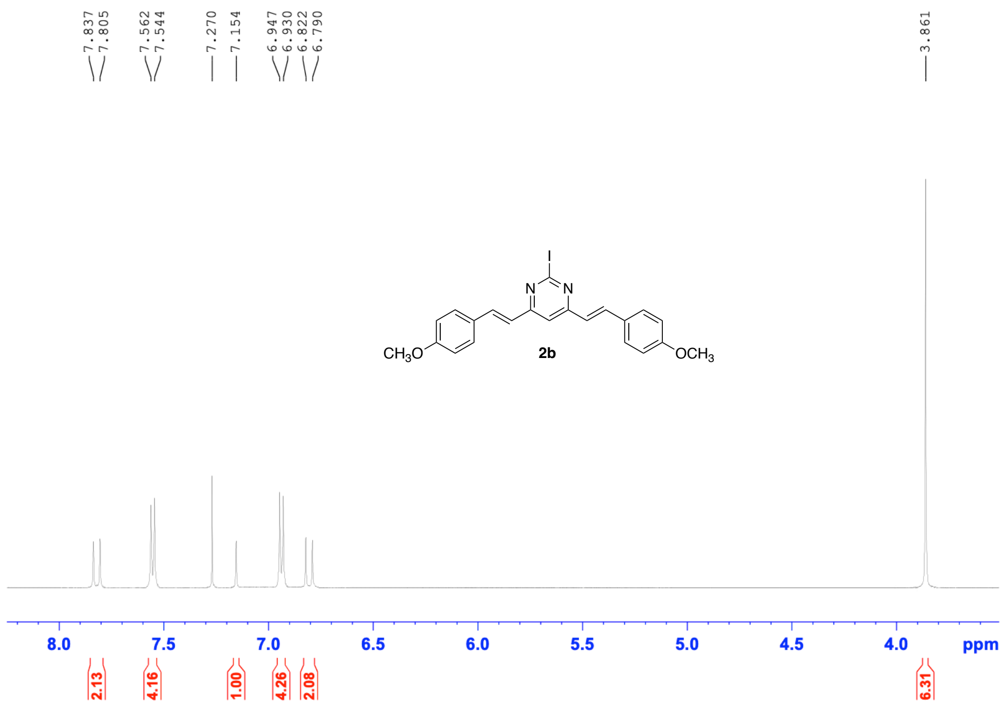

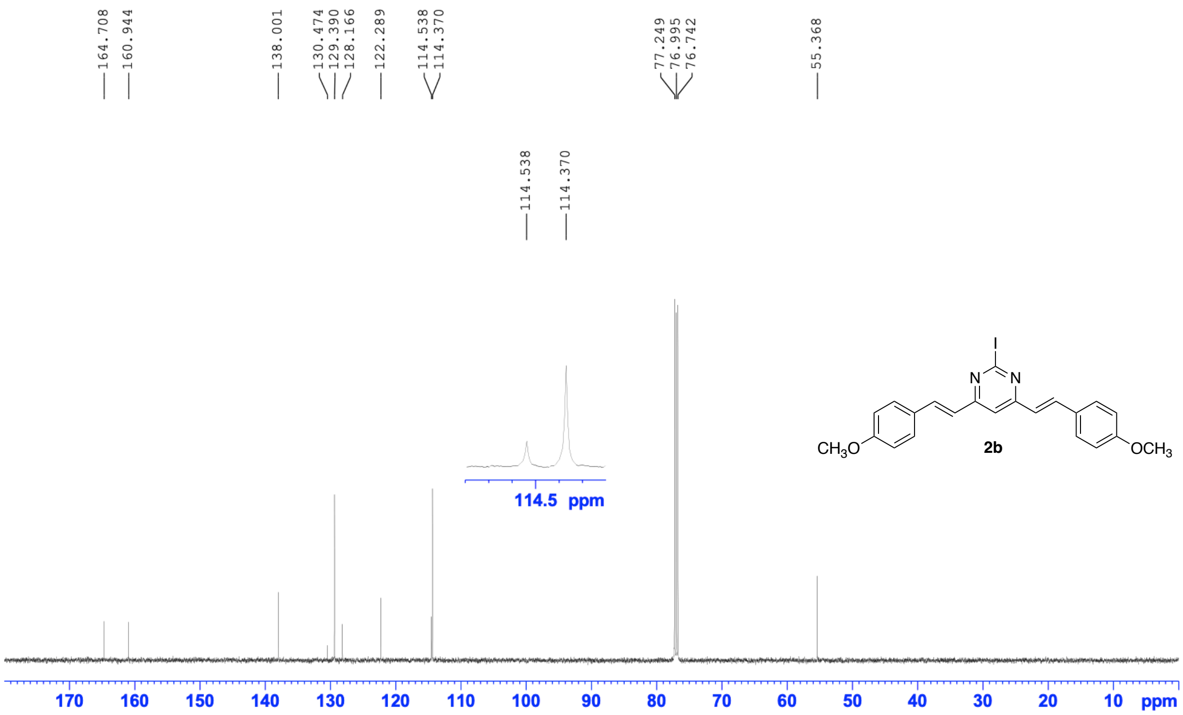

Figure S24. ^{1}H NMR ($CDCl_3$, 500 MHz) and ^{13}C NMR spectra of ($CDCl_3$, 125 MHz) of **2b**.

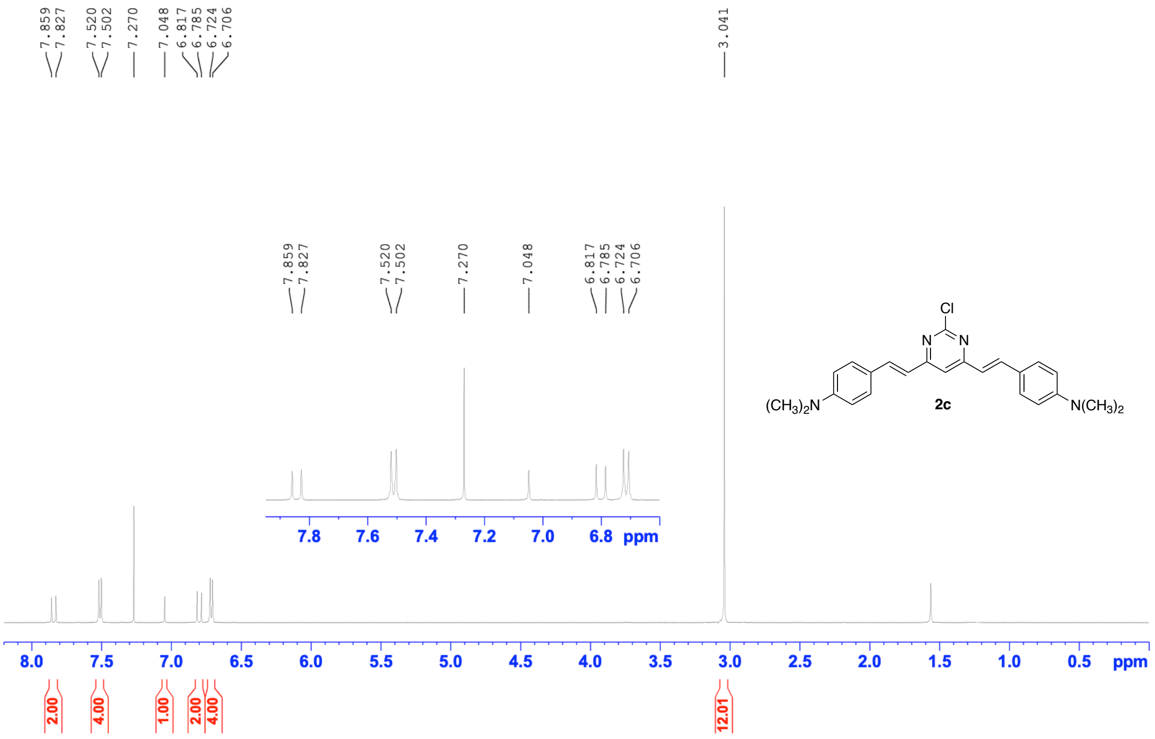

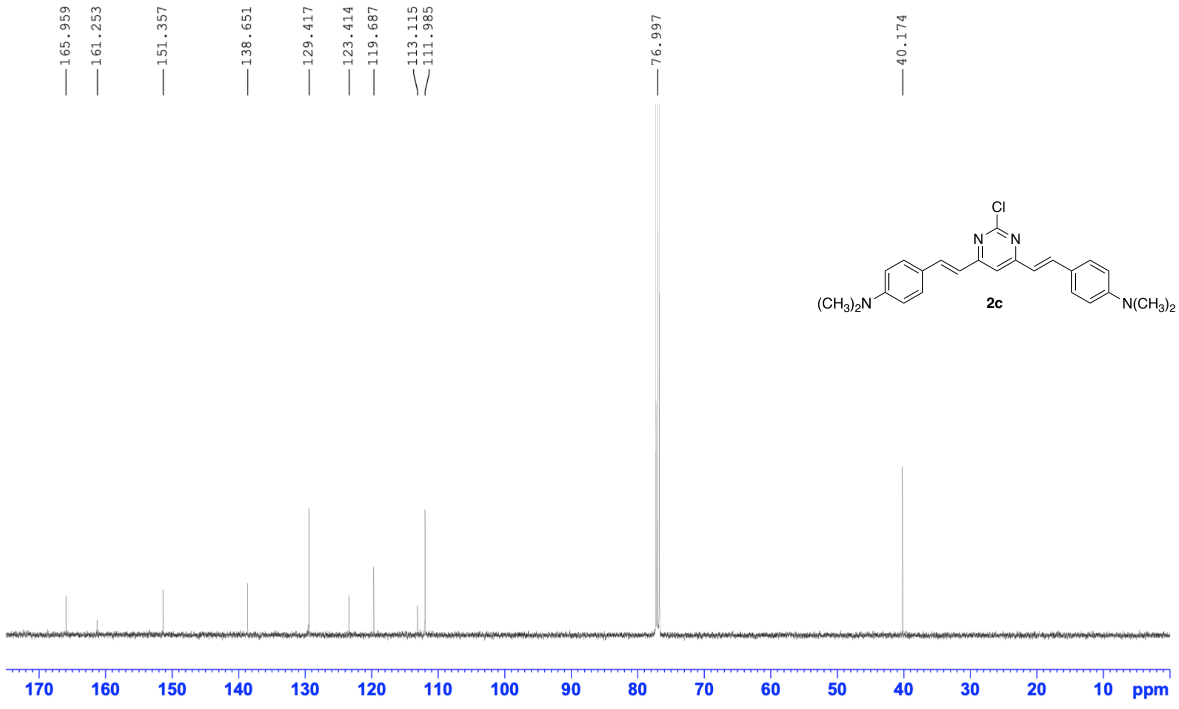

Figure S25. ^{1}H NMR ($CDCl_3$, 500 MHz) and ^{13}C NMR spectra of ($CDCl_3$, 125 MHz) of **2c**.

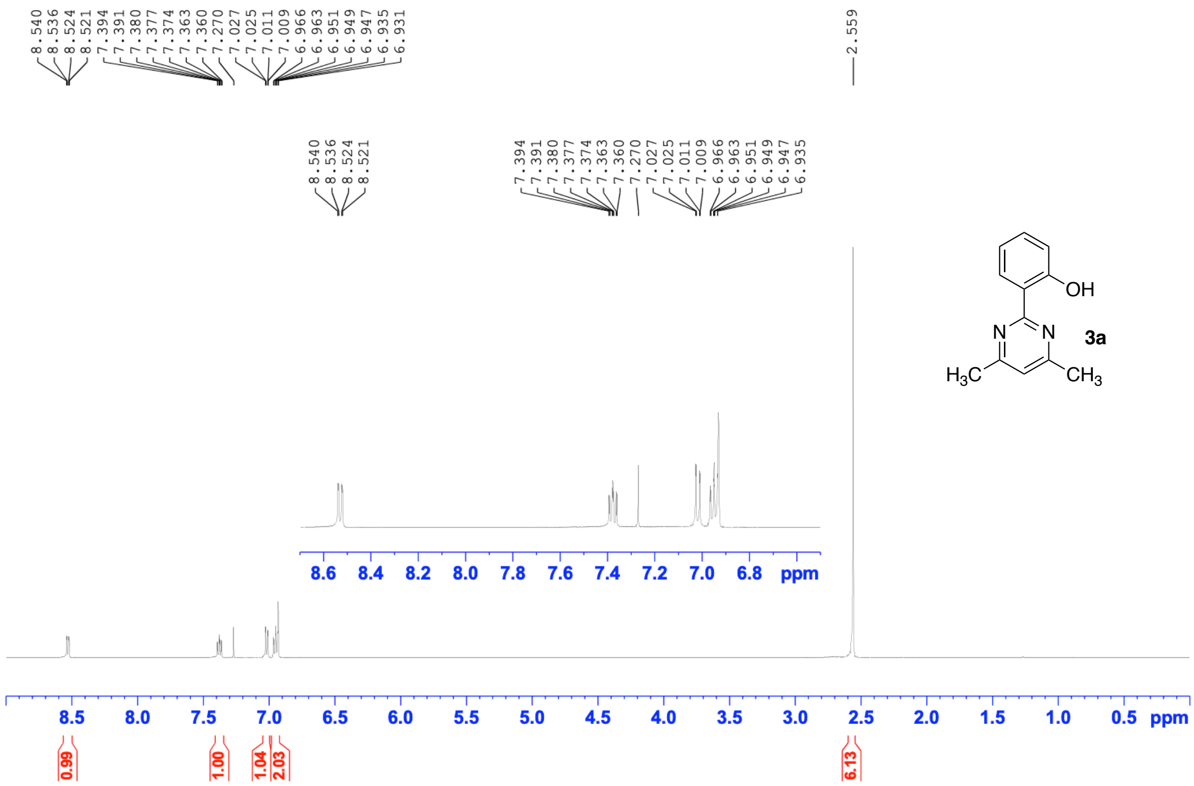

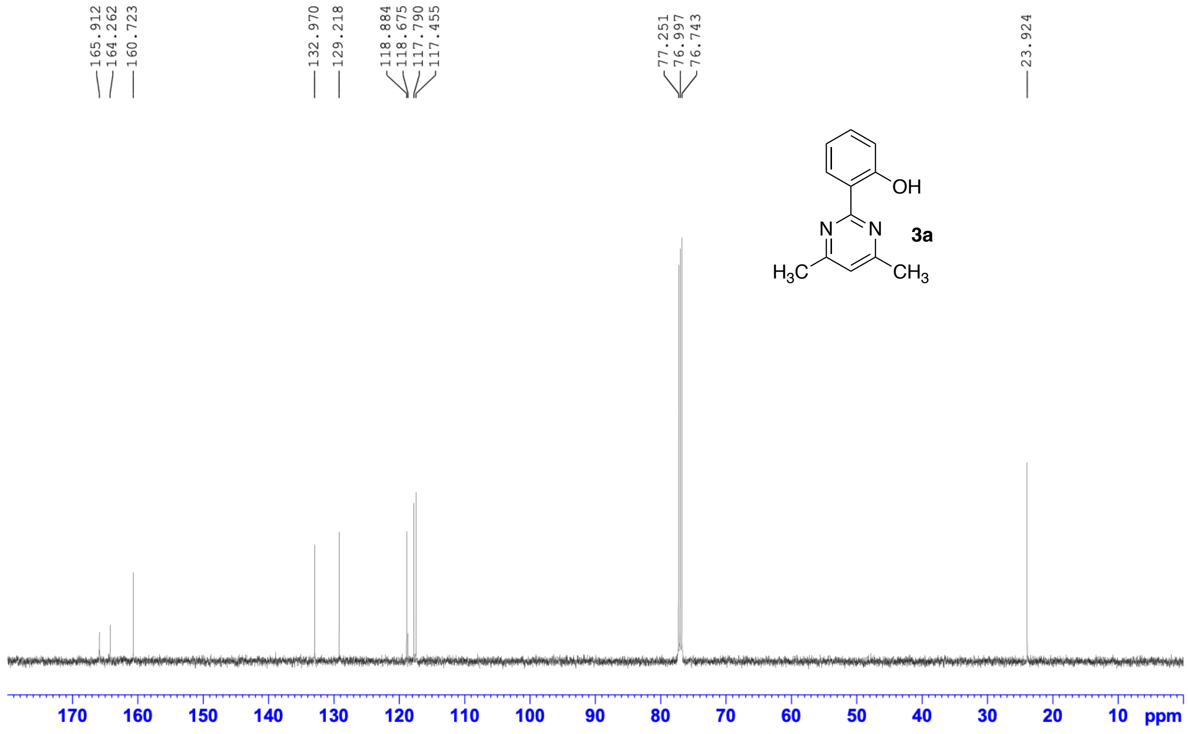

Figure S26. ^{1}H NMR ($CDCl_3$, 500 MHz) and ^{13}C NMR spectra of ($CDCl_3$, 125 MHz) of **3a**.

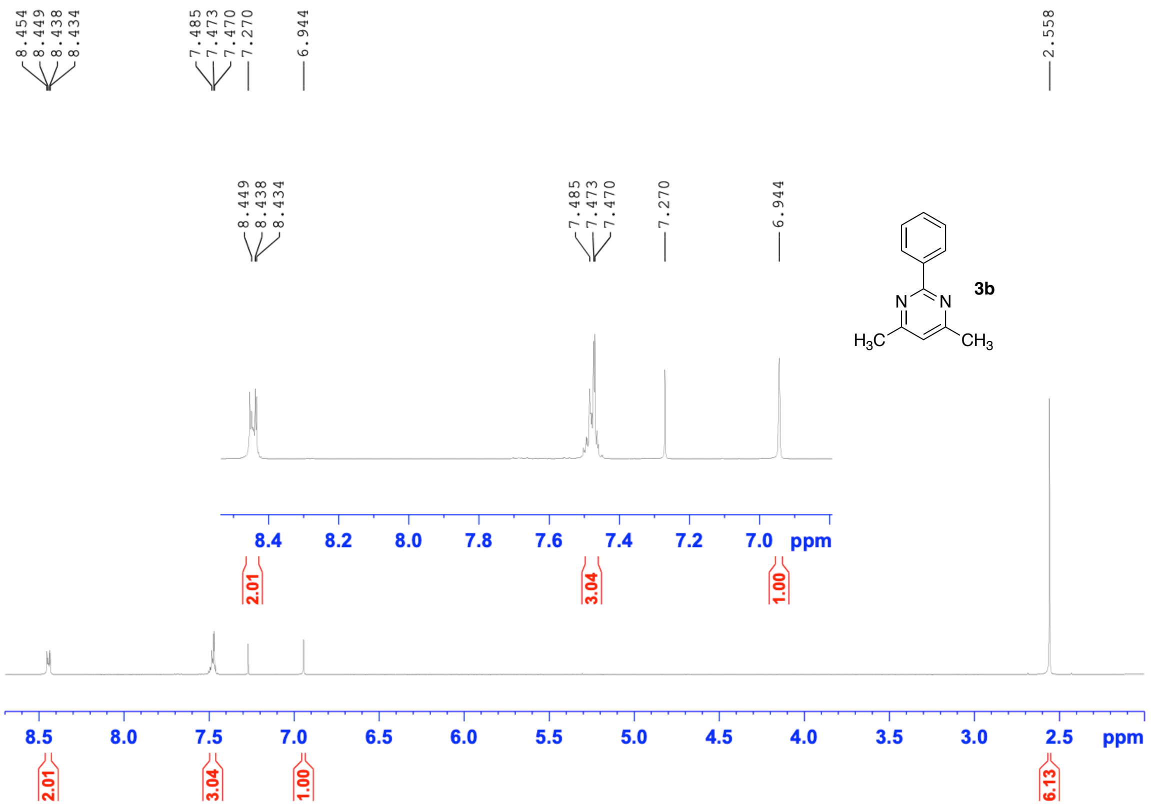

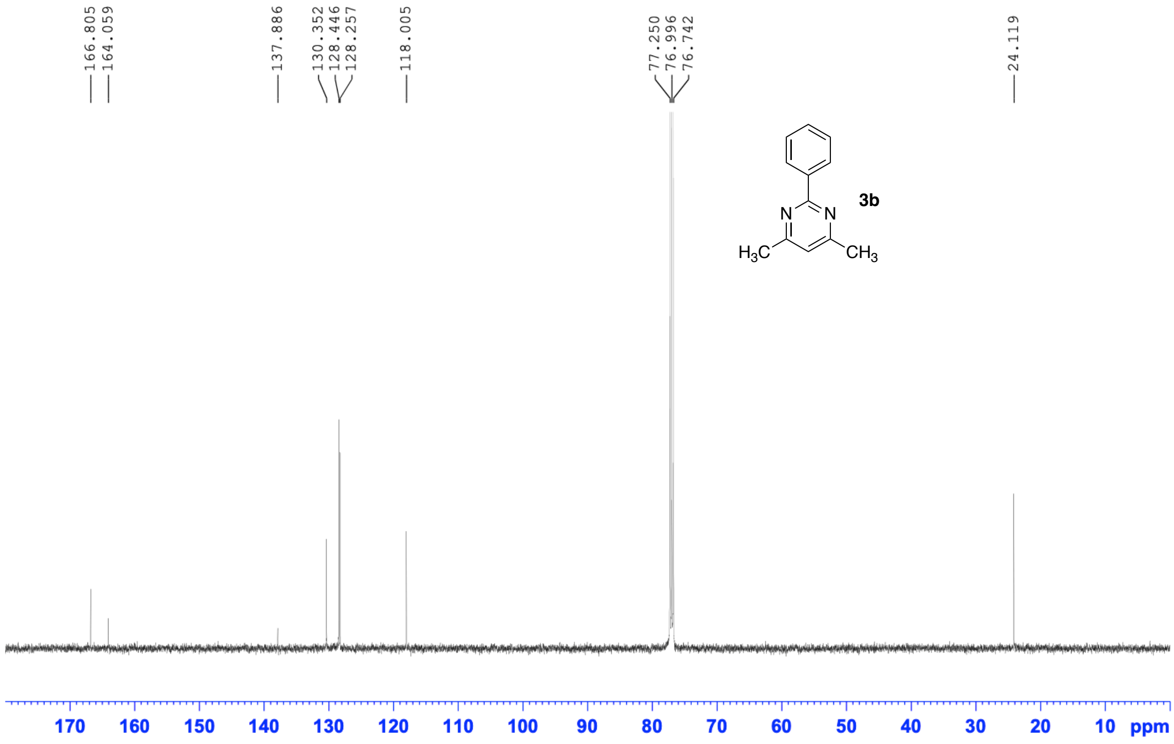

Figure S27. ^{1}H NMR ($CDCl_3$, 500 MHz) and ^{13}C NMR spectra of ($CDCl_3$, 125 MHz) of **3b**.

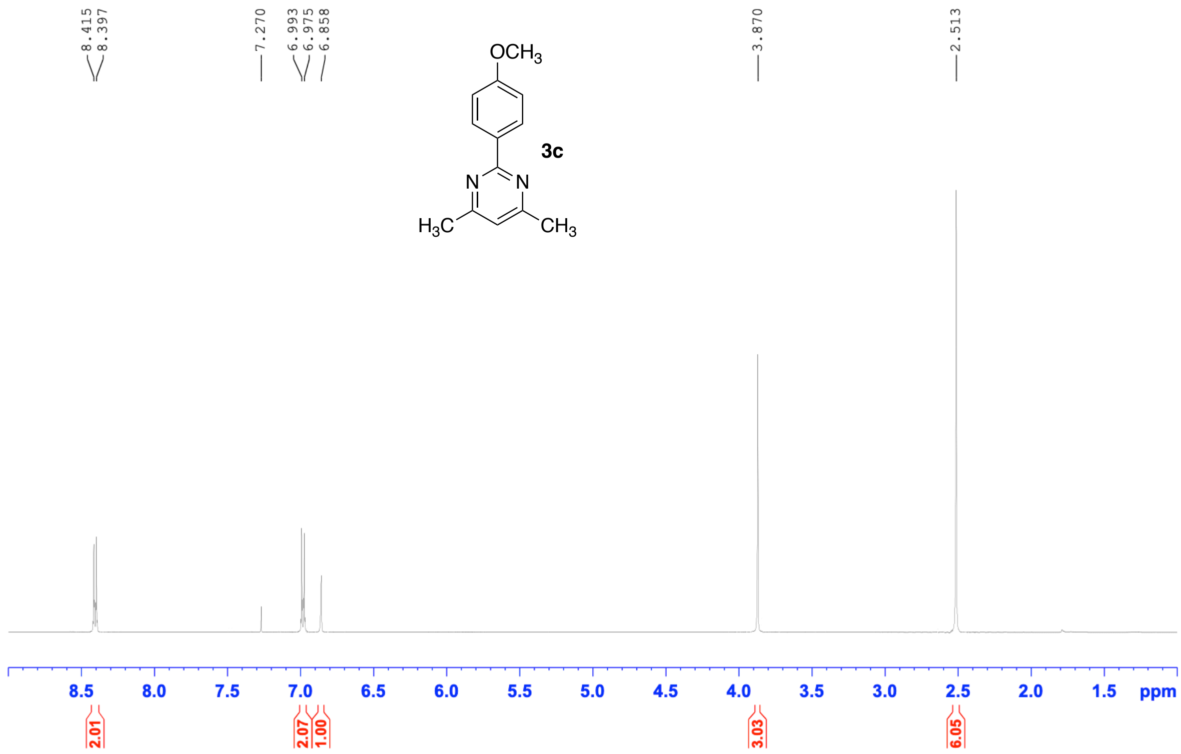

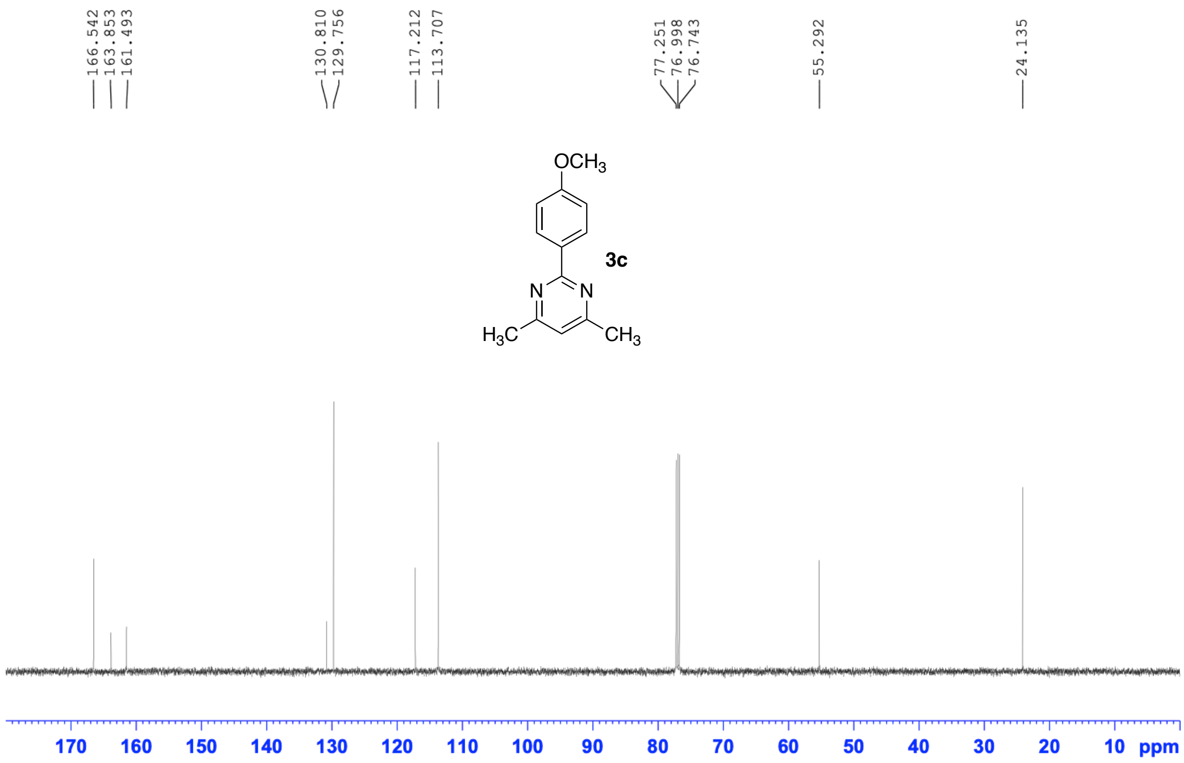

Figure S28. ^{1}H NMR ($CDCl_3$, 500 MHz) and ^{13}C NMR spectra of ($CDCl_3$, 125 MHz) of **3c**.

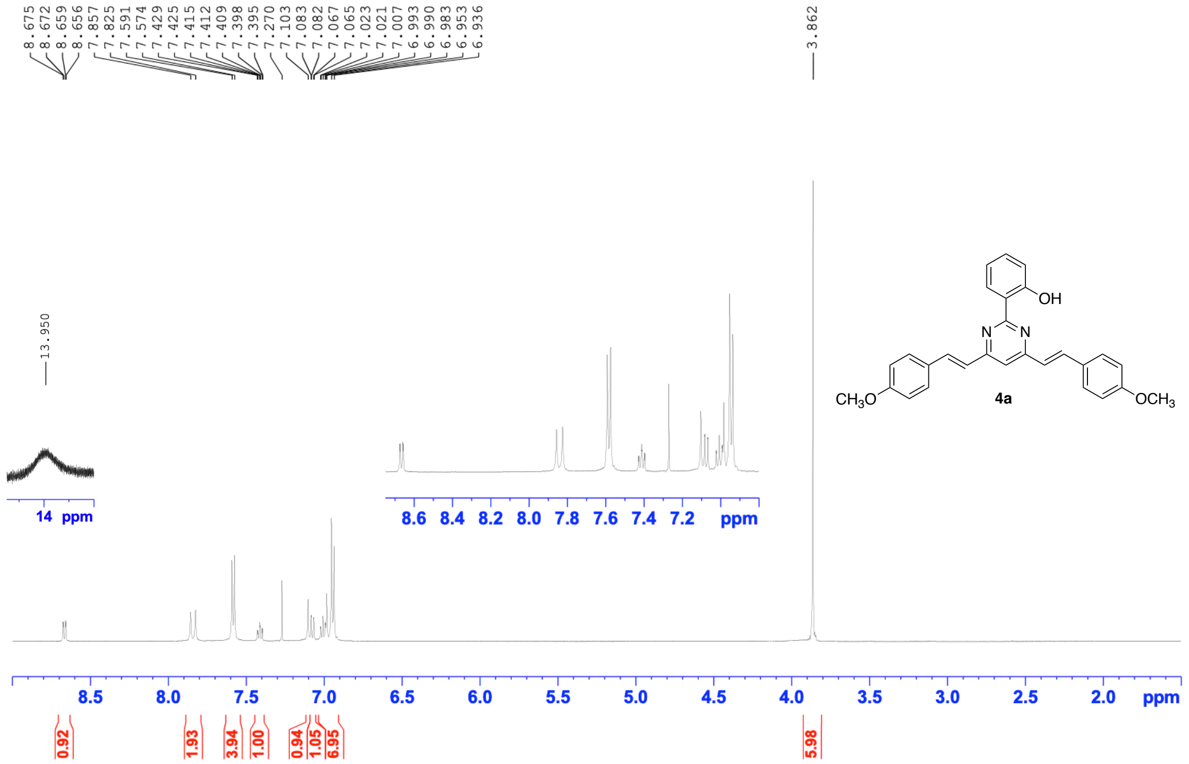

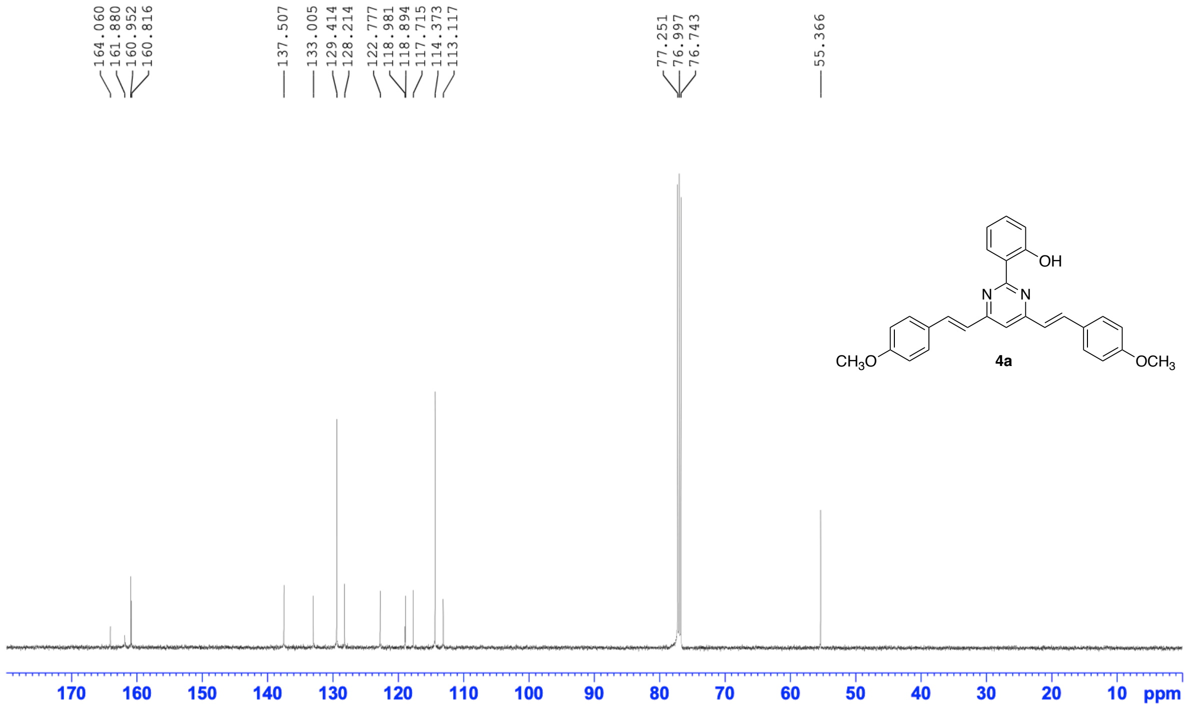

Figure S29. ^{1}H NMR ($CDCl_3$, 500 MHz) and ^{13}C NMR spectra of ($CDCl_3$, 125 MHz) of **4a**.

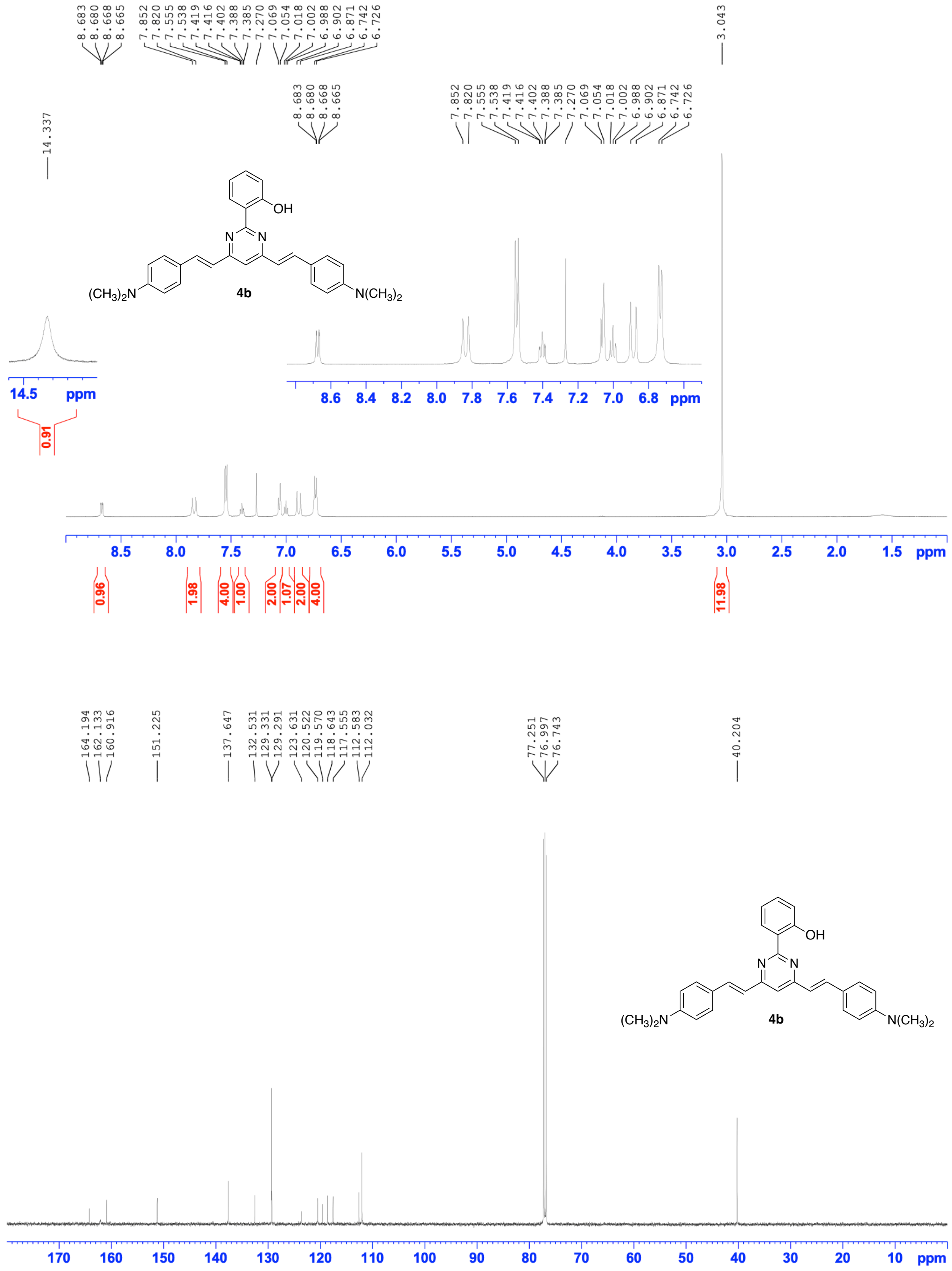

Figure S30. ^{1}H NMR ($CDCl_3$, 500 MHz) and ^{13}C NMR spectra of ($CDCl_3$, 125 MHz) of **4b**.

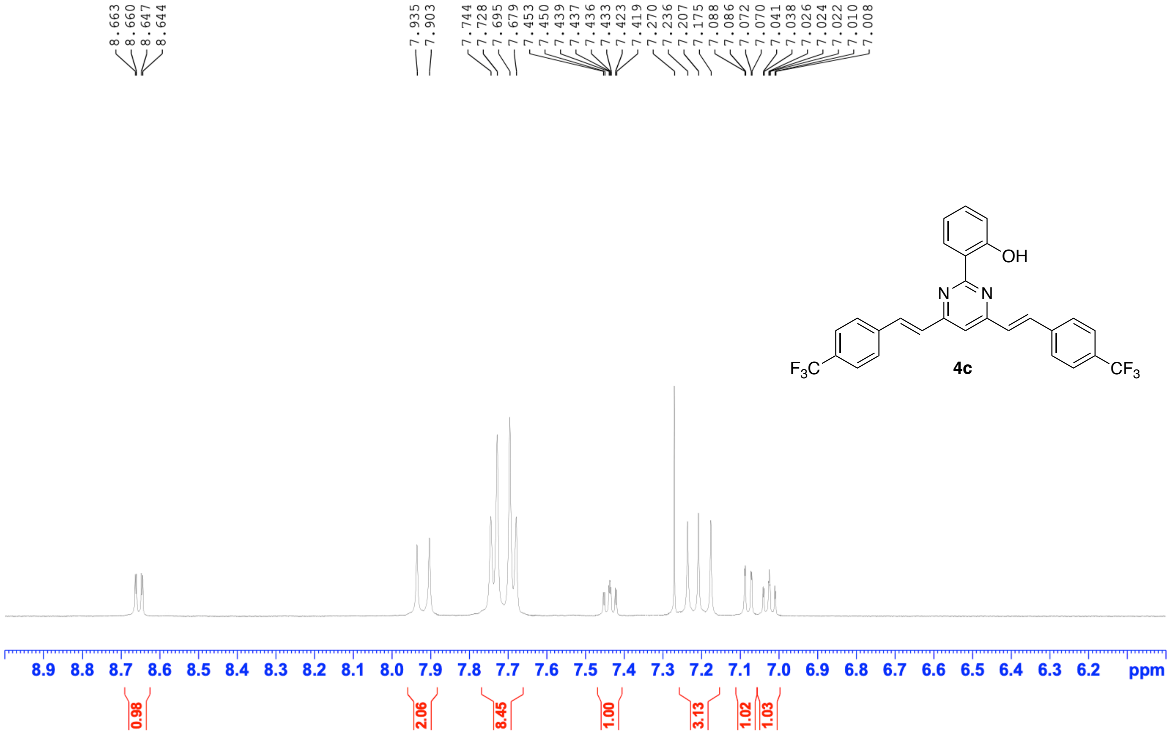

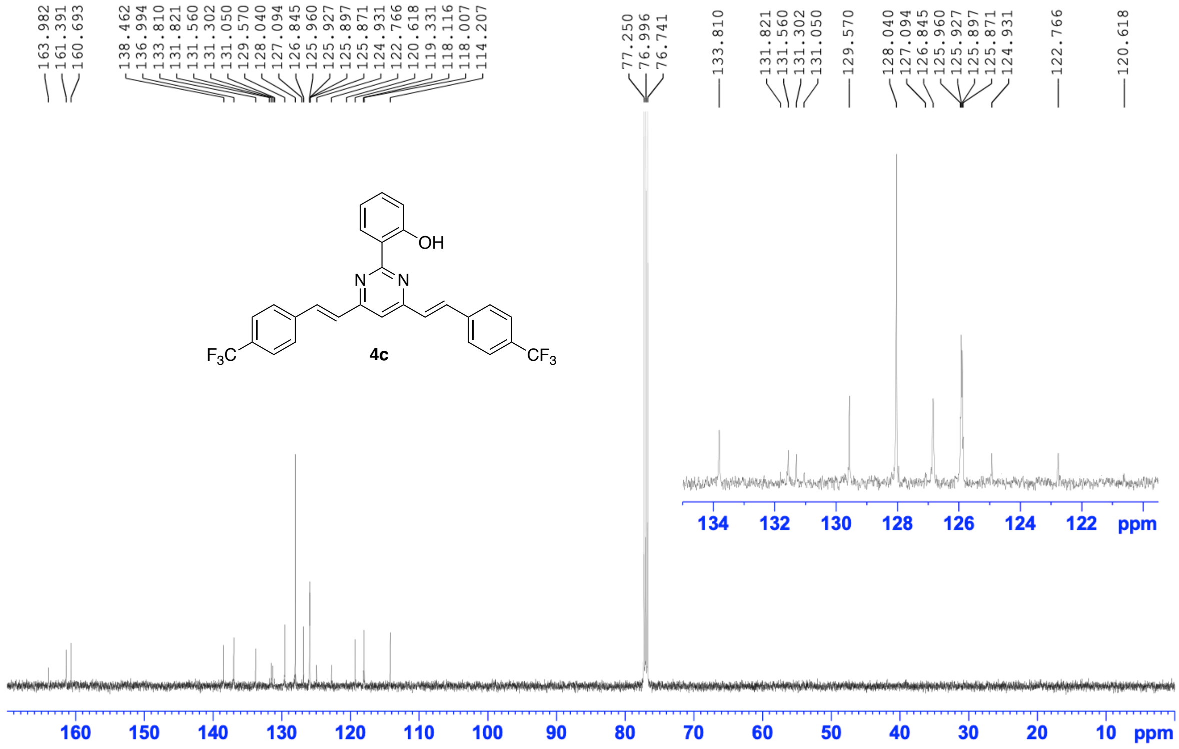

Figure S31. ^{1}H NMR ($CDCl_3$, 500 MHz) and ^{13}C NMR spectra of ($CDCl_3$, 125 MHz) of **4c**.

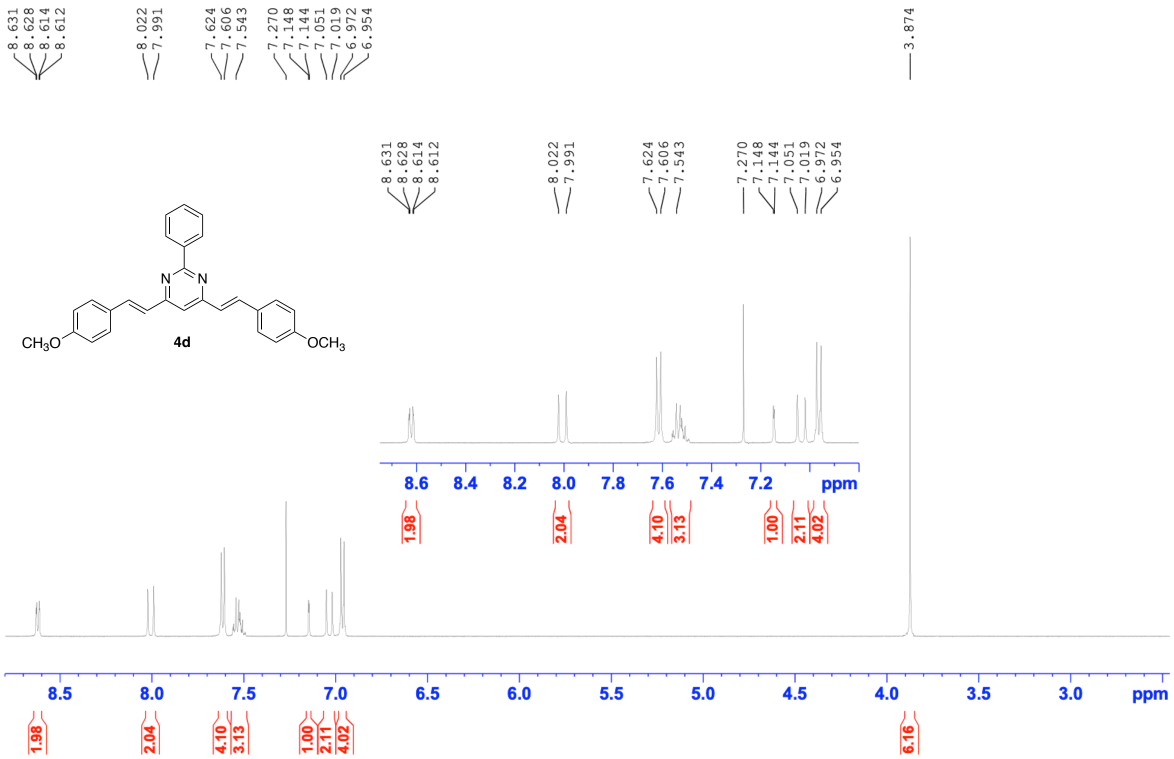

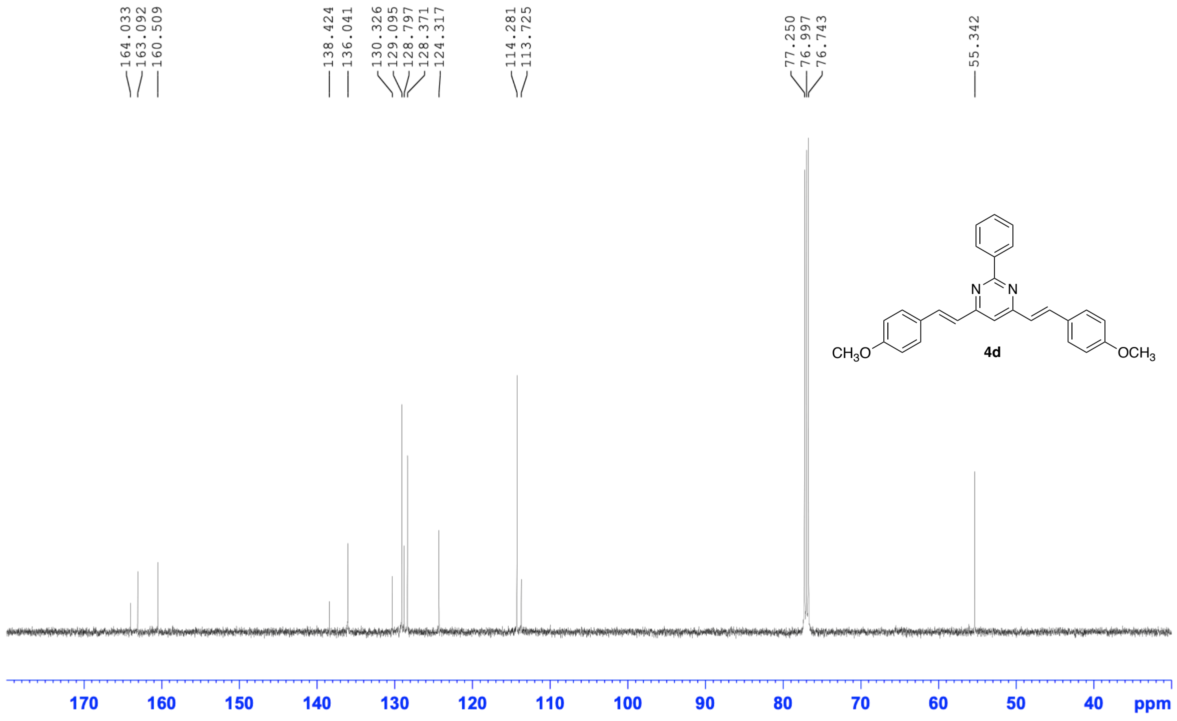

Figure S32. ^{1}H NMR ($CDCl_3$, 500 MHz) and ^{13}C NMR spectra of ($CDCl_3$, 125 MHz) of **4d**.

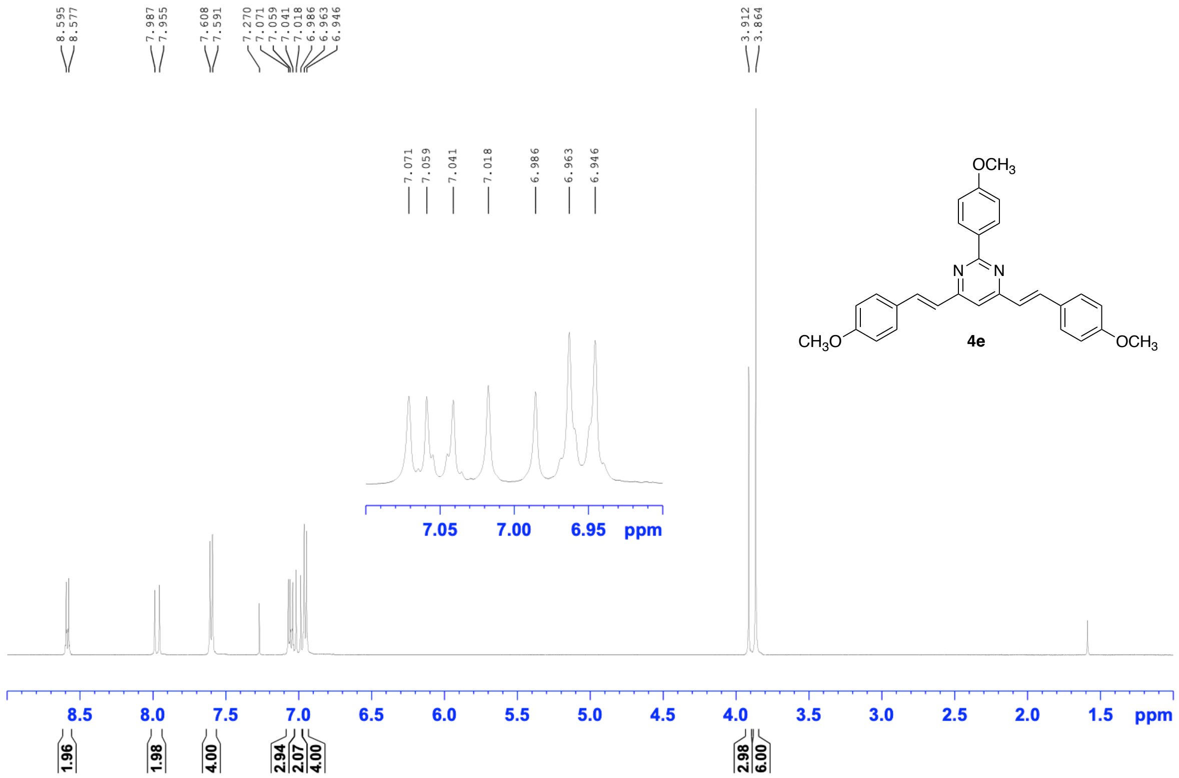

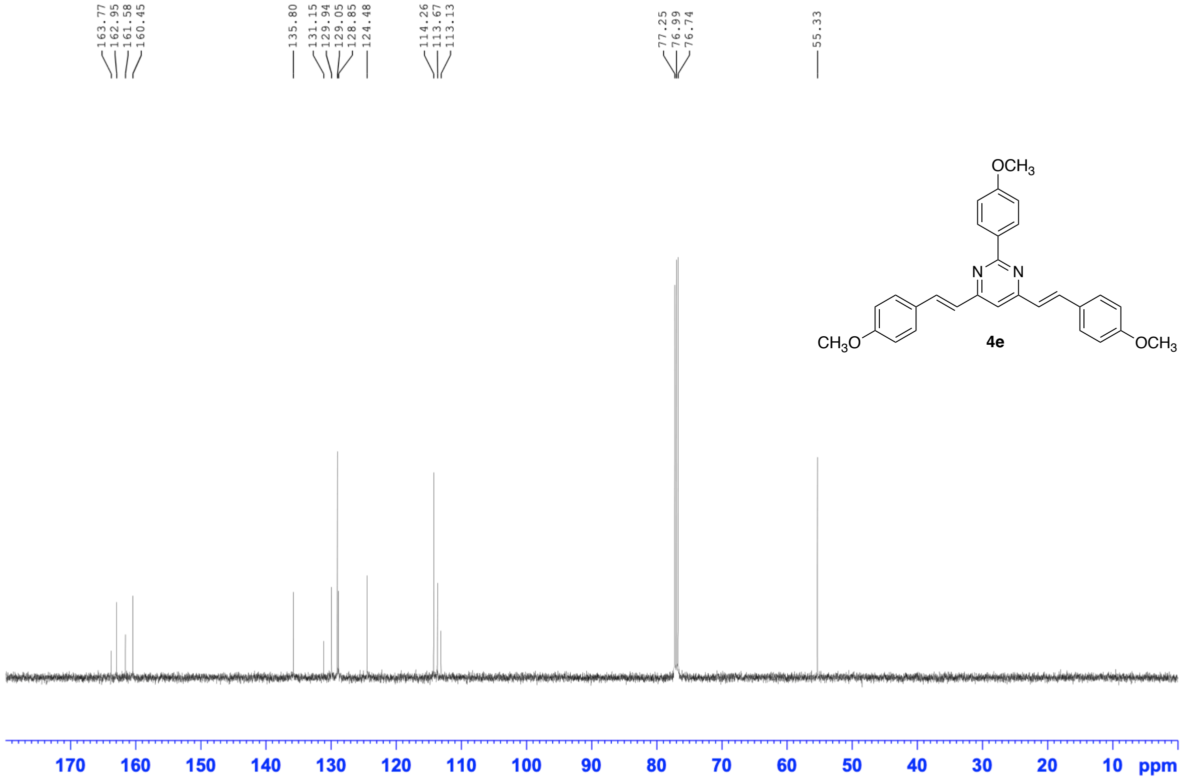

Figure S33. ^{1}H NMR ($CDCl_3$, 500 MHz) and ^{13}C NMR spectra of ($CDCl_3$, 125 MHz) of **4e**.

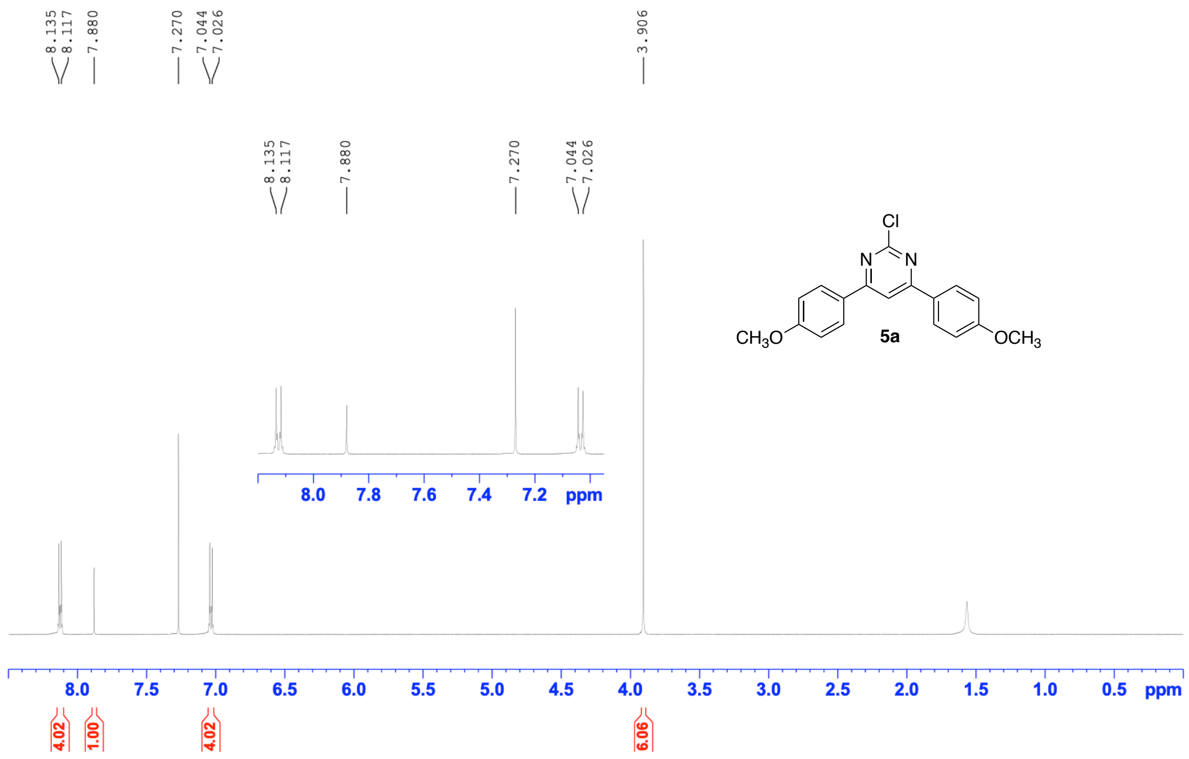

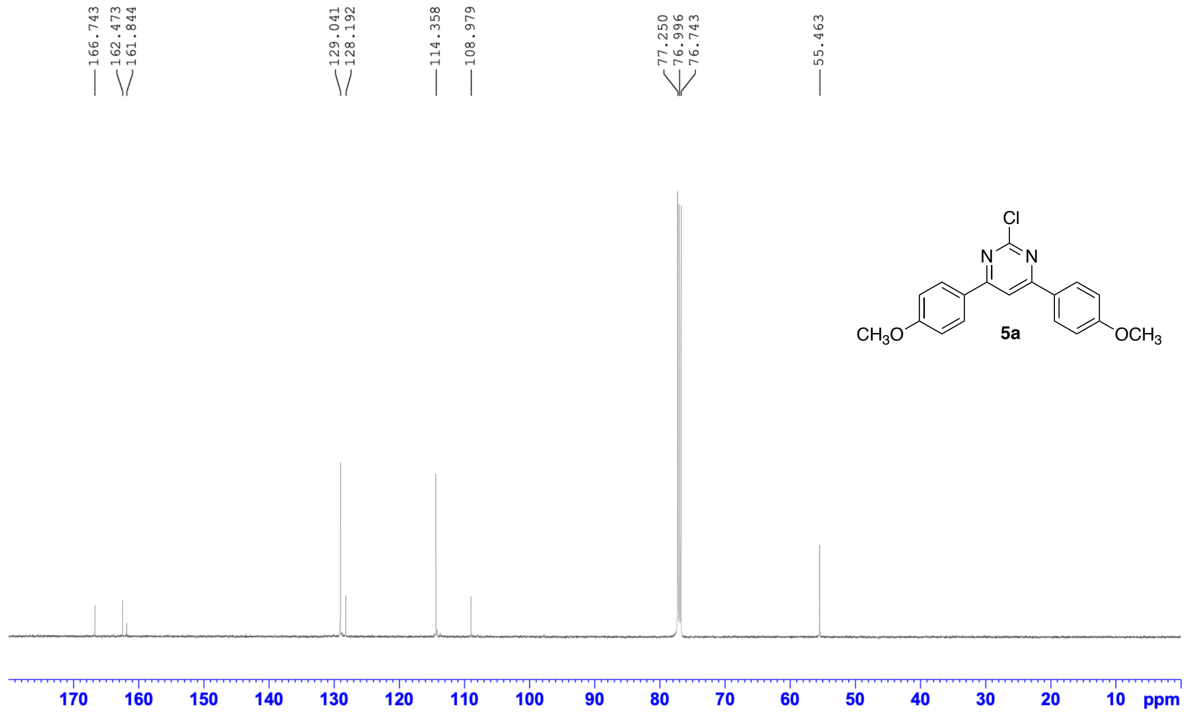

Figure S34. ^{1}H NMR ($CDCl_3$, 500 MHz) and ^{13}C NMR spectra of ($CDCl_3$, 125 MHz) of **5a**.

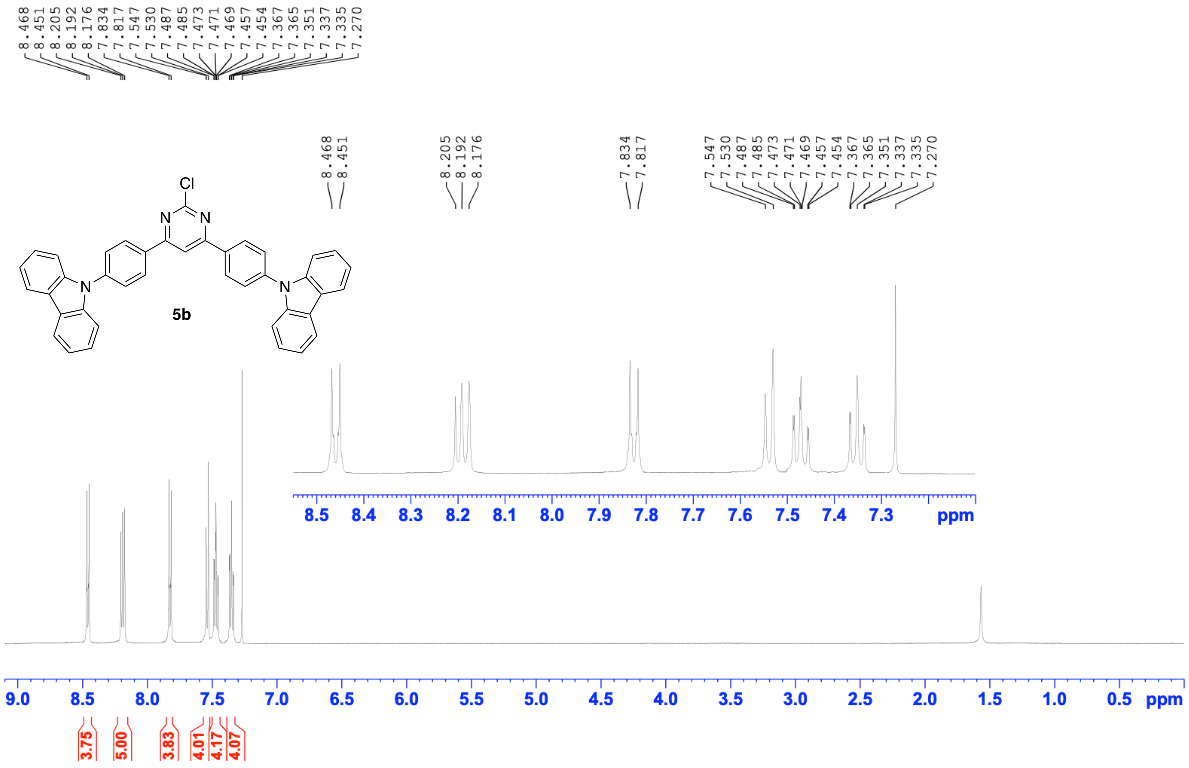

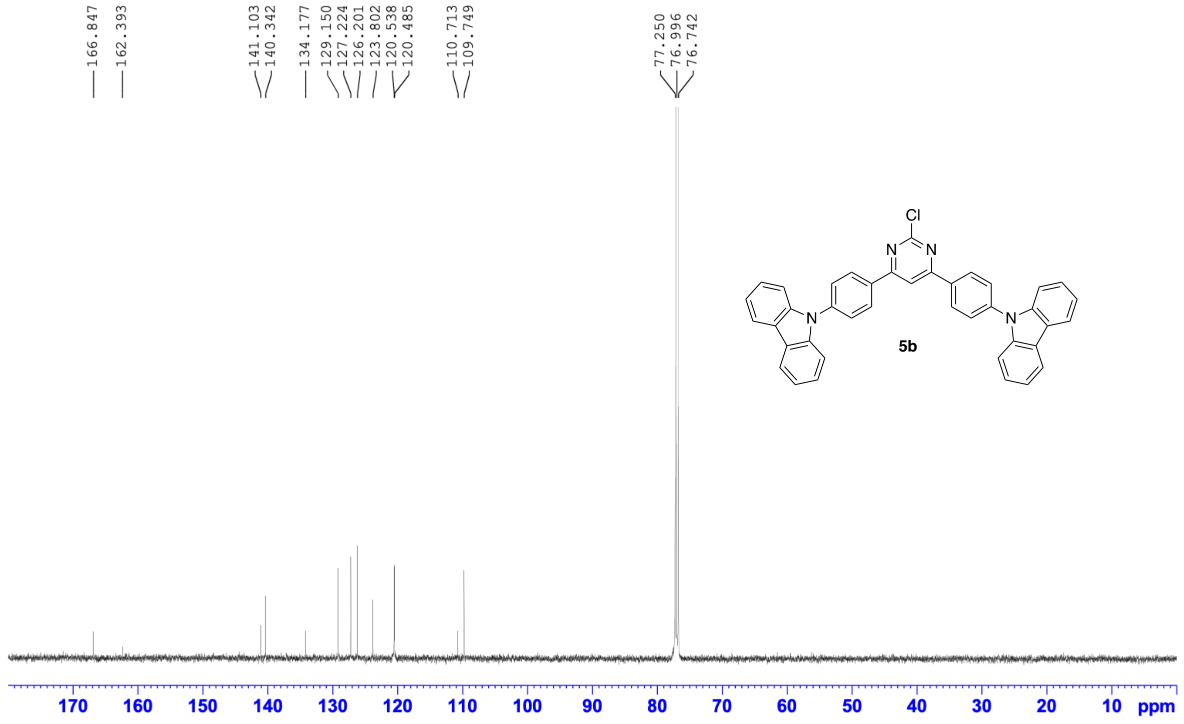

Figure S35. ^{1}H NMR ($CDCl_3$, 500 MHz) and ^{13}C NMR spectra of ($CDCl_3$, 125 MHz) of **5b**.

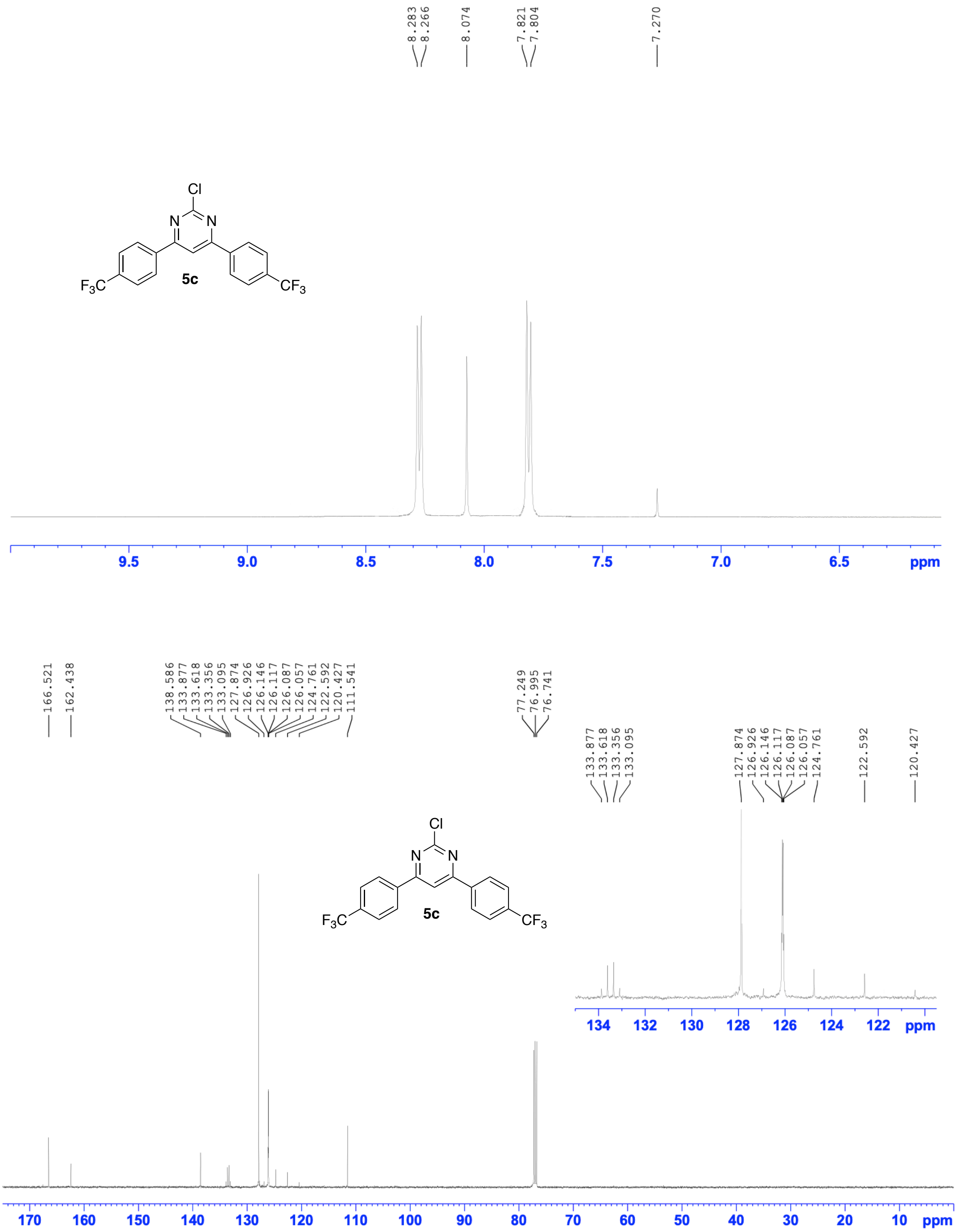

Figure S36. ^{1}H NMR ($CDCl_3$, 500 MHz) and ^{13}C NMR spectra of ($CDCl_3$, 125 MHz) of **5c**.

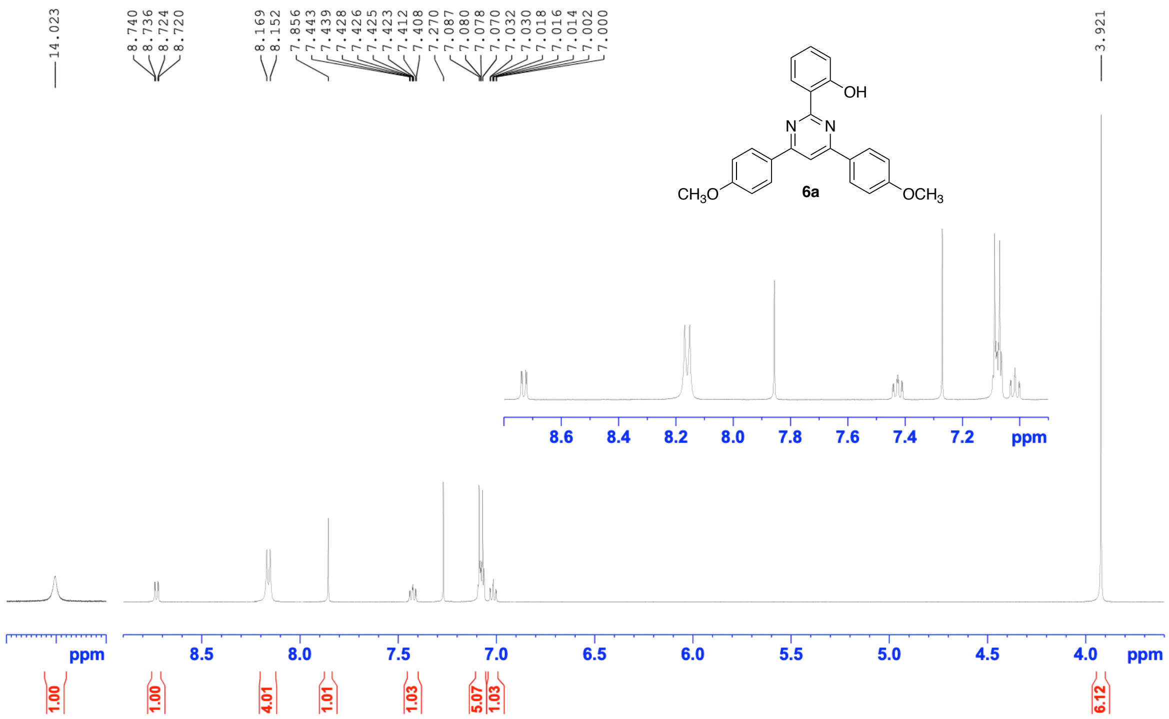

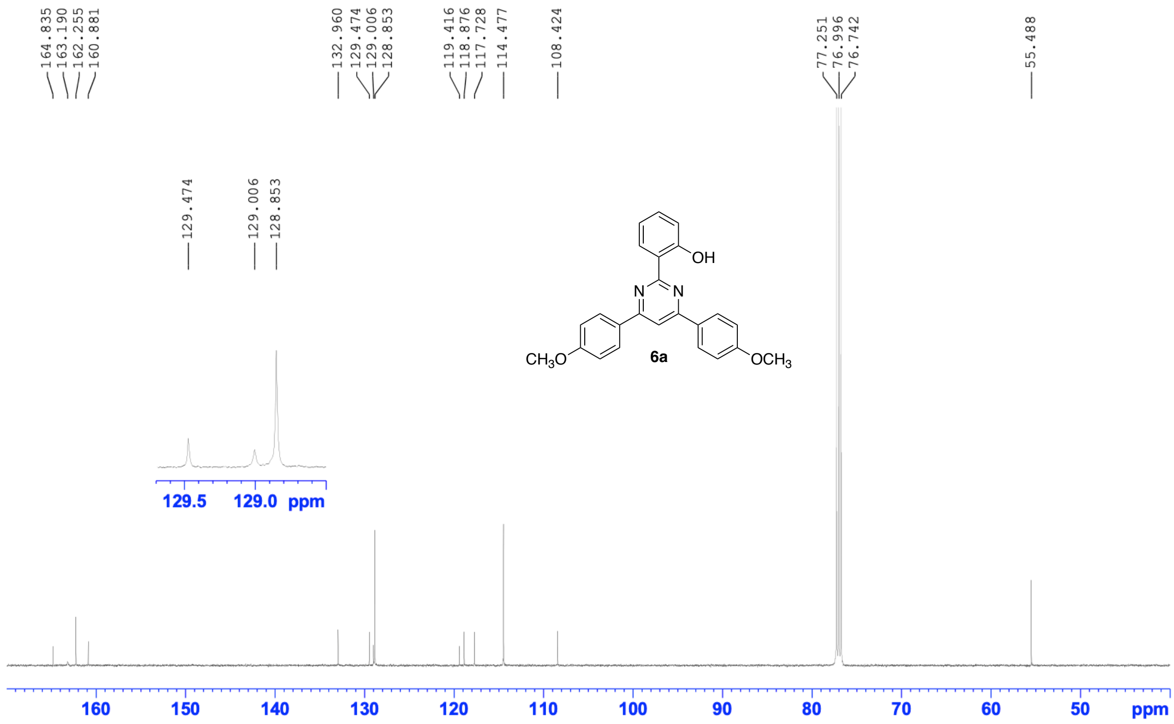

Figure S37. ^{1}H NMR ($CDCl_3$, 500 MHz) and ^{13}C NMR spectra of ($CDCl_3$, 125 MHz) of **6a**.

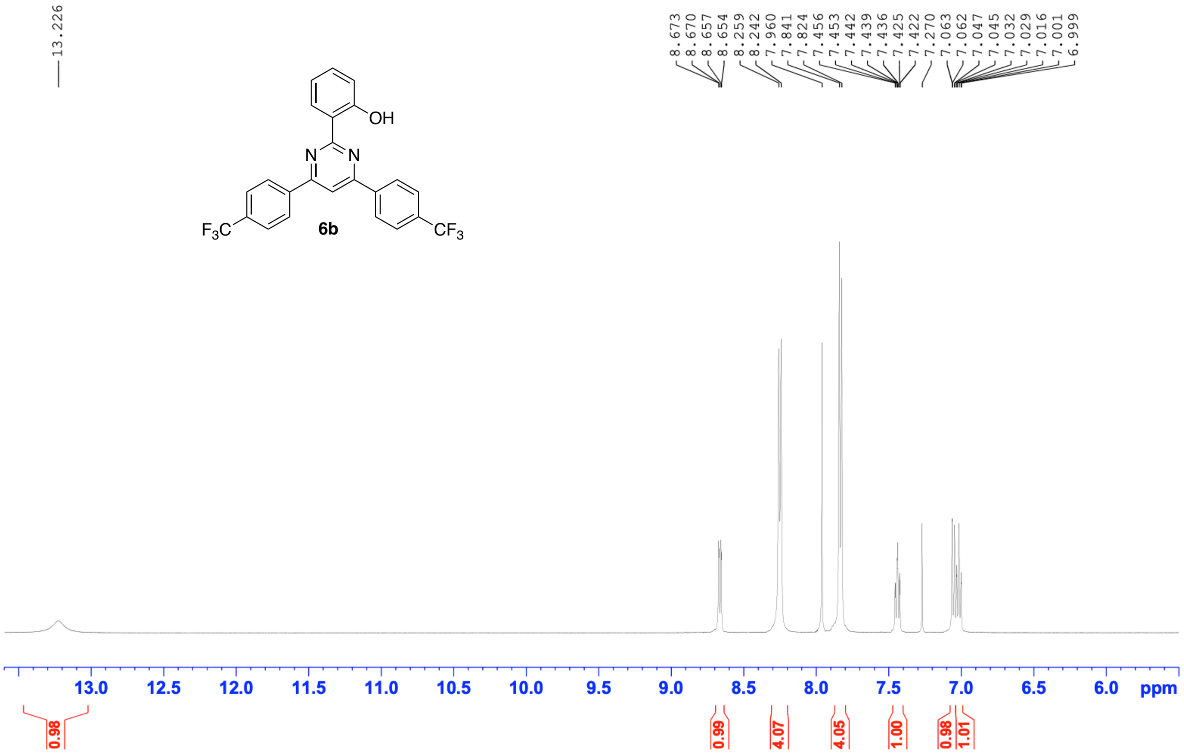

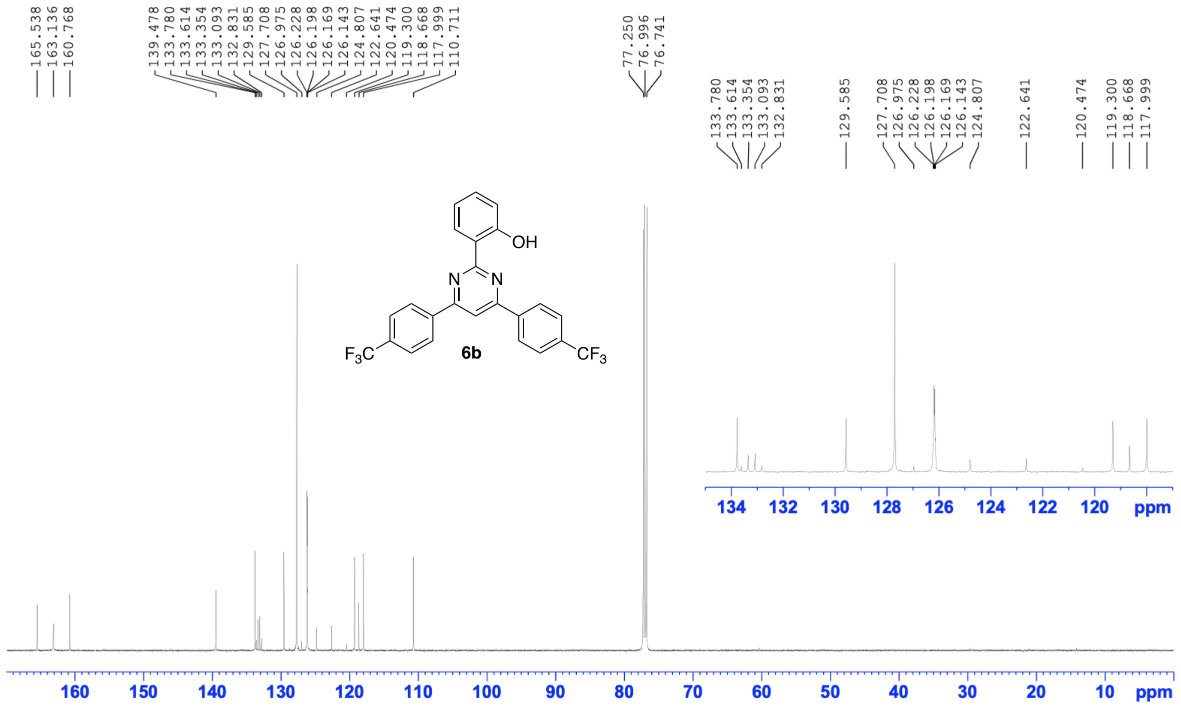

Figure S38. ^{1}H NMR ($CDCl_3$, 500 MHz) and ^{13}C NMR spectra of ($CDCl_3$, 125 MHz) of **6b**.

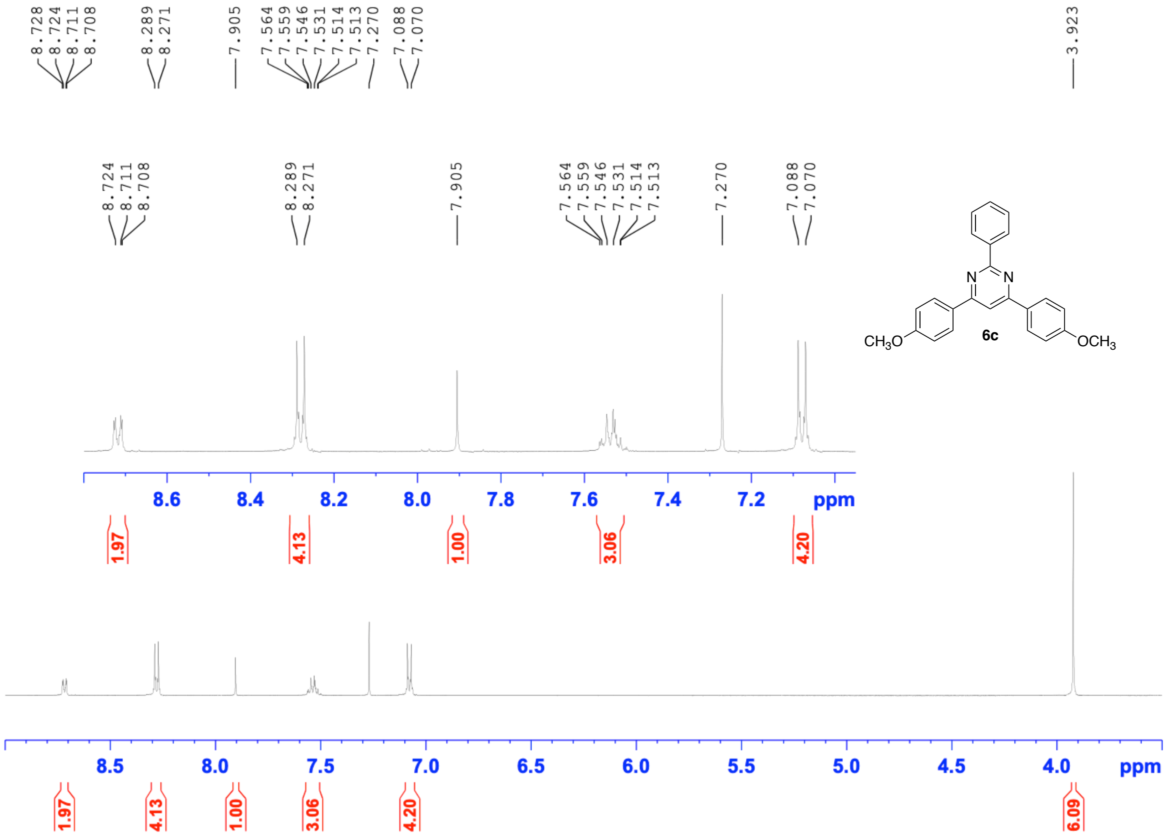

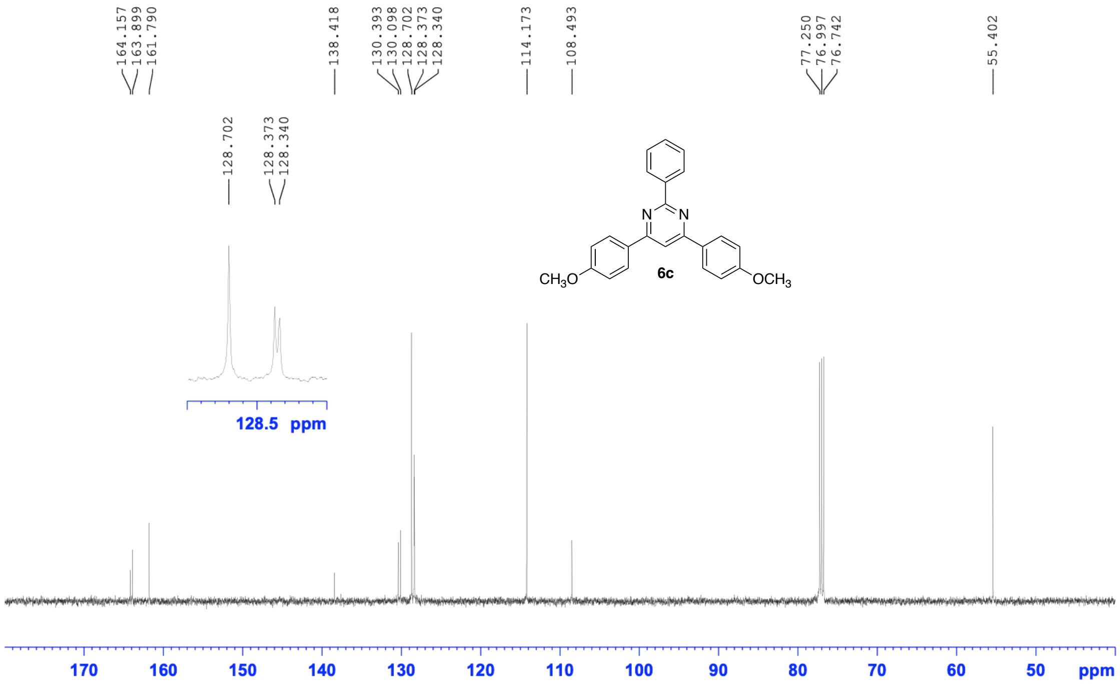

Figure S39. ^{1}H NMR ($CDCl_3$, 500 MHz) and ^{13}C NMR spectra of ($CDCl_3$, 125 MHz) of **6c**.

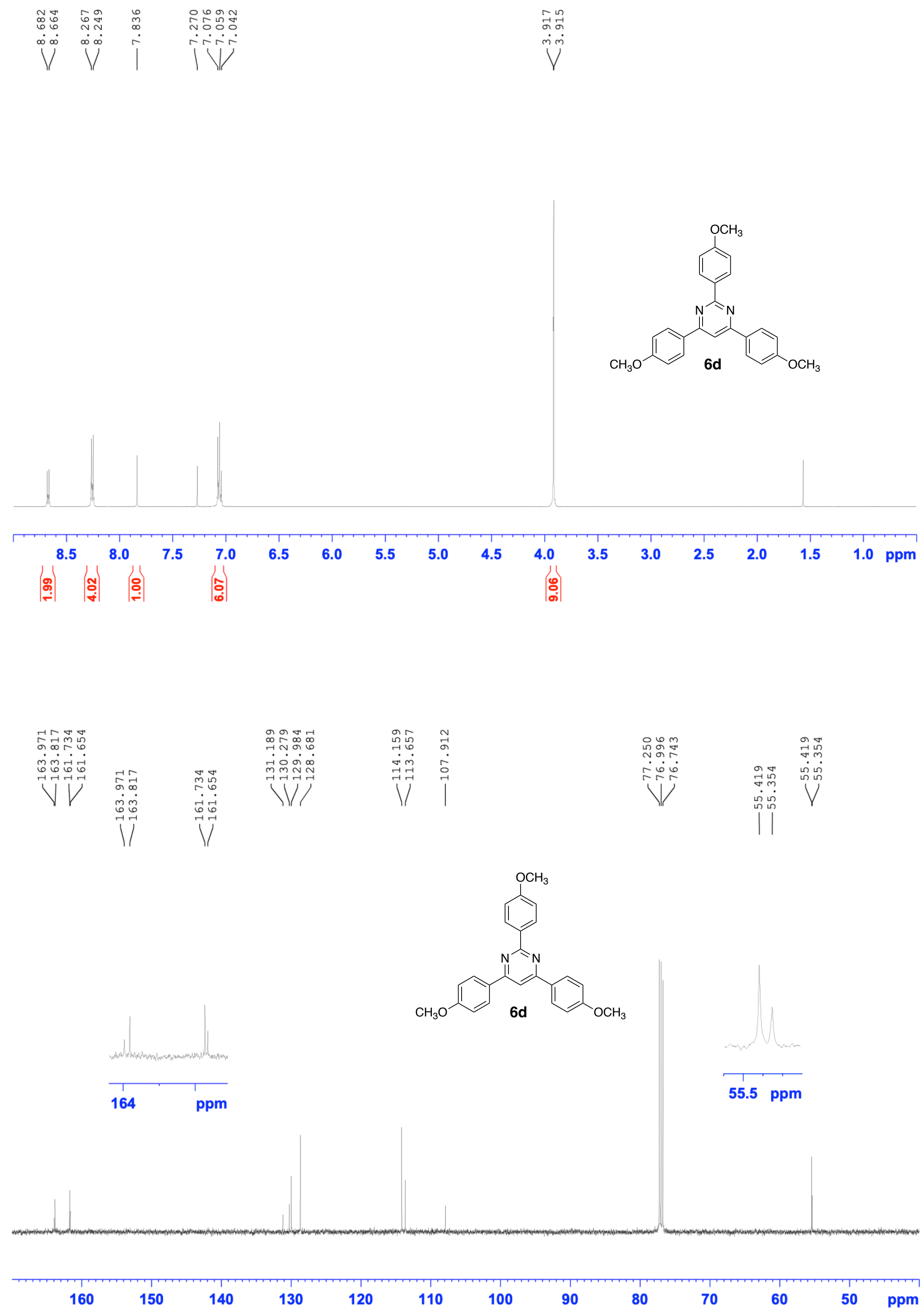

Figure S40. ^{1}H NMR ($CDCl_3$, 500 MHz) and ^{13}C NMR spectra of ($CDCl_3$, 125 MHz) of **6d**.

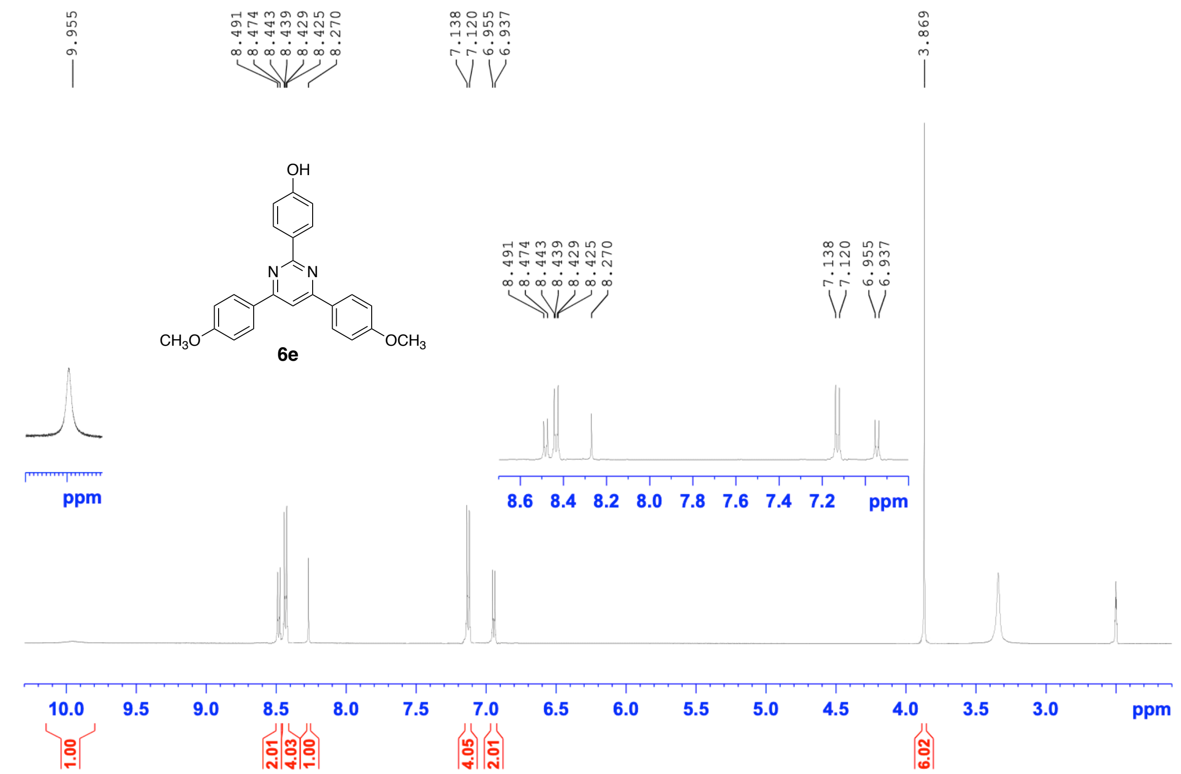

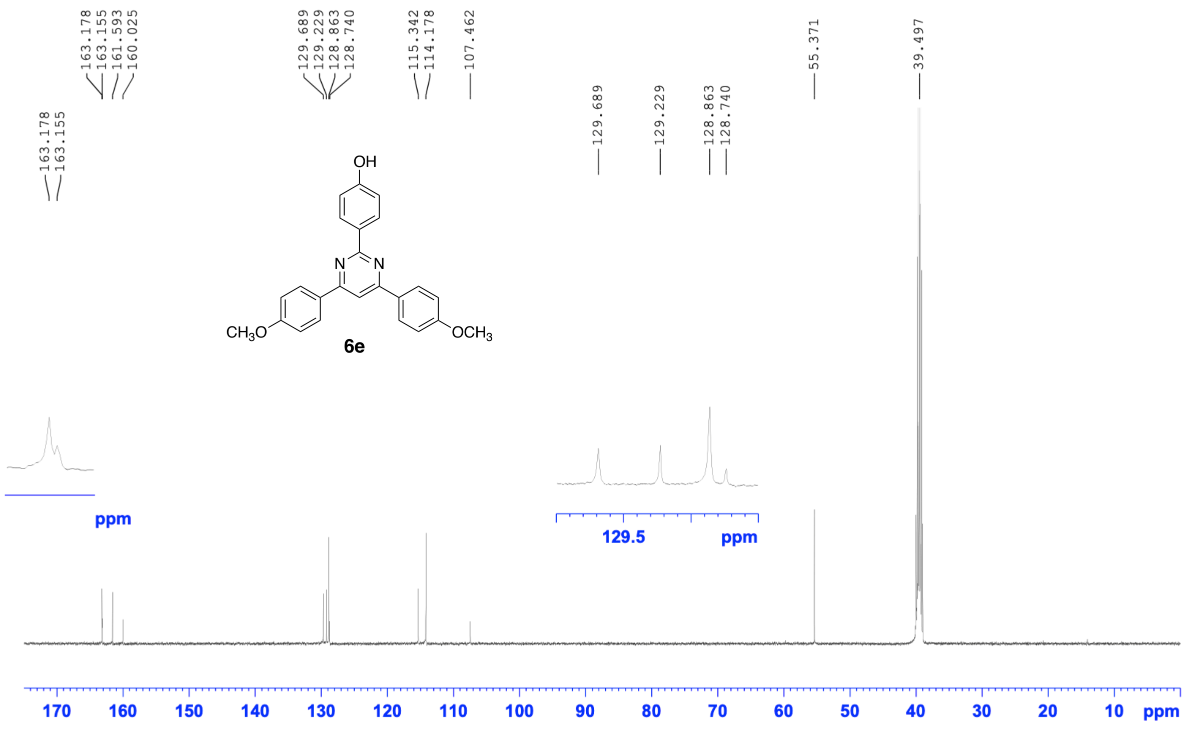

Figure S41. ^{1}H NMR (DMSO-d$_6$, 500 MHz) and ^{13}C NMR spectra of (DMSO-d$_6$, 125 MHz) of **6e**.
